# Correction: One-Seeded Fruits in the Core Caryophyllales: Their Origin and Structural Diversity

**DOI:** 10.1371/journal.pone.0130783

**Published:** 2015-06-19

**Authors:** The PLOS ONE Staff

## Notice of Republication

This article was republished on May 27, 2015 to correct words that were inadvertently merged during the typesetting process. The publisher apologizes for the errors. Also, grammatical errors, hyperlinks and the author contributions were corrected. Please download this article again to view the correct version. The originally published, uncorrected article and the republished, corrected article are provided here for reference.

## Supporting Information

S1 File Originally published, uncorrected article. (PDF)

S2 File Republished corrected article. (PDF)
