# Supplementary material for: One-Seeded Fruits in the Core Caryophyllales: Their Origin and Structural Diversity
Source: PLoS One. 2015 Feb 24;10(2):e0117974. doi: 10.1371/journal.pone.0117974 (PMC4339201; doi:10.1371/journal.pone.0117974)
Supplement: S1 Appendix — (DOC) [file pone.0117974.s001.doc]

**Appendix S1.**Origin of the material used in the carpological investigation in the present article

Achatocarpaceae

*Achatocarpus bicornutus* Schinz & Autran: Paraguay, Dep. [Presidente] Hayes, X.1988, A. Realde *72* (G-357949);

*A. hasslerianus* Heimerl: Paraguay, Parana, 1910, K. Fiebrig *6139* (G);

*A. microcarpus* Schinz et Autran: Paraguay, Ypacarai, 1913, E. Hassler *11457* (E);

*A. nigricans* Triana: Salvador, Santa Ana Dept., Metapan, IV.2007, N. Herrera *s.n.* (BM);

*A. oaxacanus* Standl.: Nicaragua, Boaco Dept., VII.1983, A. Grijalva & M.V. Sandino *2779* (BM);

*A. pubescens* C.H. Wright: Ecuador, Prov. del Guayas, El Progreso, II.1955, E. Asplund *15316* (G);

*Phaulothamnus spinescens* A. Gray: Mexico, Baja California, Isla Coronado, XI.1961, A. Carter *4336* (G-276949);

Amaranthaceaes.str.

*Achyranthes bidentata* Blume: Central Nepal, Kathmandu valley, IX.2010, A. Sukhorukov *594* (MW);

*A. porphyristachya* Wall. ex Moq.: China, Yunnan prov., Hunhe river, XI.1958, A. Voronov *s.n*. (MW);

*Aerva javanica* Juss.: Ethiopia, Ogaden [Region], P.E. Ellis *43* (K);

*A. sanguinolenta* Blume:China, Sichuan prov., IX.1976, anonym *s.n*. (PE-0084841345);

*Allmania nodiflora* (L.) R. Br.: India, Madras, 1924, J.S. Gamble *s.n*. (E) sub *A. longipedunculata*;

*Alternanthera nodiflora*R. Br.: Mali, Katibougou, XI.1966, N.N. Kaden *35* (MW);

*A. pungens* Kunth: India, Assam, IV.1961, Sen & al. *24082* (MHA);

*Amaranthus blitum* L.: Nepal, Kathmandu valley, Godawari, XI.2005, A. Sukhorukov *s.n.* (MW);

*A. muricatus* (Moq.) Hieron.: Australia, Victoria, Mallee distr., III.1967, R.V. Smith *s.n*. (K);

*A. viridis* L.: Israel, Dead Sea area, Ein Boqeq, XII.2008, A. Sukhorukov *s.n*. (MW);

*Baolia bracteata* Kung & G.L. Chu: China, border of Sichuan and Gansu prov., IX.1973, G.L. Chu & al. *15620* (PE-24032);

*Blutaparon portulacoides* (A. St.-Hill.) Mears: [Brazil] Conceiçâo da Barra, VIII.1987, G. Hatschbach & A.C. Carvi *51430* (PE);

*B. vermiculare* (L.) Mears: Senegal, Casamance, XII.1973, P. Hainard & G. Tcheremissoff *21* (G-432814);

*Bosea amherstiana* Hook. f.: 1) N India, Punjab, Wangtu, VII.1934, N. Parmanand *422* (E); 2) Himalaya [without country], Punang, 1890, anonym *s.n*. (E);

*B. cypria* Boiss. ex Hook. f.: Cyprus, Lekfa, X.1940, anonym *1983* (E);

*B. yervamora* L.: [Spain], Gran Canaria, IV.1981, P.H. Davis *67272* (E);

*Celosia trigyna* L.: Saudi Arabia, Asir [prov.], X.1984, A.K. Nasher *198* (E);

*Centemopsis gracilenta* (Hiern) Schinz: Rhodesia, Salisbury [Zimbabwe, Harare], II.1917, F. Eyles *680* (E);

*Chamissoa altissima* Kunth: 1) Mexico, I.1926, J. Ortega *6130* (E); 2) Cuba, prov. Santiago, IV.1982, L. & A. Elenevsky *s.n*. (MW);

*Charpentiera australis* Somer: Tahiti, Rairua, XI.1983, J. Florence *5874* (K);

*C. obovata* Gaudich.: USA, Hawaii, VI.1928, O. Degener *8820* (G);

*Cyathula capitata* Moq.: Central Nepal, Tikhedhunga vill., XI.2008, A. Sukhorukov *s.n*. (MW);

*C. tomentosa* Moq.: Central Nepal, Langtang, IX.2011, A. Sukhorukov (alcohol material);

*Cyphocarpa angustifolia* Lopr.: Botswana, Ghanzi & Kgalagadi distr., III.1980, C. Skarpe *S-445* (K);

*Deeringia amaranthoides* (Lam.) Merr.: India, Calcutta Bot. Garden, 1912, A. Ivanovich *s.n.* (LE);

*D. mirabilis* (Eggli) Appleq. et D.B. Pratt: Madagascar, [Ihorombe region] Ihosy, I.2003, W.L. Applequist & al. *s.n*. (K);

*Digera muricata*(L.) Mart.: 1) India, Delhi, University area, IX.1957, A. George *13* (MW); 2) Yemen, Hays, III.1981, A.G. Miller *3098* (E);

*Froelichia gracilis* (Hooker) Moq.: USA, Missouri, St.-Louis, VII.1969, W.G. D’Arcy *3513* (MW);

*F. drummondii* Moq.: USA, Texas, X.1978, H.H. Iltis, A. Lasseigne *s.n*. (MHA);

*Gomphrena dispersa* Standl.: Cuba, prov. Santiago, XI.1981, L. & A. Elenevsky *s.n.* (MW);

*Hemichroa pentandra* R.Br.: Australia, Victoria, II.1906, H.B. Williamson *s.n*. (E);

*Mechowia grandiflora* Schinz: SW Africa, Zambezi, I.1900, H. Baum *634* (E).

*Pandiaca heudelotii* (Moq.) B.D. Jacks.: Mali, Bamaco, VIII.1967, N.N. Kaden *345* (MW);

*Pleuropetalum pleiogynum* (Kuntze) Standl.: Costa Rica, Puntarenas, III.2000, R. Aguilar *6105* (G);

*P. sprucei* Standl.: Bolivia, 1901, Arnoldi *s.n*. (LE);

*Pleuropterantha revoilii* Franch.: Somalia, Gardo, XII.1969, J.I. Lavranos *7235* (E);

*Polycnemum arvense* L.: 1) [Armenia] Ashtarak, Aragac, X.1974, V. Vašak (B); 2) Russia, Belgorod, VIII.2011, A. Sukhorukov & M. Kushunina *s.n*. (MW);

*Pseudoplantago friesii* Suess.: Argentina, Dept. Eldorado, Posadas to Iguazu, II.1992, T.M. Pedersen *15792* (G-44456);

*Ptilotus fusiformis* (R.Br.) F.Muell.: Australia, Queensland, Venture Creek Crossing, IV.2009, K.R. McDonald *8382* (PE-01873790);

*P. obovatus* (Gaudich.) F. Muell.: W Australia, Morawa, X.2001, R.K. Brummit & B.R. Maslin *20533* (K);

*Pupalia lappacea* (L.) Juss.: Indonesia, Bali, II.2009, V.D. Bochkin & N.A. Bokal *s.n.*(MHA);

*Quaternella confusa* Pedersen: [Brazil] Mato Grosso do Sul, V.2002, G. Hatschbach & al. *73312* (G);

*Saltia papposa* (Forssk.) Moq.: S Yemen, Aden, VIII.1987, L. Boulos et al. *16548* (K);

*Sericocomopsis pallida* Schinz: Ethiopia, Gamu-Gofa region, I.1998, I. Friis & al. *s.n*. (K);

*Sericostachys scandens* Gilg & Lopr.: Equatorial Guinea, Bioco, XI.1986, J.F. Casas *s.n*. (PE-00160239);

*Tidestromia oblongifolia* (S. Watson) Standl.: USA, California, Inyo co., XII.1933, L.S. Rose *33425* (K);

Asteropeiaceae

*Asteropeia densiflora* Baker: Madagascar, Ambatomainty, I.1912, H. Perrier *10143* (BM);

*A. multiflora* Thou.: Madagascar, Toamasina, VI.2009, S.H.J.V. Rapanarivo & al. *s.n*. (K-000664155);

Basellaceae

*Anredera brachystachya* (Moq.) Goverts: St.-Petersb. Bot. Garden (taken from the Greenhouse);

*A. cordifolia* (Ten.) Steenis: USA, Iowa, Montezuma, XI.1961, F.R. Brounell sub *Boussingaultia gracilis* (LE);

*A. scandens* (L.) Sm.: Mexico, Morelos state, X.1902, C.G. Pringle *11151* (E);

*Basella alba* L. s.l.: 1) ex herb. Fischer, IX.1826 *s.n*. (LE); 2) St.-Petersburg Bot. Garden, in cultivation, 1841 anonym *s.n*. (LE); 3) Nepal, Mahakali Zone, Kanchapur distr., XII.1966, D.H. Nicolson *2848* (LE);

*B. paniculata* Volkens: Kenya, Nairobi, [without data] B.R. Adams *59* (K);

*Tournonia hookeriana* Moq.: [Ecuador] Prov. Tungurahua, Cordillera de Llanganates, XI.1939, E. Asplund *9755* (G);

*Ullucus tuberosus* Caldas: Bolivia, Incachaca, VIII.1950, W.M.A. Brooke *6777* (BM);

**Chenopodiaceae**

*Aphanisma blitoides* Nutt. ex Moq.: [USA], California, II.1889, E. Palmer *s.n*. (E);

*Archiatriplex nanpinensis* G.L. Chu: China, prov. Sichuan, Nanping, IX.1980, G.L. Chu *80040* (PE);

*Axyris amaranthoides* L.: Russia, Samara prov., Togliatti, VIII.2013, A. Sukhorukov *s.n.* (MW);

*Bienertia cycloptera* Bunge: [Turkmenistan] Transcaspia, Ashabad, X.1898, D. Litvinov *388* (MW);

*Cornulaca monacantha* Delile: Israel, W Negev [desert], XII.1982, A. Danin & al. *s.n*. (HUJ);

*Enchylaena tomentosa* R. Br.: Israel, Dead Sea area, Ein Boqeq, XII.2008, A. Sukhorukov (MW);

*Fadenia zygophylloides* Aellen & Townsend: Kenya, Marsabit distr., V.1971, H. van Svinderen *M140* (K);

*Hablitzia tamnoides* Bieb.: Russia, Mineralnye Vody, VIII.1965, Shumnova *s.n.* (MW);

*Halocnemum strobilaceum* M. Bieb.: [Turkmenistan] Ashgabad, XI.1898, D. Litvinov *528* (MW);

*Halostachys belangeriana* (Moq.) Botsch.: Kazakhstan, Kzyl-Orda prov., Novokazalinsk, X.2004, А. Sukhorukov *s.n*. (MW);

*Halothamnus bottae* Jaub. & Spach: Saudi Arabia, Al Masane Camp, XI.1985, J.S. Collenette *5492* (K);

*Oreobliton thesioides* Coss. et Durieu: N Africa, Algeria, V.1882, Reboud (BM);

*Suaeda altissima* (L.) Pall.: Russia, Volgograd prov, Elton, IX.1997, E. Mavrodiev & A. Sukhorukov *s.n*. (MW);

*S. linifolia* Pall.: Russia, Volgograd prov., Elton, X.2010, A. Sukhorukov *s.n*. (MW);

Caryophyllaceae

*Acanthophyllum borsczovii* Litv.: Kazakhstan, Karsakpay distr., Kara-kul, VIII.1929, S. Lipshits *757* (MW);

*A. coloratum* Schischk.: Kyrgyzstan, Osh prov., Tar river, VII.1981, Pimanov & al. *567* (MW);

*A. paniculatum* Regel & Herder: Kazakhstan, West Tien-Shan, VIII.1960, Bogibayeva *5590* (MW);

*Achyronychia cooperi* A.Gray: USA, California, San Quentin [Quintin] Bay, I.1889, E. Palmer *672* (LE);

*Cometes abyssinica* R. Br.: Yemen, Southern Jol, IV.1974, S.A. Risopoulos *54* (BM);

*Corrigiola africana* (Turrill) Ikonn.: Abyssinia, 1844, Schimper *1876* (LE);

*C. andina* Planch. & Triana: Mexico, 1876, J.G. Schaffner *s.n.* (LE);

*C. capensis* Willd.: S Africa, Natal, New Hanover distr., XII.1970, K.D. Gordon-Grey *6298* (E);

*C. littoralis* L.: England, S Devon, Slapton, X.2002, S. Kovalsky & J. Boardman *s.n*. (MW);

*C. telephiifolia* Pourr.: Italia, Sardinia, Gallura, IV.1904, A. Vaccari *790* (MW);

*Drymaria cordata* (L.) Roem. et Schult.: Nepal, Nagarkot, XI.2005, A. Sukhorukov *s.n*. (MW);

*Drypis spinosa* L.: Greece, Epirus, VII.1937, E.K. Balls & W.B. Gourlay *s.n*. (E);

*Eremogone micradenia* (P.A. Smirn.) Ikonn.: Russia, Voronezh prov., Bobrovsky distr., VII.1987, Е. Babayeva*s.n*. (MW);

*Gymnocarpos decandrus* Forssk.: Saudi Arabia, Riyadh, III.1973, S.A. Chaudhary *s.n*. (E);

*G. przewalskii* Maxim.: Xinjiang, Qitai, 18.7.1979, anonym *79-413* (XJBI-00079291);

*Herniaria glabra* L.: Kazakhstan, Almaty prov., Rudnichnyi, VIII.2008, A. Sukhorukov *s.n*. (MW);

*H. hirsuta* L.: 1) France, Champchevrier, VII.1847, Blanchet (MW); 2) Israel, Kefar Adummim, IV.2013, A. Sukhorukov *s.n*. (MW);

*H. olympica* J. Gay: Turkey, Bursa [prov.], Uludag, VIII.1996, Pimanov & Klukov *375* (MW);

*Honckenya peploides* (L.) Ehrh.: Russia, Murmansk prov., Poyakonda, VIII.1993, A. Notov & al. *s.n*. (MW);

*Illecebrum verticillatum* L.: Denmark, Fliskov, VIII.1976, S. Jepponsen & B. Lojtnant *707* (MW);

*Minuartia caucasica* Mattf.: Daghestan, Kala-aul, VI.1931, N. Samsel *s.n*. (MW);

*M. hamata* Mattf.: [Georgia] Bot. Garden Tbilisi, 1939, N.N. Kaden (carpological collection at Dept. Higher Plants, Moscow State Univ. *1837*);

*Ortegia hispanica* L.: Portugal, Póvoa e Meadas, VI.1908, G. Sampaio *s.n*. (LE);

*Paronychia amani* Chaudhri: Turkey, Bursa [prov.], Uludag, VIII.1996, Pimanov & Klukov *382* (MW);

*P. arabica* DC.: Algeria, Chellala, VI.1968, V.P. Bochantsev *346* (LE);

*P.argentea* Lam.: Israel, V.2010, A. Sukhorukov (alcohol material);

*P. capitata* (L.) Lam.: Greece, Saronic Island, IX.2010, A. Seryogin *1146* (MW);

*P. chionaea* Boiss.: Turkey, Ichel, VIII.1996, V.N. Pavlov & al. *204* (MW);

*P. chlorothyrsa* Murb.: Saudi Arabia, III.1990, J.S. Collenette *7358* (E);

*P. kurdica*Boiss.: Karabakh, Stepanakert, VIII.1930, I. Schukin & A. Schukina *s.n*. (MW);

*Pollichia campestris* Soland.: Abyssinien, VII.-IX.1872, J.M. Hildebrandt *654* (LE);

*Pteranthus dichotomus* Forssk.: Israel, road from Be’er Sheva to Eilat, V.2013, A. Sukhorukov *s.n*. (MW);

*Saponaria ocymoides* L.: Switzerland, canton of Valais, VII.1874, anonym *s.n*. (E);

*Schiedea globosa* H. Mann: USA, Hawaii, II.1952, O. Degener *21964* sub *S*. *remyi* (G);

*Scleranthus neglectus* Rochel ex Baumg.: Bulgaria, Sytara Planina, VIII.2005, A. Seryogin & M. Bocharnikov *578* sub *S. perennis* subsp. *marginatus* (MW);

*S. perennis* L.: Russia, Tambov prov., near Rasskazovo city, VII.2009, A. Sukhorukov *s.n*. (MW);

*S. polycarpus* DC.: Azerbaijan, Lenkoran, III.1915, V.L. Pastukhov *s.n*. (MW);

*S. uncinatus* Schur: [Ukraine] Carpat prov., Volovets, VIII.1953, Dementyeva & al. *s.n*. (MW);

*Silene baccifera* Roth: Russia, Kaluga prov., 2009, A. Devyatov (alcohol material);

*Spergulaarvensis* L.: Russia, Tver prov., Kamenka, VIII.1936, N. Sokolova (MW);

*Spergularia salina* J. Presl & C. Presl: Russia, Tambov prov., Kirsanov, VIII.1995, A. Sukhorukov *s.n*. (MW);

*Stellaria monosperma* Buch.-Ham. ex D. Don: 1) India, Rampur, X.1938, K. Ram *2230* (E); 2) Japan, Shimotsuke prov., Tochigi pref., IX.1981, M. Furuse *s.n*. (PE-01104627 sub *var. japonica*);

*Telephium imperati* L.: Marocco, El-Ksiba, VII.1997, S.L. Jury *17536* (BM-000559634);

*T. oligospermum* Steud.: Armenia, Echmiadzin, VII.1928, I. Schukin & A. Schukina *s.n*. (MW);

*T. orientale* Boiss.: Caucasus, Georgia, VII.1871, Sitovsky *s.n*. (MW);

Didiereaceae

*Alluaudia ascendens* (Drake) Drake: Madagascar, Toliara [Tulear], XI.1991, S.T. Malcomber *1111* (LE);

*A. comosa* Drake: Madagascar, Tulear, XI.2002, R. Ranaivojaona *516* (K);

*Calyptrotheca somalensis* Gilg: Somalia, Mogadishu Bot. Garden, III.1970, I. Filatov *s.n*. (LE);

*C. taitensis* (Pax & Vatke) Brenan: Tanzania, Dodoma distr., S. Bidgood & al. *6325* (K);

*Ceraria longipedunculata* Merxm. & Podlech: S Africa, Swaziland, Kaokoveld, VII.1976, Leistner & al. *2419* (K);

*C. namaquensis* Pearson & Stephens: SW Africa, VII.1937, Verdoorn & Dyer *1807* (K);

*Decarya madagascariensis* Choux: Madagascar, Amboasar, II.1967, L. Bernardl *11554* (E);

Limeaceae

*Limeum indicum* Stocks ex T. Anderson: Oman, Seel, V.1982, K.S. Price *165* (E);

Lophiocarpaceae

*Lophiocarpus burchellii* Hook. f.: SW Africa [Namibia], distr. Gobabis, II.1955, B. Winter *2468* (K);

*L. dinteri* Engl.: SW Africa [Namibia], Karibib, I.1934, Dinter *s.n*. (BM);

*L. latifolius* Nowicke: Mozambique, Gaza, II.1959, A.G. Barbosa & F. de Lemos *8389* (K);

*L. polystachyus* Turcz.: SW Africa, Us Rivier, V.1972, W. Giess & M. Müller *12028* (H-1113437);

*L. tenuissimus* Hook. f.: 1) South Africa, Transvaal, Pretoria, Wonderboom, III.1954, I.B. Pole-Evans *s.n.*(H-1738443); 2) South Africa, Transvaal [Vhembe District], Soutpansberg, V.1984, K. Balkwill 1639 (E).

Corbichonia decumbens (Forsk.) Exell: Saudi Arabia, Wadi Kharar, II.1980, J.S. Collenette 1902 (E);

C. rubriviolacea (Friedrich) C. Jeffrey: Namibia, distr. Swakopmund, IV.2006, H. Kolberg & al. 34620 (K);

Macarthuriaceae

*Macarthuria australis* Hügel ex Endl.: 1) Australia, ad fl. Cygnorum, VI.1899, J. Drummond *1671* (LE); 2) Australia, [surroundings of Perth] Bayswater, XI.1900, A. Morrison *s.n*. (BM);

*M. neocambrica* F. Muell.: Australia, New South Wales, V.1909, J.H. Maiden *s.n*. (LE);

Microteaceae

*Microtea debilis* Sw.: Trinitatis [Trinidad & Tobago], anonym *134* (LE);

*M. maypurensis* G. Don: Peru, [anno] 1854, W. Lechler *2288* (LE);

*M. paniculata* Moq.: Paraguay, [prov. Cordillera] Coros de Tobati, I.1903, K. Fiebrig *677* (BM);

*M. portoricensis* Urb.: Puerto Rico, Cabo Rojo, I.1885, I. Urban *717* (LE);

Molluginaceae

*A. littoralis* Adamson: S Africa, IX.1897, Schlechter *11383* (BM);

*A. rigida* Sond.: S Africa, Cap, II.1896, Schlechter *7971* (E);

*Glinus oppositifolius* (L.) A. DC.: India, Mysore state, Hassan distr., IX.1971, K.N. Gandhi *s.n*. (E);

Nyctaginaceae

*Abronia latifolia* Eschscholtz: USA, Oregon, VII.1959, R.L. Taylor, G. Staudt *4254* (MW);

*Acleisanthes chenopodioides* (A. Gray) R.A. Levin: 1) Mexico, Dona Ana co., VIII.1897, E.O. Wooton *408* sub *Selinocarpus chenopodioides* (E); 2) Mexico, Chihuahua, IX.1902, C.G. Pringle *11143* sub *Ammocodon chenopodioides* (K);

*Allionia choisyi* Standl.: Mexico, Charcos, VIII.1934, C.L. Lundell *5124* sub *A. glabra* (K);

*A. incarnata* L.: USA, Nevada, V.1905, L.N. Goodding *2255* (E);

*Andradea floribunda* Alemão: 1) Brasil, Rio de Janeiro, IV.1998, B. Longo & al. *342776* (K); 2) Brasil, Rio de Janeiro, VI.2000, C. Farney, J.C. Gomes *4087* (K);

*Boerhavia diffusa* L.: E Nepal, Itahari, III.2012, A. Sukhorukov *s.n.* (MW);

*B. erecta*L.: Mali, Bamaco, IX.1967, N.N. Kaden *442* (MW); 2) Jamaica, Cornwall, XII.2001, M.F. Gardner & S.G. Knees *6363* (E-00131640);

*B. palmeri* S. Wats.: USA, California, San Diego, [without date] J.S. Brandegee *s.n*. (E);

*Boldoa purpurascens* Cav. ex Lag.: Cuba, Havana, I.1905, A.H. Curtiss *585* sub *Cryptocarpus globosus* (E);

*Bougainvillea campanulata* Heimerl: Bolivia, dept. Santa Cruz, prov. Cordillera, XI.2000, M. Nee *51257* (K);

*B. glabra* Choisy: Nepal, Godawari Botanical Garden, IX.2011, A. Sukhorukov (alcohol material; pericarp well-developed, no seeds due to absence of pollinators);

*B. praecox* Griseb.: Bolivia, dept. Santa Cruz, prov. Cordillera, IX.1985, Michel & al. *380* (K);

*Colignonia scandens* Benth.: Ecuador, Loja Prov., VI.1996, G.P. Lewis & B. Merino *s.n*. (E-00196158);

*Commicarpus arabicus* Meikle: Yemen, Wadi Dahr, XI.1982, R.A. King *52* (E);

*C. boissieri* (Heimerl) Cufod.: Oman, X.1983, R.V. Lawton *2486* (E);

*C. plumbagineus* (Cav.) Standl.: Saudi Arabia, Zaymah, II.1980, J.S. Collenette *1750* (E);

*C. sinuatus* Meikle: Yemen, [San’a’ Governorate] Ar Rawdah, XI.1982, R.A. King *323* (E);

*Cryptocarpus pyriformis* Kunth: Ecuador, prov. El Oro, VI.1943, E.L. Little *6597* (K);

*Cyphomeris gypsophiloides* (M.Martnes & Galeotti) Stand.: Mexico, Coahuila state, 1898, E. Palmer (K);

*Guapira discolor* (Spreng.) Little: Cuba, VI.1941, R.A. Howard *4960* sub *Torrubia discolor s.n*. (LE);

*G. graciliflora* (Mart. ex J.A. Schmidt) Lundell: Brazil, Escola Fazendaria, X.1976, J.A. Ratter & al. *3757* (E);

*G. obtusata* (Jacq.) Little: Brazil, Bahia, I.1998, B. Stannard & R.F. Queiroz *s.n*. (E-00310854);

*G. opposita* (Vell.) Reitz: Brazil, Prov. Parana, Mun. Arapoti, XII.1990, G. Hatschbach & J.M. Silva *54883* (E-00304715);

*G. psammophila* (Mart. ex J.A. Schmidt) Angely: Brazil, State of Mato Grosso, XI.1993, J.A. Ratter & al. *7015* (E);

*Mirabilis albida* Heimerl: USA, Manitou, VIII.1901, F.E. & E.S. Clements *36* sub *Allionia hirsuta* (E);

*M. alipes* (S. Watson) Pilz: USA, Nevada, VI.1913, P.B. Kennedy *1989* sub *Hermidium alipes* (E);

*M. himalaica* (Edgew.) Heimerl: West Nepal, Jumla vill., X.2010, A. Sukhorukov *462* (MW);

*M. laevis* (Benth.) Curran: USA, South California, IX.1908, R. Abrams & E.A. McGregor *s.n*. sub *M. aspera* (E);

*M. odorata* L.: Haiti [without date], leg. Jäger *235* (LE) sub *M. dichotoma*;

*M. prostrata* (Ruiz & Pav.) Heimerl: USA, Texas, IX.1917, A. Ruth *1336* sub *Allionia bracteosa* (E);

*Neea psychotrioides* Donn. Sm.: [Belize] Cayo distr., VII.1970, D.L. Spellman & W.W. Newey *1716* (E);

*N. spruceana* Heimerl: Brazil, Caveira Indio, II.1976, J.A. Ratter & al. *3959* (E);

*Oxybaphus nyctagineus* (Michx.) Sweet: Russia, Belgorod prov., VIII.2011, A. Sukhorukov & M. Kushunina *s.n*. (MW);

*Phaeoptilum spinosum* Radlk.: South Africa, I.1906, Dinter *250* (E);

*Pisonia aculeata* L.: 1) India, Madras, coll. Wight *s.n*. (E); 2) China, Kwangtung, VII.1981, anonym *59176* (PE-0917862); 3) Cuba, prov. Santiago, IV.1982, L. & A. Elenevsky (MW) – in this specimen, the pericarp is found to be multi-layered;

*Pisonia umbellifera* (J.R. Forst. & G. Forst.) Seem.: Taiwan, Pingtung co., IX.1984, Y. Tateishi & al. *18417* (MHA);

*Pisoniella glabrata* Standl.: Mexico, X.1861, M. Bourgeou (LE) sub *Pisonia hirtella*;

*Reichenbachia hirsuta* Spreng.: Paraguay, 1880-1890, T. Morong *169* (K);

*Salpianthus aequalis* Standl.: Mexico, Monte de Charnaco, III.1898, E. Langlassé (G);

**Petiveriaceae**

*Petiveria alliacea* L.: Costa Rica, Puntarenas, X.1987, M. Grayum & R. Wagner *8392* (BM);

**Physenaceae**

*Physena madagascariensis* Thou. ex Tul.: Madagascar, Amber Mountain, III.1880, J.M. Hildebrandt *s.n.*(LE);

*P. sessiliflora* Tul.: Madagascar, Toliara, XI.2004, L.J. Razafitsalama *683* (K);

**Rhabdodendraceae**

*Rhabdodendron amazonicum* (Spruce ex Benth.) Huber: Brazil, Pará, VI.1910, A. Duck *s.n*. (BM);

**Rivinaceae**

*Hilleria latifolia* H. Walter: [Democratic Republic of] Congo, Bonsole, X.1957, Evrard *2708* (E);

*Rivina brasiliensis* Nocca: Botanical Garden MSU, Moscow (living material);

*R. purpurascens* Schrad.: [without locality & date] leg. Meyer *s.n*. (LE);

*Schindleria racemosa* H. Walter: Bolivia, Los Yungas, 1890, M. Bang *s.n*. (LE);

*Trichostigma octandrum* (L.) H. Walter: Ecuador, Pastaza [prov.], II.1990, V. Zak & S. Espinoza *5078* *А* (E-00066333);

Sarcobataceae

*Sarcobatus baileyi* Coville: 1) USA, California, [anno] 1875, J.G. Lemmon *s.n*. (LE); 2) USA, Nevada, Esmeralda co., VI.1986, J.D. Morefield *3943* (LE);

*S. vermiculatus* (Hook.) Torr.: USA, Nevada, Cave Creek, VIII.1908, A.A. Heller *9473* (E);

**Seguieriaceae**

*Gallesia gorazema* Moq.: Brasil, Rio Macauhan, VIII.1933, B.A. Krukoff *5405* (LE);

*Seguieria aculeata* Jacq.: Bolivia, prov. de Andrés Ibáñez, Dept. Santa Cruz, VII.1967, M. Nee *35261* (LE);

Simmondsiaceae

*Simmondsia chinensis* C.K. Schneid.: Israel, near Be’er Sheva, plantations, V.2013, A. Sukhorukov (alcohol material);

Stegnospermataceae

*Stegnosperma cubense* A. Rich.: Mexico, Sinaloa, XII.1957, R. Alava, S. Cook *1554* (E);

*S. halimifolium* Benth.: Mexico, Bahia de Los Angeles, II.1963, J. Henrickson *32767* (WU).
