# Supplementary material for: One-Seeded Fruits in the Core Caryophyllales: Their Origin and Structural Diversity
Source: PLoS One. 2015 Feb 24;10(2):e0117974. doi: 10.1371/journal.pone.0117974 (PMC4339201; doi:10.1371/journal.pone.0117974)
Supplement: S1 Table — (DOC) [file pone.0117974.s002.doc]

Table S1. **List of taxa with GenBank-EMBL accession numbers used in the analyses**

| Taxon | NCBI number | |
| --- | --- | --- |
|  | *rbcL* | *matK* |
| **Achatocarpaceae** |  |  |
| *Achatocarpus praecox* | AY270142.1 | AY514845.1 |
| *Phaulothamnus spinescens* | M97887.1 | AY514846.1 |
| **Adgestidaceae** |  |  |
| *Agdestis* sp. |  | AY042538.1 |
| **Aizoaceae** |  |  |
| *Lampranthus* sp. |  | FN597631.1 |
| *Ruschia* sp. |  | AY042649.1 |
| *Trichodiadema* sp. |  | HQ620893.1 |
| **Amaranthaceae** |  |  |
| *Aerva javanica* | AY270050.1 | AY514793.1 |
| *Aerva lanata* | FR775285.1 | FR775267.1 |
| *Alternanthera pungens* | AY270054.1 | AY514795.1 |
| *Amaranthus blitum* | HM849763.1 | HM850679.1 |
| *Blutaparon vermiculare* | AY270067.1 | AY514798.1 |
| *Bosea yervamora* | AY270069.1 | AY514810.1 |
| *Celosia trigyna* | HQ237459.1 | AY514811.1 |
| *Chamissoa altissima* | AY270073.1 | AY514857.1 |
| *Charpentiera obovata* | AY270074.1 | AY514855.1 |
| *Deeringia amaranthoides* | AY270085.1 | AY514814.1 |
| *Froelichia drummondii* | FR775294.1 | FR775276.1 |
| *Gomphrena elegans* | AY270088.1 |  |
| *Gomphrena globosa* |  | HQ619789.1 |
| *Gomphrena haageana* |  | AY514800.1 |
| *Gomphrena serrata* | AY270090.1 |  |
| *Hemichroa pentandra* | JQ693462.1 |  |
| *Hermbstaedtia glauca* | AY270099.1 | AY514812.1 |
| *Iresine* sp. |  | AY514805.1 |
| *Pleuropetalum sprucei* | AY270117.1 | AY514813.1 |
| *Polycnemum majus* | AY270118.1 | AY514839.1 |
| *Polycnemum perenne* | AY270119.1 |  |
| *Pseudoplantago friesii* | AY270120.1 | AY514820.1 |
| *Pupalia lappacea* | AY270122.1 | AY514858.1 |
| *Sericostachys scandens* | AY270134.1 | AY514819.1 |
| **Anacampserotaceae** |  |  |
| *Anacampseros* sp. |  | HQ620850.1 |
| *Grahamia* sp. |  | AY015273.1 |
| **Asteropeiaceae** |  |  |
| *Asteropeia micraster* | AF206737.1 | JQ844149.1 |
| **Barbeuiaceae** |  |  |
| *Barbeuia* sp. |  | AY042552.1 |
| **Basellaceae** |  |  |
| *Anredera baselloides* | HQ621333.1 | HQ620830.1 |
| *Anredera cordifolia* | AY270147.1 | HM851012.1 |
| *Basella alba* | M62564.1 | JQ844148.1 |
| **Cactaceae** |  |  |
| *Opuntia* sp. |  | AY875369.1 |
| *Pereskia lychnidiflora* |  | AY875358.1 |
| *Pereskia stenantha* |  | AY015276.1 |
| *Pereskia weberiana* |  | AY875357.1 |
| *Pereskia zinniiflora* |  | AY015277.1 |
| *Schlumbergera* sp. |  | AY015343.1 |
| **Caryophyllaceae** |  |  |
| *Arenaria* sp. |  | FJ404824.1 |
| *Cerastium* *glomeratum* | HM849882.1 | JN895359.1 |
| *Dianthus* sp. |  | FJ404832.1 |
| *Herniaria glabra* | AF132091.1 | JN589212.1 |
| *Illecebrum verticillatum* | AY270143.1 | AY514849.1 |
| *Minuartia verna* | JQ933411.1 | JN895264.1 |
| *Polycarpon tetraphyllum* | AY270144.1 | FJ404860.1 |
| *Sagina procumbens* | HM850329.1 | JN895587.1 |
| *Schiedea* sp. |  | FJ404868.1 |
| *Scleranthus annuus* | AY270145.1 | DQ267196.1 |
| *Silene baccifera* | JQ933289.1 | JF956240.1 |
| *Silene dioica* | KC171364.1 | KC171395.1 |
| *Silene latifolia* | KC171363.1 | EF647015.1 |
| *Silene vulgaris* | EF646883.1 | EF547245.1 |
| *Spergula arvensis* |  | JN894814.1 |
| *Stellaria* sp. |  | FJ404875.1 |
| **Chenopodiaceae** |  |  |
| *Agriophyllum squarrosum* | AY270051.1 | AY514827.1 |
| *Anabasis aphylla* | HM131746.1 |  |
| *Aphanisma blitoides* | AY270057.1 | AY514844.1 |
| *Archiatriplex nanpinensis* | HM587580.1 |  |
| *Atriplex hortensis* | FR775290.1 | FR775272.1 |
| *Atriplex patula* | HM849801.1 | AY042550.1 |
| *Axyris amaranthoides* | JX848450.1 |  |
| *Axyris prostrata* | AY270062.1 | HE855600.1 |
| *Bassia prostrata* | AY270104.1 |  |
| *Beta vulgaris* | AY270065.1 | AY514832.1 |
| *Blitum bonus-henricus* | AY270079.1 | HE855613.1 |
| *Blitum virgatum* | AY270081.1 | HE855617.1 |
| *Ceratocarpus arenarius* | HM587594.1 | HE855601.1 |
| *Chenopodiastrum coronopus* | HM587595.1 | HE855636.1 |
| *Chenopodium album* | JX848451.1 | HE855665.1 |
| *Chenopodium anidiophyllum* | AY270042.1 |  |
| *Chenopodium nutans* | HM587600.1 | HE855662.1 |
| *Chenopodium quinoa* | KF319008.1 | HE855652.1 |
| *Chenopodium sanctae-clarae* | AY270043.1 |  |
| *Corispermum puberulum* | JF792799.1 |  |
| *Cycloloma atriplicifolium* | HM587598.1 |  |
| *Dysphania ambrosioides* | HM587599.1 | HE855611.1 |
| *Grayia spinosa* | HM587605.1 |  |
| *Hablitzia tamnoides* | AY270092.1 | AY514825.1 |
| *Halocnemum strobilaceum* | AY270094.1 | AY514842.1 |
| *Halostachys belangeriana* | HM630096.1 | DQ499402.1 |
| *Holmbergia tweedii* | AY270100.1 |  |
| *Krascheninnikovia ceratoides* | AY270105.1 | HE855602.1 |
| *Manochlamys albicans* | HM587607.1 |  |
| *Microgynoecium tibeticum* | AY270107.1 | HE855639.1 |
| *Monolepis nuttalliana* | AY270108.1 | HE855621.1 |
| *Oxybasis urbica* | HM587596.1 | HE855630.1 |
| *Scleroblitum atriplicinum* | AY270044.1 |  |
| *Spinacia* sp. |  | HE855620.1 |
| *Suaeda altissima* | AY270135.1 |  |
| *Suaeda linifolia* | HM630106.1 |  |
| *Suckleya suckleyana* | HM587611.1 |  |
| *Teloxys aristata* | AY270140.1 | HE855612.1 |
| **Corrigiolaceae** |  |  |
| *Corrigiola* *litoralis* | FN868311.1 | FN825767.1 |
| *Telephium* *imperati* | FN868312.1 | FN825768.1 |
| **Didiereaceae** |  |  |
| *Alluaudia* *comosa* |  | HQ620838.1 |
| *Alluaudia* *montagnacii* |  | HQ620841.1 |
| *Ceraria* *fruticulosa* |  | AY875371.1 |
| *Portulacaria* sp. |  | AY875368.1 |
| **Galeniaceae** |  |  |
| *Galenia* sp. |  | AY042589.1 |
| **Gisekiaceae** |  |  |
| *Gisekia* sp. |  | AY042591.1| |
| **Halophytaceae** |  |  |
| *Halophytum* sp. |  | AY514852.1 |
| **Limeaceae** |  |  |
| *Limeum arabicum* | FN868314.1 | FN825770.1 |
| **Lophiocarpaceae** |  |  |
| *Corbichonia decumbens* | FN824475.1 | FN825760.1 |
| *Lophiocarpus* sp. |  | AY042611.1 |
| **Macarthuriaceae** |  |  |
| *Macarthuria australis* | FN824479.1 | FN825765.1 |
| **Microteaceae** |  |  |
| *Microtea debilis* |  | FN597632.1 |
| **Molluginaceae** |  |  |
| *Adenogramma mollugo* | FN824409.1 | FN825690.1 |
| *Coelanthum* sp. |  | FN825759.1 |
| *Glinus lotoides* | FN824413.1 | FN825693.1 |
| *Glischrothamnus* sp. |  | FN825699.1 |
| *Hypertelis* sp. |  | FN825763.1 |
| *Mollugo cerviana* | FN824435.1 | FN825715.1 |
| *Pharnaceum* sp. |  | FN825749.1 |
| *Polpoda* sp. |  | FN825753.1 |
| *Psammotropha* sp. |  | FN825755.1 |
| *Suessenguthiella* sp*.* |  | FN825756.1 |
| **Montiaceae** |  |  |
| *Claytonia* sp. |  | JX456286.1 |
| *Hectorella* sp. |  | EF551350.1 |
| **Nyctaginaceae** |  |  |
| *Allionia choisyi* | FR775286.1 | FR775268.1 |
| *Bougainvillea glabra* | M88340.1 | JQ844141.1 |
| *Guapira* sp. |  | FN597630.1 |
| *Mirabilis jalapa* | HM850179.1 | JQ844140.1 |
| *Neea* sp. |  | FJ037933.1 |
| **Petiveriaceae** |  |  |
| *Gallesia* sp. |  | AY042590.1 |
| *Petiveria alliacea* | AJ402987.1 | GQ429080.1 |
| **Seguieriaceae** |  |  |
| *Seguieria* sp. |  | AY042654.1 |
| **Physenaceae** |  |  |
| *Physena* sp. | Y13116.1 |  |
| **Phytolaccaceae** |  |  |
| *Ercilla* sp. |  | AY042583.1 |
| *Phytolacca americana* | FJ860398.1 | JQ844139.1 |
| **Portulacaceae** |  |  |
| *Portulaca grandiflora* | M62568.1 | EU834751.1 |
| *Portulaca oleracea* | HQ621340.1 | DQ855850.1 |
| **Rhabdodendraceae** |  |  |
| *Rhabdodendron amazonicum* | JQ625835.1 | JQ844136.1 |
| **Rivinaceae** |  |  |
| *Hilleria* sp. |  | AY042601.1 |
| *Ledenbergia* sp. |  | AY042606.1 |
| *Rivina humilis* | M62569.1 | AY514850.1 |
| *Trichostigma* sp. |  | FN597635.1 |
| **Sarcobataceae** |  |  |
| *Sarcobatus vermiculatus* | AY270148.1 | AY042652.1 |
| **Simmondsiaceae** |  |  |
| *Simmondsia chinensis* | FJ860399.1 | AY514854.1 |
| **Stegnospermataceae** |  |  |
| *Stegnosperma halimifolium* | M62571.1 | HQ878442.1 |
| **Talinaceae** |  |  |
| *Talinum paniculatum* | AY875214.1 | AY015274.1 |
| **Outgroups:** |  |  |
| **Droseraceae** |  |  |
| *Drosera* *adelae* |  | AY096121.1 |
| **Plumbaginaceae** |  |  |
| *Limonium caesium* | Z97643.1 |  |
| **Polygonaceae** |  |  |
| *Polygonum aviculare* | HM850273.1 | HM357913.1 |
