# Supplementary material for: Correction: One-Seeded Fruits in the Core Caryophyllales: Their Origin and Structural Diversity
Source: PLoS One. 2015 Jun 19;10(6):e0130783. doi: 10.1371/journal.pone.0130783 (PMC4475014; doi:10.1371/journal.pone.0130783)
Supplement: S2 File — (PDF) [file pone.0130783.s002.pdf]

PLOS | ONE

RESEARCH ARTICLE

One-Seeded Fruits in the Core Caryophyllales: Their Origin and Structural Diversity

Alexander P. Sukhorukov[1]*, Evgeny V. Mavrodiev[2], Madeleen Struwig[3], Maya V. Nilova[1], Khalima Kh. Dzhalilova[1], Sergey A. Balandin[4], Andrey Erst[5¤a], Anastasiya A. Krinitsyna[1]

1 Department of Higher Plants, Faculty of Biology, Lomonosov Moscow State University, Moscow, Russia, **2** Florida Museum of Natural History, University of Florida, Gainesville, Florida, United States of America, **3** Department of Botany, University of Zululand, KwaDlangezwa 3880, KwaZulu-Natal, South Africa, **4** Department of Geobotany, Faculty of Biology, Lomonosov Moscow State University, Moscow, Russia, **5** Herbarium, Central Siberian Botanical Garden, Siberian Branch of Russian Academy of Sciences, Novosibirsk, Russia

¤a Current address: Tomsk State University, Tomsk, Russia
* suchor@mail.ru

CrossMark
click for updates

OPEN ACCESS

Citation: Sukhorukov AP, Mavrodiev EV, Struwig M, Nilova MV, Dzhalilova KK, Balandin SA, et al. (2015) One-Seeded Fruits in the Core Caryophyllales: Their Origin and Structural Diversity. PLoS ONE 10(2): e0117974. doi:10.1371/journal.pone.0117974

Academic Editor: Wei Wang, Henan Agricultural Univeristy, CHINA

Received: October 28, 2014

Accepted: January 2, 2015

Published: February 24, 2015

Copyright: © 2015 Sukhorukov et al. This is an open access article distributed under the terms of the Creative Commons Attribution License, which permits unrestricted use, distribution, and reproduction in any medium, provided the original author and source are credited.

Data Availability Statement: All relevant data are within the paper and its Supporting Information files.

Funding: This work was supported by grants of the Russian Fund for Basic Research (project 14-04-00136-a: phylogeny of Caryophyllales) and Russian Scientific Foundation (project 14-50-00029: carpological investigations). The funders had no role in study design, data collection and analysis, decision to publish, or preparation of the manuscript.

Competing Interests: The work of one of the authors (SK) in a Commercial company does not alter the authors' adherence to PLOS ONE policies

Abstract

The core Caryophyllales consist of approximately 30 families (12 000 species) distributed worldwide. Many members evolved one-seeded or conjoined fruits, but their origin and structural diversity have not been investigated. A comparative anatomical investigation of the one-seeded fruits within the core Caryophyllales was conducted. The origin of the one-seeded fruits and the evolutionary reconstructions of some carpological characters were traced using a tree based on *rbcl* and *matK* data in order to understand the ancestral characters and their changes. The one-seeded fruit type is inferred to be an ancestral character state in core Caryophyllales, with a subsequent increase in the seed number seen in all major clades. Most representatives of the 'Earlier Diverging' clade are distinguished in various carpological traits. The organization of the pericarp is diverse in many groups, although fruits with a dry, many-layered pericarp, consisting of sclerenchyma as outer layers and a thin-walled parenchyma below, with seeds occupying a vertical embryo position, are likely ancestral character states in the core Caryophyllales clade. Several carpological peculiarities in fruit and seed structure were discovered in obligate one-seeded Achatocarpaceae, Chenopodiaceae, Nyctaginaceae, Seguieriaceae and Sarcobataceae. The horizontal embryo evolved in only certain groups of Chenopodiaceae. The bar-thickening of endotegmen cells appears to be an additional character typical of core Caryophyllales. The syncarpy-to-lysicarpy paradigm in Caryophyllaceae needs to be reinterpreted.

Introduction

The Caryophyllales belong to the core eudicots [1] and comprises approximately 12 000 species distributed worldwide [2]. Molecular phylogeny divides the order into two large clades. The first clade, which is often referred to as the core Caryophyllales or caryophyllids, includes taxa in the traditional order circumscription (Caryophyllaceae, Amaranthaceae s.str.,

on sharing data and materials. There are restrictions on sharing of data and/or materials. The authors have added an appropriate sentence about absence of competition interests, and this center does not play a role in the aims of this article. The scientific activity of this center is connected with the new technologies in the production of the natural food, and SK is dealing with diverse scientific efforts like interaction between the plants & insects, investigations of fruits and seeds, seed germination etc.

Chenopodiaceae, Cactaceae, Nyctaginaceae, etc.). The second clade (the non-core Caryophyllales, or polygonids) comprises Plumbaginaceae, Polygonaceae, Droseraceae and several smaller families. Around 30 families are known in the core Caryophyllales and half of them were discovered or confirmed in their familial rank only in the last two decades. Two main clades were recognized in the core Caryophyllales; the so-called 'Earlier Diverging' lineages and the 'Globular Inclusion' clade [3–5]. The first clade comprises the families: Rhabdodendraceae, Simmondsiaceae, Asteropeiaceae, Physenaceae, Microteaceae, Macarthuriaceae, Caryophyllaceae, Achatocarpaceae, Amaranthaceae and Chenopodiaceae (the three last families are often abbreviated as the AAC clade). Here, we accepted the 'Earlier Diverging' lineages as consisting of Rhabdodendraceae, Simmondsiaceae, Asteropeiaceae, Physenaceae and Microteaceae. The 'Globular Inclusion' clade is the sister group to another large clade Achatocarpaceae-Amaranthaceae-Chenopodiaceae+Corrigiolaceae-Caryophyllaceae abbreviated here as AAC+CC clade.

Most representatives of the core Caryophyllales share the following distinctive traits: (1) the presence of betalain pigments [3], [6–8]; (2) anomalous secondary thickening of the root and stem [9], [10]; (3) P-type sieve tube elements [11–13]; (4) androecium structure [14]; (5) pollen embryogenesis [15] and morphology [16], [17]; (6) curved (campylotropous or anatropous) ovules [18]; (7) a seed coat that is made of both exotesta (outer layer, outer ovule integument) and endotegmen (inner layer, inner integument), with predominant development of the exotesta at maturity [19–21]; (8) well-developed perisperm as nutritive tissue in the seed and loss of endosperm in the ripe seed [22], [23]; and (9) seedling anatomy [24].

The number of seeds per ovule is one of the most important characteristics within the core Caryophyllales. Multi-seeded fruits are mostly known to occur in the 'Globular Inclusion' clade and the AAC+CC clade; they are usually dehiscent and dry (capsules), but in some cases are indehiscent, dry or berry-like, derived from the ovules, receptacle, and supporting foliar structures (Cactaceae). One-seeded fruits are widespread in all three major clades and are reported for Achatocarpaceae, Agdestiaceae, Anacampserotaceae, Asteropeiaceae, Basellaceae, Chenopodiaceae, Didiereaceae, Microteaceae, Nyctaginaceae, Petiveriaceae, Physenaceae, Rhabdodendraceae, Rivinaceae, Sarcobataceae, Seguieriaceae, Simmondsiaceae, Caryophyllaceae, part of Lophiocarpaceae and almost all Amaranthaceae. The one-seeded fruits have an ovule inserted at the base [25], [26], and as a rule have a dry and indehiscent pericarp. Completely or partially fleshy fruits occur only in Achatocarpaceae, some Rivinaceae, Chenopodiaceae (especially in the tribe Salsoleae), and Amaranthaceae (*Bosea*, *Deeringia*). The one-seeded fruits are often enclosed in an anthocarp (persistent bracts, bracteoles or perianth) which develops wing-like projections or tubercles, enabling long-distance dispersal (either by wind or by animals). This foliar cover is sometimes coloured and contains water-accumulating tissue (Chenopodiaceae: *Enchylaena*, a part of the gen. *Blitum*; Basellaceae: gen. *Basella*), thus mimicking a fleshy pericarp [27], [28]. The presence of such structures enveloping the fruit, often erroneously classified as part of the pericarp has led to morphological misunderstandings. The fruit of *Ullucus* (Basellaceae) was considered to be succulent [28], [29] but is in fact dry [30]. For some Salsoloideae (Chenopodiaceae), 'half-berry' is the appropriate term for a fruit with a fleshy pericarp in only its upper half [31]. However, this problem still applies to the propagules of *Sarcobatus* (Sarcobataceae) which has wing-like outgrowths, and which are interpreted as genuine fruit [32], or as fruit supported by a calyx-like entity [33–36] or where the outgrowth is labelled as "perianth" [37].

The detailed anatomy of the pericarp and the seed coat in one-seeded fruits of Caryophyllales are still poorly investigated. This is true for the small groups that almost completely lack data, and also for larger families, including Amaranthaceae and Nyctaginaceae. However, Caryophyllaceae have received more attention in studies of evolutionary trends in their fruit structure, and in particular the predominance of one-seeded fruits in the family [38]. Based on

Table 1. The traditional view of evolutionary trends in fruit and seed structure in the Caryophyllaceae (after Devyatov and Ermilova, 2002).

Initial characters	Possible intermediate positions	Final characters
Perianth (calyx) without hypanthium; fruit a capsule; syncarpous gynoecium (with septa between the locules); pericarp with well-expressed topographical zones; ovules numerous; seedcoat testa sculptured	Septa abrupt; gynoecium lysicarp (with one central column having numerous seeds)	Hypanthium present; column abortive; gynoecium pseudomonomerous; fruit dry, one-seeded, indehiscent; pericarp reduced to 1–2 undifferentiated parenchymatous cell layers; ovule single with basal placentation due to disappearence of central column; seedcoat testa without prominent sculpture

doi:10.1371/journal.pone.0117974.t001

widely accepted embryological data, several authors [39–41] suggested that fruit/seed characters became simplified over time (Table 1). Some authors [26] [42] instead hypothesized that the fruit/seed structure became more complex. The indehiscent or irregularly dehiscent fruits in the Caryophyllaceae are now considered to be an ancestral trait retained in Corrigioleae (or Corrigiolaceae) and Paronychieae, with subsequent radiations to multi-seeded fruits inferred in various lineages, including the majority of Caryophyllaceae (Plurcaryophyllaceae clade: see [43]).

The aim of the present study is to determine the origin and evolution of one-seeded fruits and their structural diversity within the core Caryophyllales. Specifically we attempt to (1) reconstruct the origin and diversification of one-seeded fruits with molecular phylogenetic analysis using a combined *rbcl* and *matK* matrix; (2) investigate or ascertain the carpological characters of one-seeded fruits with implications for the recent taxonomy of the core Caryophyllales and to discover the possible peculiarities in the fruit/seed structure within the families in their current circumscription; (3) reconstruct evolutionary history in fine carpological characters to understand their origin and possible changes based on a molecular phylogeny derived from published data for *rbcL* and *matK*.

Materials and Methods

Origin of material and its preparation

The carpology of ~460 representatives of the core Caryophyllales were investigated. About 260 species from Chenopodiaceae were included in previous studies [31], [44–50]. A list of additional plants studied here for the first time is given in S1 Appendix. Specimens were collected by the authors in many parts of Eurasia and Africa and are stored at the following herbaria: B, E, G, H, LE, MW, W, PUC, with simultaneous preservation of the fruits in 70% ethyl alcohol. No specific permissions were required for the locations of the material, and field studies did not involve endangered or protected species (weed species investigated were collected from the families Amaranthaceae, Caryophyllaceae, Chenopodiaceae and Nyctaginaceae in European Russia, Nepal, South Africa and Israel). Other material (mostly fallen fruits) was obtained from herbarium specimens (with permission) and soaked in a mixture of ethyl alcohol, water and glycerine in equal proportions. Anatomical cross-sections were made by hand or with a microtome. For the majority of the families, the pericarp or seed structure did not depend on the topology of the cross-sections. However, we argued that the fully developed pericarp structure in some Amaranthaceae s.str. (Gomphrenoideae, Achyranthoids) and Chenopodiaceae (Salsoloideae) is represented in the upper part of the fruit, while the lower parts are characterized by reduced zones and layers. This topographically dependent inequality of pericarp layers also occurs in one-seeded Caryophyllaceae, as noted by Rohweder [39] and Schiman-Czeika [51]. The most valuable data sources in these three families were therefore obtained from cross-sections made from the upper part of the fruit. For tissue staining, the following solutions were used: 0.2% aqueous toluidine blue or 1% aqueous solutions of Safranin + 1% Light Green in

concentrated picric acid were used for general tissue staining, Sudan IV for revealing fatty substances and Lugol's iodine for revealing starch. To detect crystals, sections were viewed under polarized light. Prior to scanning electron microscopy (SEM), the material was dehydrated in aqueous ethyl alcohol solutions of increasing concentration, followed by alcohol-acetone solutions and pure acetone. SEM observations were made with a JSM-6380 (JEOL Ltd., Japan) at 15 kV after critical point drying and sputtercoating with gold-palladium. The carpological terms used are those of Werker [52].

DNA sequence data for two plastid loci, *matK* and *rbcL*, were taken from the GenBank/EMBL databases (S1 Table), concatenated and analyzed as a single dataset using Maximum Likelihood (ML) (RAxML 7.0.4; [53]) and Bayesian analyses (BI) (MrBayes 3.1.2; [54]), as described in Crowl et al. [55]. Two runs with four chains each (three heated and one cold) were run for 15 million generations; the chains were sampled every 1000 generations with default parameters.The first 1000 Bayesian trees were discarded as burn-in, and posterior probabilities were calculated from the majority-rule consensus (50%) of the remaining trees sampled in both runs. At the end of the runs, the standard deviation of split frequencies between the two runs had fallen to 0.0060 [55] for the alignment strategy. Maximum parsimony reconstructions of the histories of the characters were performed using Mesquite v. 2.75 [56]. The topography of the taxa in the combined tree (Fig 1) is almost the same as in recent investigations [5], [8]. The most significant change concerned *Microtea* and *Macarthuria*. In contrast to the previous data that indicated *Macarthuria* as the sister clade of *Microtea* and other clades (incl. the 'Globular Inclusion' and AAC+CC clades), our analysis recovered *Macarthuria* as the sister group of the 'Globular Inclusion' clade. The genera *Limonium*, *Polygonum* and *Drosera* (polygonids) were chosen as outgroups.

Results

Origin of one-seeded fruits in the core Caryophyllales (Fig 2)

For the analysis, the number of seeds in the fruits were classified into three types: 0—one-seeded fruits, 1—multi-seeded fruits, 2—conjoined (both one- and many-seeded) fruits within an individual.

The one-seeded fruit type is the ancestral character state for all the Caryophyllales (core Caryophyllales + Polygonids).

Earlier diverging lineages. One-seeded fruit type is the initial character state in all the clades of the 'Earlier diverging' lineages (*Rhabdodendron*, *Physena*, *Asteropeia*, *Microtea*). *Simmondsia*, however, as the first lineage, is characterized by a labile seed (1 to 3) number.

AAC+CC clade. The one-seeded fruit type is the ancestral character state for the AAC+CC alliance. Many-seeded fruits mostly evolved in the Caryophyllaceae+Corrigiolaceae families. This tendency is seen in the Corrigiolaceae (gen. *Telephium*) and in the tribe Polycarpaeae. The multi-seeded fruit type is the ancestral character state in the large Plurcaryophyllaceae clade (Sperguleae, Sagineae, Sclerantheae, Arenarieae, Caryophylleae and Sileneae), with rare cases of reversion to a one-seeded character state in *Scleranthus* (Sclerantheae), some *Stellaria* (Alsineae) and (not shown in the tree) in Sileneae (*Silene ampullata*) and Caryophylleae (*Scleranthopsis*, *Saponaria*, and *Acanthophyllum*). In the AAC clade, the conjoined fruit types are the ancestral state only in tribe Celosieae (Amaranthaceae), with a further change to form stable multi-seeded capsules (*Pleuropetalum*).

Globular Inclusion clade. In this clade, all three character states (one-, multi-seeded fruits and the conjoined type) can be considered as equivocal. However, *Stegnospermataceae* and other families (*Limeaceae*, *Corbichonia* + *Lophiocarpus*, *Gisekiaceae*, *Aizoaceae*, *Cactaceae*, *Portulacaceae*, *Talinaceae*, *Molluginaceae* and *Phytolaccaceae* s.str.) are characterized by multi-

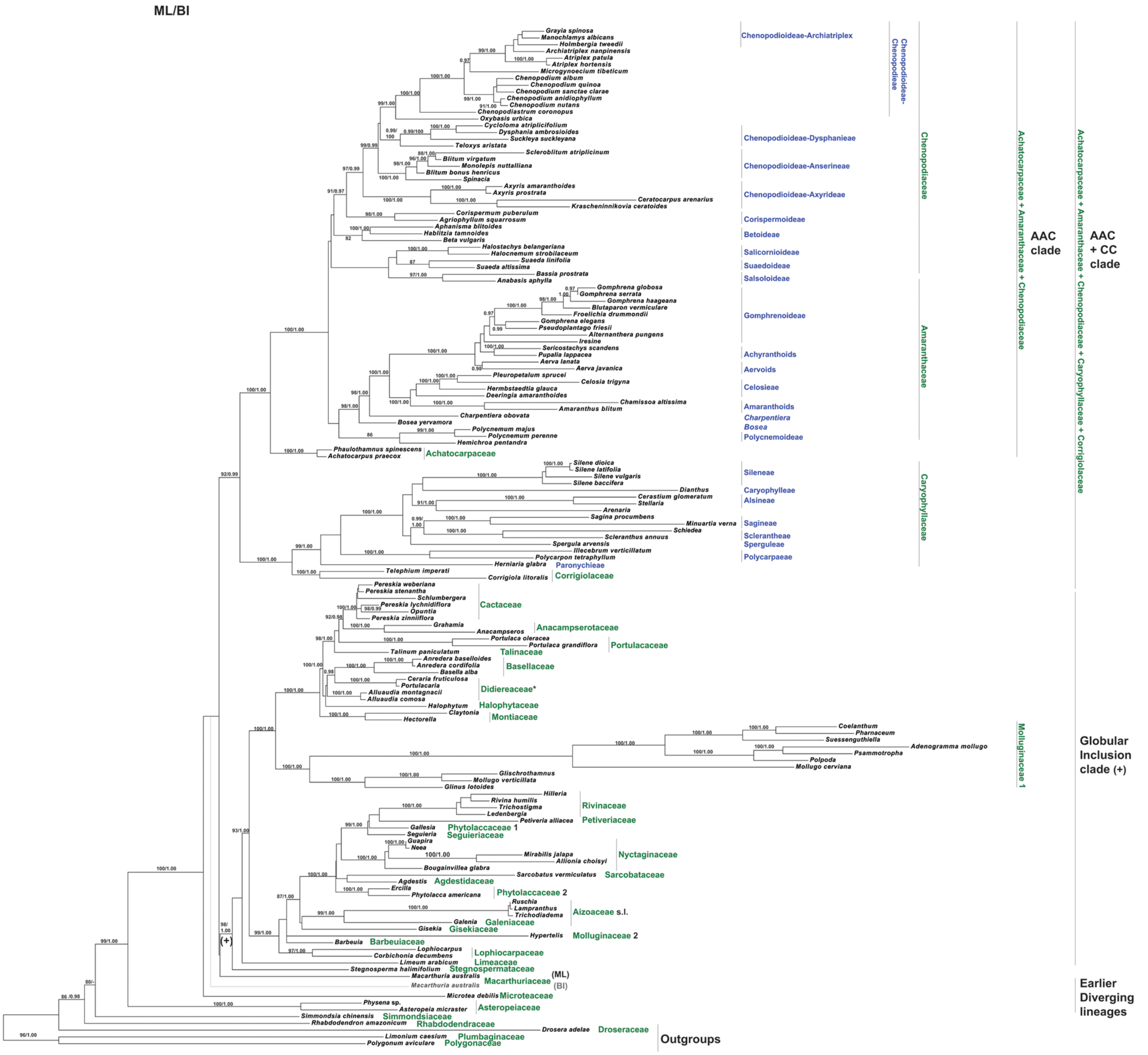

Fig 1. The most probable topology from Maximum Likelihood analysis (ML) of combined plastid dataset. Numbers above branches are bootstrap values >50% posterior probabilities > 0.95 from Bayesian analysis (BI) of the same dataset.

doi:10.1371/journal.pone.0117974.g001

seeded fruits as the ancestral character state. The reversion to a one-seeded fruit type exists in (1) Agdestiaceae + Sarcobataceae, Nyctaginaceae + Rivinaceae + Petiveriaceae + Seguieriaceae, and in (2) Basellaceae + Didiereaceae.

Diversity of the carpological characters in one-seeded fruits

Twenty-six carpological characters of the one-seeded fruits were summarized in this study. Each of these is described to take all the possible variations into account. The data for each investigated taxon are given in the S2 Table.

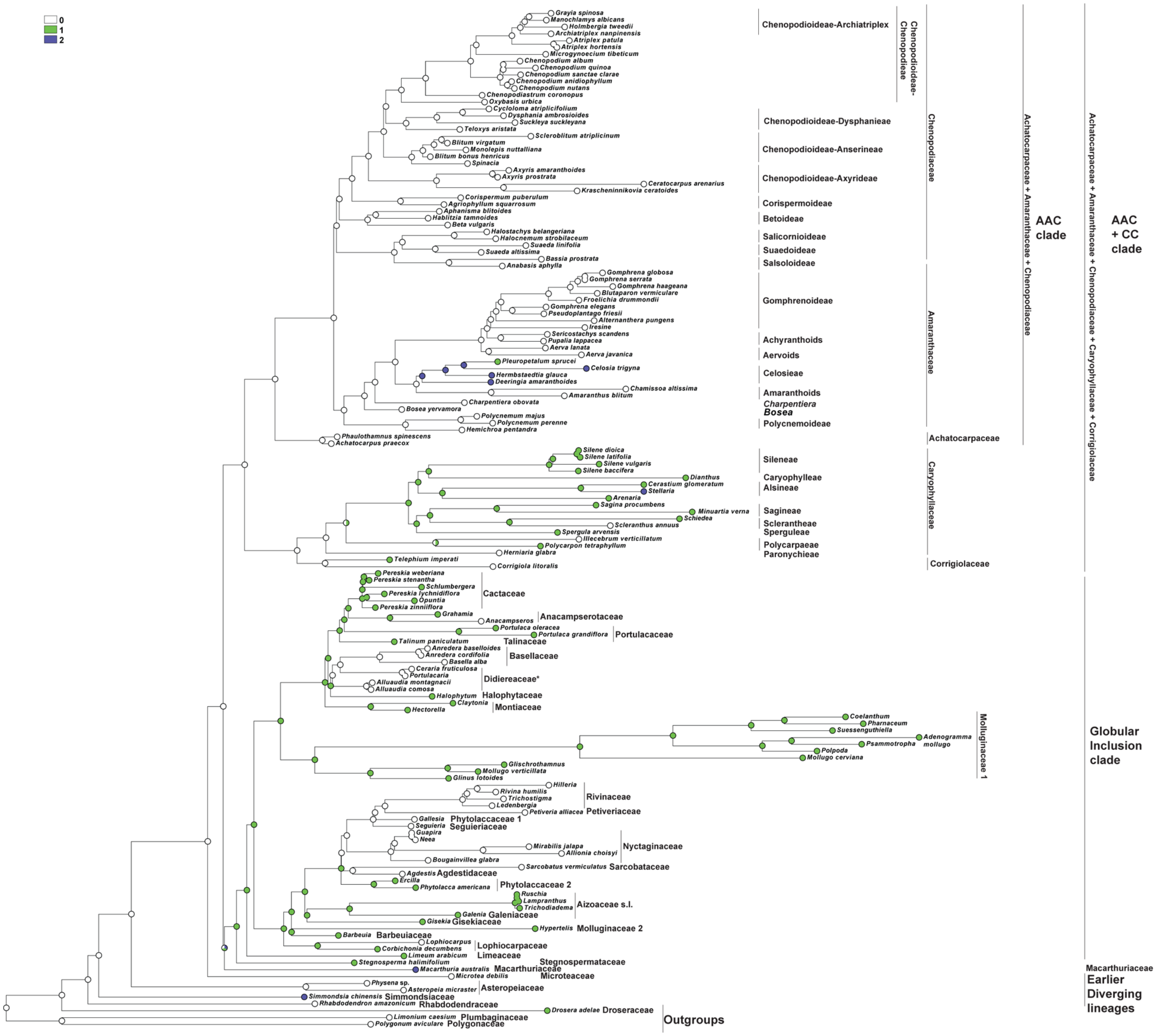

Fig 2. **Maximum Parsimony (MP) reconstruction of the evolutionary history of single-seed state based on combined plastid dataset.** Character states: 0—one-seeded fruits, 1—multi-seeded fruits, 2—conjoined (both one- and many-seeded) fruits within an individual. Morphological characters treated as unordered.

doi:10.1371/journal.pone.0117974.g002

1. Stylodia: 0 –(almost) free; 1—concrescent more than halfway into column, or single stylodium;
2. Fruit length/thickness ratio: 0—almost equal (fruits subglobose or trigonous); 1—length significantly greater than thickness (fruits flattened);
3. Fruit dehiscence: 0—dehiscent in special part; 1—indehiscent or ruptured irregularly (both states can be combined within one individual);
4. Pericarp adherence to the seed coat: 0—easily detached; 1—readily scraped off the seed; 2—pericarp adherent to the seed coat;

5. Wing in the pericarp: 0—expressed only marginally; 1—not expressed; 2—expressed (almost) radially in the pericarp; 3—in the upper part of the fruit (accrescent part of stylodium);

6. Pericarp detachments from the seed coat: 0—visually not noticeable (may be visible anatomically); 1—detachments ear-like, present in the upper fruit part; 2—detachments can evolve in different parts of the fruit;

7. Pericarp succulence: 0—dry; 1—tendency to be fleshy and coloured (or rarely transparent); 2—both dry and fleshy fruits in one individual (due to presence of heterocarpy);

8. Pericarp surface: 0—not rough (not foveolate); 1—clearly foveolate or verrucose;

9. Pericarp layers: 0–1-2(3) layers; 1—more than 3 layers (at least in some fruits due to presence of heterocarpy);

10. Outer cell wall of the outer pericarp layer: 0—papillate (at least in upper part or the majority of fruits if heterocarpous/heterospermous); 1—smooth, not papillate (smooth) or only with mamillae; 2—with vesicular trichomes; 3—with both vesicular trichomes and simple hairs; 4—with stellate hairs; 5—with simple and bifurcate trichomes; 6—with simple hairs; 7—colleters;

11. Pericarp topography: 0—no drastic differences in the consistency of all layers (cells parenchymatous, non-lignified or rarely sclerenchymatic), or only one layer is present; 1—differentiated into parenchyma as outermost layer(s) and sclerenchyma beneath (at least in some fruits, if heterocarpic); 2—differentiated into sclerenchyma (or sclerenchymatic parenchyma) as the outermost layer(s) and thin-walled parenchyma; 3—differentiated into: (a) outer parenchymatous epidermis; (b)—thin-walled parenchyma (but sometimes reduced); (c)—sclerenchyma present as O-shaped cells (with equally thickened walls) or U-shaped cells (unequally thickened walls) that often contain crystals in the protoplast; (d)—inner epidermis (sometimes obliterated); 4—differentiated into: (a) outer sclereid layers; (b) thin-walled parenchyma intermixed with brachysclereids; (c) crumpled parenchyma; (d) inner epidermis; 5—differentiated into: (a) one or several layers with thick walls; (b) thin-walled parenchyma; (c) brachysclereids with walls filled with tannins (fruit is a typical drupe); 6—divided into (a) thick outer epidermis, (b) thin-walled parenchyma, and (c) thick inner epidermis; 7—divided into (a) parenchyma as outermost layer(s), b—sclerenchymatic layer(s), c—parenchyma layer(s); 8—divided into (a) sclerenchymatic parenchyma, (b) thin-walled parenchyma, (c) multilayered fibers; 9—divided into (a) thin-walled parenchyma, b—sclerenchyma, c—thin-walled parenchyma;

12. Presence of a crystalliferous layer with thickened cell walls in the pericarp: 0—absent; 1—present as U-shaped cells in the pericarp (with fine crystalliferous content or small prismatic crystals); 2—present with (almost) equally thickened walls and prismatic monocrystal(s) in the protoplast; 3—present with equally thickened walls and crystals in the form of granules;

13. Exocarp: 0—one-layered only; 1–2-5-layered;

14. Seed colour: 0—black or black and brownish (if heterospermous); 1—red or reddish-black; 2—brown (or yellow-brown); 3—transparent (colourless); 4—red and brown (due to presence of heterospermy);

15. Presence of keel(s) on the seed: 0—absent or slightly keeled; 1—with one or two prominent keels;

16. Differentiation of the seed coat: 0—clearly differentiated into thick testa and thin tegmen layer(s); 1—not clearly differentiated, all layers thin or (almost) equal; 2—both types 0 & 1 (structural heterospermy); 3—differentiated into more than 2 distinct layers;
17. Sculpture of the exotesta: 0—not or slightly undulate; 1—alveolate; 2—with finger-like projections; 3—mamillate or papillate; 4—both 0 and 2 types (heterospermy);
18. Testa thickness: 0—up to 20(25) μm (at least in the majority of the fruits); 1—from 20 to 50(60) μm or more; 2—both types 0 and 1 due to significant structural heterospermy; 3—more than 60 μm;
19. Stalactites in the testa cells: 0—vertically oriented (present in the black and red seeds); 1—obliquely oriented; 2—without prominent stalactites; 3—both types: with vertical stalactites and without them (heterospermy);
20. Protoplast of the testa cells: 0—always compressed (not easily visible); 1—easily visible;
21. Hair-like outgrowths of testa: 0—absent; 1—present;
22. Bar-thickening inseed-coat cell walls (mostly in endotegmen): 0—not detected; 1—present;
23. Seed arillus: 0—absent; 1—present;
24. Embryo orientation: 0—horizontal; 1—both vertical and horizontal within individual plant (spatial heterospermy); 2—vertical; 3—not applicable due to more than two seeds in the fruit or locule;
25. Embryo curvature: 0—annular (curved) or horseshoe-shaped; 1—straight or slightly bent; 2—spiral (twisted) in 1.5–2.5 coils;
26. Perisperm: 0—easily visible; 1—absent, or only traces.

The data presented here provide the most valuable information about the fruit and seed structure of the families investigated. Based on these features, we compared fruit and seed data for many groups of the core Caryophyllales, with further evolutionary reconstructions of several carpological characters using a *plastid-based phylogeny*.

Discussion

Fruit and seed anatomy of the families investigated and its taxonomic importance

Earlier Diverging lineages. Almost all the families of the 'Earlier diverging' lineages—the monotypic Rhabdodendraceae, Simmondsiaceae, Physenaceae and Asteropeiaceae—clearly demonstrate a distinctive carpological structure in contrast to the other members of the order. This is expressed in the possession of a thick pericarp divided into several topographic zones, and in their unusual seed-coat structure. The seed coat typically consists of many layers, but they are all are equal in size. Differentiation into a thick one-layered exotesta and thin one- to several-layered endotegmen was reported to be typical for core Caryophyllales [20], but was not observed in any of our representatives. The perisperm was not found in the ripe seeds.

Rhabdodendraceae. This is a monotypic family with several species in tropical America. Its systematic position within Caryophyllales was based on important anatomical and palynological data [57]. Investigations concerning the characters of *Rhabdodendron* [57–61], however, showed that the genus is distinct in many details from other members of the order. This is also true for its gynoecium structure. The style appears to be in a lateral position (anacrostyly) and

thus the fruit axis is displaced horizontally. Three topographic zones can be distinguished in the fruit: an exocarp with thickened walls, a tangentially elongated thin-walled parenchyma, and a very hard pyrena consisting of many-layered stone cells filled with tannins. The seed coat is brownish and parchment-like, of three to four thin layers, with equal cells having bar-thickened walls. The seed embryo appears horizontal but is actually vertical due to displacement of the fruit axis. Only traces of nutritive tissue are found in the seed.

Simmondsiaceae. This family consists of a single species, *Simmondsia chinensis*, with a narrow distribution in the Sonoran desert [62]. Its systematic position was uncertain (e.g., [63–66]), but many of its anatomical characters do not correspond with families earlier considered to be relatives such as Buxaceae or Euphorbiaceae [20], [67–70]. Recently Simmondsiaceae was placed as diverging near the root of the core Caryophyllales [3], [4]. Only wood anatomy in this family appears to be similar to the other core Caryophyllales and was evaluated as primitive in all dicots [71].

Its fruit and seed anatomy (Fig 3A and 3B) are indeed very unusual within core Caryophyllales. Despite reports that only one seed is present in the capsule [23], [72], sometimes with several rudimentary ovules [73], we also found two or three completely developed seeds in the fruit. Anatomically, the pericarp possesses a one-layered exocarp comprising radially elongated sclereids. Spherical cells of underlying parenchyma (0.7–1.3 mm thick), partially filled with tannin-like substances, form at least 15 chaotically arranged layers. In its lower half, this thin-walled parenchyma is intermixed with brachysclereids. The next few thin layers of parenchyma consist of crumpled, tangentially elongated cells without tanniniferous content. The inner epidermis comprises spherical cells impregnated with tannins. Both seed integuments consist of ~20 layers [20] with no crushing in the ripe seed and cells are filled with tannins. Our investigation confirms previous observations on seed anatomy [20], [23], [67] with some amendments: (1) the number of layers in the dorsal part of the seed is increased in contrast to the ventral surface, and (2) the outer seed-coat layer may consist of both radially elongated and tiny (8–12 μm) thick-walled cells.

The peculiarities of the seed coat are connected with their significant thickness (from 180 μm) and its subdivision into several topographical zones (sclerified layers; loose isodiametric, mesotestal layers; and inner epidermis). The seed cavity is completely filled with a straight embryo that is oriented vertically. The highly diversified and thick fruit and seed covers, the presence of the tannins in many (vegetative and reproductive) organs [68] and liquid wax in the seeds [74] all serve to distinguish *Simmondsia* from almost all other members of core Caryophyllales.

Physenaceae and Asteropeiaceae. These two monotypic Madagascan families were only two decades ago placed in an extended Caryophyllales [75]. The combined *rbcL*/*matK* analysis supports their position within the root of the core order as sister groups [3]. As in *Simmondsia*, the wood anatomy is the more primitive type seen in angiosperms [76]. It also applies to the pericarp of *Physena* and *Asteropeia*, which is robust (0.4–0.6 mm thick in *Physena madagascariensis*, 0.6–0.8 mm in *P. sessiliflora*, and greater than 1 mm in *Asteropeia*), and consists of many layers that are divided into two topographic zones (Fig 3C and 3D): (1) mechanical tissue and (2) thin-walled parenchyma. In both species of *Physena*, the outermost layer consists of radially elongated sclereids with cristate cell cavities that are substituted below by a many-layered sclerenchymatous parenchyma (more than 20 cells). The cells of the mechanical sheath of *Asteropeia densiflora* have even thickness, and they often have a tannin-like content. *Asteropeia multiflora* has an outermost layer of radially elongated cells, and several additional layers with rounded cells. The parenchymatous layers form a robust many-layered zone with discontinuous vascular bundles.

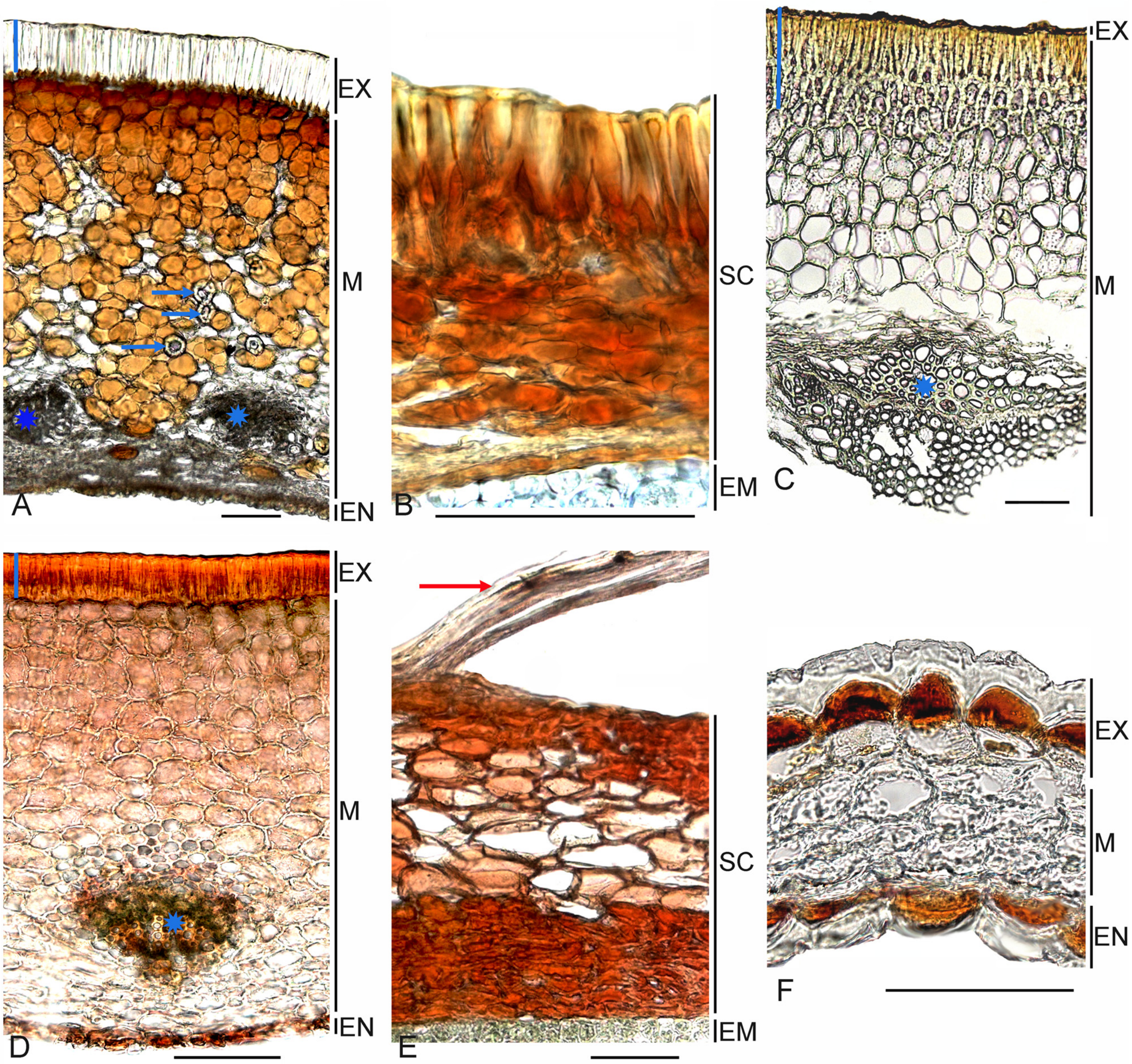

Fig 3. Carpology of the families Simmondsiaceae, Physenaceae and Macarthuriaceae. (A, C, D, F) Transverse sections of pericarp. (B, E) Transverse sections of seed coat. (A, B) *Simmondsia chinensis*, blue arrows indicate brachysclereids, vertical blue line indicates sclereid layer; vascular bundles are marked with asterisk. (C) *Physena madagascariensis*, vertical blue line indicates sclereid layers, vascular bundle are marked with asterisk. (D, E) *Physena sessiliflora*, vertical blue line indicates sclereid layer, vascular bundles are marked with asterisk, red arrow indicates trichome. (F) *Macarthuria australis*. *Abbreviations*: EM—embryo, EN—endocarp, EX—exocarp, M—mesocarp, SC—seed coat. Bars = 100 μm.

doi:10.1371/journal.pone.0117974.g003

The fruit bears only a single ripe seed [77]. The seed coat is especially thick (more than 350 μm) in *Physena* and consists of many layers whose cells are impregnated with tannins (Fig 3E). This robust seed coat looks similar to that in *Simmondsia*, but there are air cavities in the mesotestal layers (*P. madagascariensis*) or hair-like outgrowths of the exotesta cells (*P. sessiliflora*). In contrast, the seed coat of *Asteropeia* is thinner and consists of two to five unequal cell layers that are also filled with tannins. The seed embryo is straight with curved imbricate cotyledons. Only traces of nutritive tissue are found in the representatives of both families.

Macarthuriaceae. It is a recently described monotypic family [78] restricted to Australia that consists of about 10 herbaceous to shrubby species. The systematic position of *Macarthuria* within the core Caryophyllales has been labile [79] (with references herein). Most authors placed the genus in Molluginaceae (e.g. [80]), despite some differences in the general characteristics, including the presence of the funicular seed aril [81]. Recently the genus was included in one of the deepest lineages of core Caryophyllales based on both *rbcL* and *matK* markers [4], [5], [82].

Anatomically, the pericarp of *Macarthuria* is distinct in that both the thick-walled exocarp and endocarp are impregnated with tannins (*M. australis*), and in having a two- to four-layered thin-walled mesocarp (Fig 3F).

Microteaceae. This family was recently established [2] and comprises a single genus with several annual species that are distributed in the tropical parts of America. It is distinguished by its possession of small (1–3 mm), spherical, and indehiscent fruits containing a single seed. The pericarp varies among species: its surface can be smooth or alveolate, or contains prickles or acute outgrowths (Fig 4A and 4B). The presence of projections was one of the reasons used to transfer *M. maypurensis* into a separate genus, *Ancistrocarpus* [83]. However, all investigated *Microtea* species share the same carpological traits: (1) the presence of a parenchymatous pericarp that is tightly adjoined to the seed coat, (2) a thick testa, and (3) a thin tegmen with bar-thickened walls. The embryo is vertical, annular or (in *M. maypurensis*) slightly bent.

Two lineages, *Microtea* and *Macarthuria*, are the first clades sharing carpological characters that are typical for the majority of the members of the order. They have: (1) a relatively small (1.5–5 mm) fruit and seed, (2) a non-multiplicative black seed coat with a crustaceous (thick) single-layered testa (30–50 μm), and one to several barely noticeable endotegmen layer(s), and (3) the central position of the perisperm in the seed and annular embryo shape.

AAC+ CC clade. The Caryophyllaceae alliance (Caryophyllaceae s.str. + Corrigiolaceae)—This alliance consists of ~2200 species distributed worldwide (Caryophyllaceae s.l. in [84]) and is one of the deepest, but highly diversified clades in the core Caryophyllales [3] with some archaic traits in stem anatomy [85]. The former classification of Caryophyllaceae into three subfamilies based on reproductive character sets appears to be unreliable [86], [87]. Further

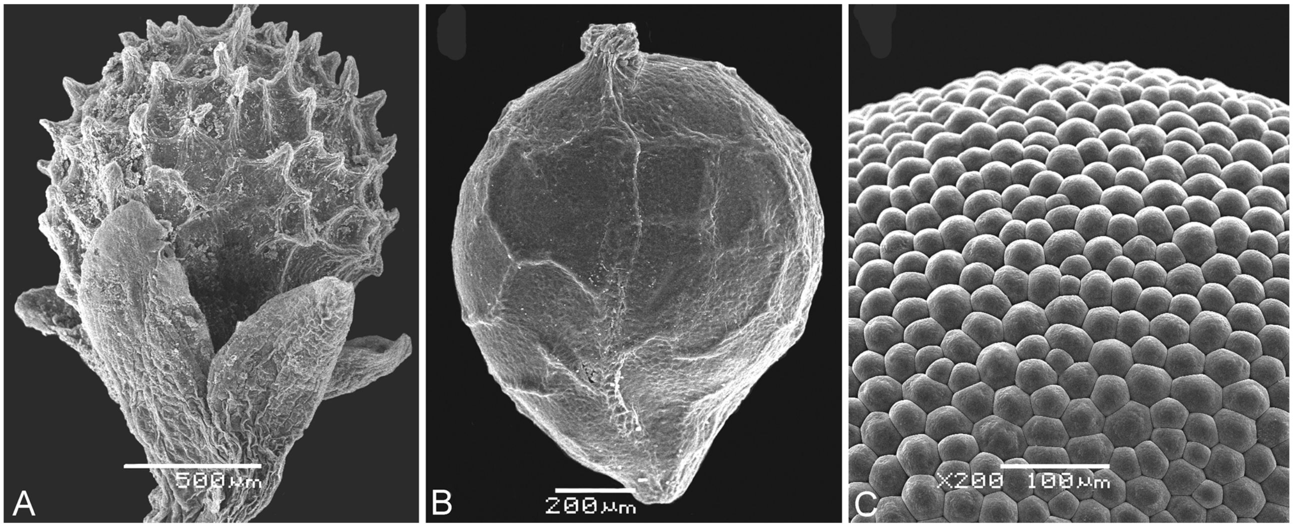

Fig 4. Scan micrographs. (A) *Microtea debilis*, fruit with perianth. (B) *Microtea portoricensis*, fruit. (C) *Telephium imperati*, mamillate seed surface. Scale bar = 500 μm (A), 200 μm (B), and 100 μm (C).

doi:10.1371/journal.pone.0117974.g004

reconstructions of the character states within the new taxonomic rearrangement show that the representatives with the indehiscent or irregularly dehiscing one-seeded fruits first evolved in the deepest clades (Corrigioleae, or Corrigiolaceae; Paronychieae, and partially Polycarpaeae), and the evolution of capsules from a one-seeded fruit type is a common trend in many clades [43]. A tendency of reduction in seed number is assumed in all tribes in which one of several to many ovules becomes the surviving seed (e.g., [39], [51]), but this trend is exceptionally rare in the remainder of Caryophyllaceae (Caryophylleae: *Acanthophyllum*, *Scleranthopsis*, some *Saponaria*, and Sileneae: *Silene ampullata*). Investigations have shown that *Saponaria* species, especially *S. ocymoides*, usually considered as having one seed in the capsule, can in fact develop one to four seeds [88]. Although many members of Caryophyllaceae s.str. are thought to be well-studied carpologically, especially with regard to their seeds, we uncovered additional carpological variation that has not attracted attention so far.

The first clade in this alliance is Corrigiolaceae (or Corrigioleae), consisting of two genera—*Corrigiola* and *Telephium* [43],[89]. We recognize Corrigioleae at the familial level, as proposed by Dumortier [90], based on its position as the sister clade of other Caryophyllaceae s.str. An unusual trigonous pericarp shape is found in both *Corrigiola* and *Telephium* (see also [91]), but *Corrigiola andina* and *C. africana* possess almost orbicular fruits, hardly sharp-edged and then only at the apex. Another peculiarity of both genera is an almost completely sclerified pericarp consisting of many (7–10) layers. The sole exception is *Telephium imperati*, which has two (-three) outermost sclerenchymatous layers. The unlignified cells substitute the sclerenchyma only beneath as 1-3(-5) innermost, often crushed layer(s). However, these genera differ from each other by the fruit type, which can be a many-seeded (dehiscent) capsule (*Telephium*) or a one-seeded utricle (*Corrigola*), contrasting with previous reports of a one-seeded utricle as the only fruit type [43]. Although the fruit of *Corrigiola* is always described as indehiscent, Rohweder [39] stated that its loculicidal ('ribbed') dehiscence occurs only at seed germination.

In contrast to *Corrigiolaceae*, the pericarp of Caryophyllaceae s.str. is usually not very thick, and cell-wall sclerification is found in the epidermis and (facultatively) in one or several subepidermal cells only (as in *Telephium imperati*). A similar thinner pericarp with the same topography is found in multi-seeded fruits in the representatives of Sileneae [92], and in the present study in Sperguleae, Polycarpaeae (*Ortegia*), Sclerantheae (gen. *Schiedea*) as well as in one-seeded fruits of *Minuartia hamata* (Sagineae), *Acanthophyllum* (*Caryophylleae*), and *Cometes* (not included in the molecular analysis). The second type of pericarp zonal division, in *Scleranthus* (Sclerantheae), involves sclerification of the cell walls of the inner layers at the apex of the fruit that is anchored. However, the sclerification of the pericarp is totally absent in most members with one-seeded fruits, and the thin pericarp consists of parenchymatous layer(s) (*Herniaria*, *Anychia*, *Gymnocarpos*, *Pteranthus*, some *Paronychia*: [93]). Rarely, the supporting tissue is absent in many-seeded fruits in which the outermost layer consists of living cells with more or less thickened walls (Sclerantheae: *Wilhelmsia physodes*, *Honckenya peploides*; Sileneae: *Silene baccifera*). In such fruits, which have a parenchymatous pericarp, the innermost layer consists of cells with thick anticlinal and inner periclinal walls, but with no fine crystalliferous content. The parenchymatous pericarp of *Illecebrum verticillatum* consists of parenchymatous cells with bar-thickened walls.

Of all the Paronychieae, *Paronychia* appears to be the most complicated genus. Molecular investigations suggest that *Paronychia* is not monophyletic and is divided into two different clades within the Paronychieae [43]. From a carpological point of view, some species possess a parenchymatous one- to two-layered pericarp (*P. argentea*, *P. amani*), but *P. kurdica*, *P. capitata*, *P. chionaea* and *P. chlorothyrsa* have a thin-walled exocarp and endocarp and sclerified cell walls with a crystal-filled mesocarp.

The seeds are quite diverse. Keeled seeds are rare but are found in *Telephium imperati* (Corrigiolaceae) and *Herniaria* (Caryophyllaceae) species, for example. The seeds can take two forms (especially in Corrigiolaceae): smooth, with equally thick testa and tegmen (*Corrigiola*), or clearly mamillate (Fig 4C), with a thick testa and a thin tegmen (*Telephium*). The smooth pattern is most common in Paronychieae and Sclerantheae, whereas the sculptured (mamillate, alveolate or finger-like) testa is quite well developed in the clades Sperguleae, Arenarieae, Alsineae, Caryophylleae, and Sileneae. However, the representatives of *Acanthophyllum* have only one-seeded fruits that can have various (smooth, alveolate or finger-like) types of seed coat, and *Stellaria monosperma* is characterized by having one-seeded fruits with a smooth seed surface, in contrast to other *Stellaria* taxa whose fruits contain many seeds and have mamillate sculpturing. The majority of the alliance's representatives have curved (annular) embryos, but we agree with Pal [94] that bent or straight embryos are not rare in many taxa in Caryophyllaceae s.str. (e.g. *Achyronychia*, *Polycarpon*, *Illecebrum*, *Pteranthus*, *Pollichia*). One additional embryo type is known in several members: in *Spergula* and *Drypis* [84] it is coiled in 1.5 turns. In spite of different degrees of curvature of the embryo, it is oriented vertically in all the members.

Achatocarpaceae. This family comprises two American shrubby genera: *Achatocarpus* (about 15 species) and the monotypic *Phaulothamnus*. The small female flowers produce translucent berries which turn black when dry. In the past, Achatocarpaceae was considered to be part of Phytolaccaceae which has an uncertain position in the family [95], [96]. The distinct wood anatomy [97] and the absence of anomalous secondary thickening in the stem [9] have supported its familial status, as proposed by Heimerl [98]. The two genera share similar pollen structure [99], [100] and carpological characters described here. The pericarp is divided into four topographic zones: (one-) two- to five-layered outer epidermis, a rare trait in core Caryophyllales, (2) underlying multi-layered soaked parenchyma, (3) one to two layers of U-shaped cells with fine crystalliferous content, and (4) one-layered inner epidermis. The seed coat consists of a robust testa and thinner tegmen that have bar-thickened walls; the embryo is vertical. Abundant starch-granule conglomerates are found in the perisperm, and this carbohydrate type has apparently not been found in other core Caryophyllales so far. In addition to this peculiarity, we note that Achatocarpaceae is the first lineage in AAC clade which has evolved a pericarp with a multi-layered outer epidermis and a layer with U-shaped cells with fine crystalliferous content. A similar multi-layered outer epidermis is also found in the pericarp of restricted Chenopodiaceae members of different taxonomic position: in three *Anabasis* species [31] and *Holmbergia tweedii* [101].

Amaranthaceae s.str. This is one of largest families (~700 sp.) distributed in the Tropics. It is the sister clade of Chenopodiaceae [102]. Most species have one-seeded fruits, with a single exception: tribe Celosieae has several- or many-seeded fruits. The fruit and seed anatomy of Amaranthaceae are poorly investigated. The fruit anatomy is known in the closely related genera *Amaranthus* and *Chamissoa* only. Costea et al. [103] discovered a four- to eight-layered pericarp in some *Amaranthus* consisting of unlignified cells. In *Chamissoa*, the pericarp is composed of both epidermal parenchyma and sclerenchymatous mesocarp [104]. The seed coat is reported to be thick [105]. However, none of these descriptions represents the main carpological structure in Amaranthaceae (see below). The groups in the family are named according to Müller and Borsch [106] whose circumscription is generally confirmed by recent studies [107], [108]. The first grades of Amaranthaceae include subf. Polycnemoideae [109] and the genera *Bosea* and *Charpentiera*.

Subfam. Polycnemoideae. This group was transferred from Chenopodiaceae to Amaranthaceae [102], [107] and consists of four small genera [109] with a disjunct range pattern: *Nitrophila* (America), *Polycnemum* (temperate Eurasia + North Africa), *Hemichroa* and *Surreya*

(both from Australia). *Polycnemum* and *Hemichroa* are investigated here, and many traits (including pericarp outlines, pericarp adherence to the seed coat) are found to be diverse (S2 Table). Both genera, however, have a homocellular parenchymatous pericarp and black seeds that have a more or less hard testa, with stalactites in the outer cell wall (Fig 5A). One peculiarity in *H. pentandra* is that one seed margin is keeled and the other is rounded.

Bosea. This is a genus of three shrubby species with a small and disjunct range pattern: *B. amherstiana* occurs in NW Himalaya, while *B. cypria* and *B. yervamora* are found in the Mediterranean area (Cyprus and Canary Islands, respectively). There are no differences in their carpological characters. The fruits are red-coloured berries with a multilayered pericarp that consists of (1) a one-layered outer epidermis; (2) a robust parenchyma that accounts for the fruit's fleshiness; (3) U-shaped cells with fine crystalliferous content, and; (4) an inner epidermis. There are no data on the dispersal of *Bosea* fruits [110],[111], but they should be well-adapted to bird dispersal, as they have an attractive pericarp color and a hard testa 30–100 μm thick that should protect the embryo after digestion (Fig 5B). The embryo is mostly curved, but can be almost straight in *B. yervamora*.

The main pericarp type in Amaranthaceae (*Bosea*, *Charpentiera*, Achyranthoids, Gomphrenoideae, Celosieae, and some Amaranthoids) is observed to have zonality. Only a few details distinguish the pericarp of *Bosea* from most other representatives of the family. It has a thick subepidermal parenchyma that is colored, and a robust seed coat. Together with morphological data [112], [113], these carpological results confirm the similarity of *Bosea* to Amaranthaceae and not to Anacardiaceae that is proposed by Kunkel [114], the members of which have other traits in fruit/seed structure, as studied by Plisko [115].

Charpentiera. Eight tree-like species are locally distributed in Polynesia [116]. Two species investigated here have a dry, thin pericarp of four to five layers (*C. obovata*) or many layers (6–10 layers in *C. australis*), but with the same topography as *Bosea*. The seeds have a crustaceous testa (50–60 μm) with stalactites in the outer cell walls and compressed protoplast. The embryo is sometimes underdeveloped ([117], and present investigation).

Amaranthoids (*Amaranthus*, *Chamissoa*, *Pleuropterantha*). In general, all three genera have similar pericarp structures that differ only marginally. The core genus *Amaranthus* often possesses a pericarp with an alveolate or rough surface, and its mesocarp has radially elongated, parenchymatous cells located in three to eight layers, often with large intercellular spaces [103]. The pericarp of *Chamissoa altissima* consists of several (four to six) layers that are tightly packed but without any intercellular cavities. In contrast to a previous investigation [104], we found that this sclerenchymatous layer (the innermost layer of mesocarp) located above the inner (often crushed) pericarp epidermis consists of large U-shaped cells with a fine crystalliferous content. The innermost mesocarp layer of *Pleuropterantha revoilii* is distinguished in having equally thickened (O-shaped) cells with monocrystals (Fig 5C). The monocrystals are not found in *Amaranthus* or *Chamissoa*, but scattered cells with a fine crystalliferous content in the upper part of the fruit are present in some members of *Amaranthus* (e.g., *A. muricatus*, *A. viridis*).

The seeds mostly have a thick, crustaceous testa with vertical stalactites in the outer cell walls (*Amaranthus*, *Chamissoa*). In some *Amaranthus*, the evident structural heterospermy is evolved with predominating one of two (dark or yellow) seed types, the latter lacking stalactites. *Pleuropterantha* is distinguished by having thin, almost equal seed coat layers that lack stalactites in the testa cells.

Achyranthoids (of which *Achyranthes*, *Centemopsis*, *Cyphocarpa*, *Mechowia*, *Cyathula*, *Pandiaka*, *Sericocomopsis*, *Sericostachys*, *Pupalia* were studied). The pericarp structure is the main pericarp type of Amaranthaceae (i.e. a parenchymatous exocarp, subepidermal parenchyma facultatively present when the pericarp layers are more than two, crystalliferous layer with

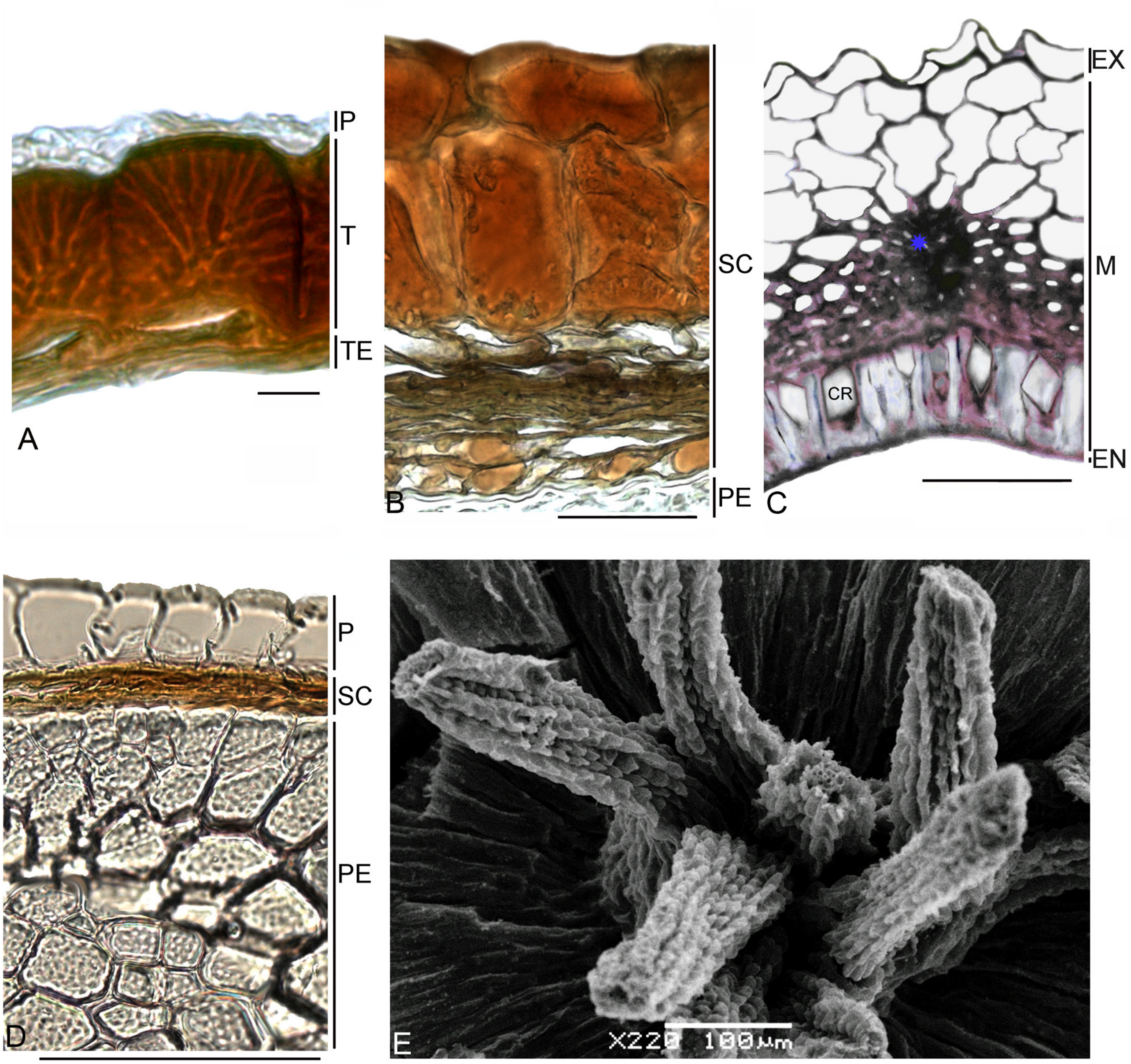

Fig 5. Carpology of the family Amaranthaceae s.str. (A-D) Transverse sections. (A) *Polycnemum arvense*. (B) *Bosea yervamora*. (C) *Pleuropterantha revoilii*, asterisk marks vascular bundle, (D) *Achyranthes bidentata*. (E) Basally located free funiculi of *Celosia trigyna*. *Abbreviations*: CR—crystal, EN—endocarp, EX—exocarp, M—mesocarp, P—pericarp, PE—perisperm, SC—seed coat, T—testa, TE—tegmen. Scale bars = 100 μm (B-E), 10 μm (A).

doi:10.1371/journal.pone.0117974.g005

U-shaped walls and finely divided content or several small prismatic crystals, and easily visible inner epidermis). Rarely, the U-shaped cells are substituted by cells with completely sclerified walls and several rhombic crystalls (*Mechowia*), or the pericarp is homocellular (Fig 5D). The seed coat is mostly thin, and the thickness of the testa is either twice that of the tegmen, or testa and tegmen are equally thick. The testa of *Pupalia lappacea* is unusual in being thick, black, and crustaceous.

Aervoids (*Aerva*, *Ptilotus*). Both genera investigated here correspond to achyranthoids in their pericarp structure, but in *Aerva* the seed coat is thicker (12–25 μm) with a stripe-like protoplast and stalactites in the outer cell walls.

Subfam. Gomphrenoideae (*Gomphrena*, *Blutaparon*, *Froelichia*, *Alternanthera*, *Tidestromia* and *Pseudoplantago* are studied here). The fruit and seed coat structure of nearly all these genera is similar to that of the achyranthoids and consists of two to three, or rarely many (*Froelichia gracilis*) layers. The crystalliferous layer in the pericarp is mostly represented by cells that have thickened walls with rhombic monocrystals in the cell content, or that rarely are fine crystals (*Blutaparon*). U-shaped cells with finely divided crystalliferous content are found in *Tidestromia oblongifolia*. The seed coat does not differ from that of most achyranthoids (except in *Pupalia*, see above).

Tribe Celosieae (*Celosia*, *Hermbstaedtia*, *Deeringia*, *Pleuropetalum*). This group is distinguished by dehiscent fruit containing several or many seeds inserted basally (Fig 5E). The one-seeded fruits have been noted only in some *Celosia* [118], but they are not an exception to the rule and have been occasionally found by us in *Hermbstaedtia glauca* and *Deeringia amaranthoides*. The pericarp of *Celosia*, *Hermbstaedtia* and *Deeringia* is divided into four topographic zones, as in *Bosea*. Both species of *Deeringia* investigated here (Asian *D. amaranthoides* and Madagascan *D. mirabilis*) have the same fruit and seed anatomy as other *Deeringia* members that can be used as an additional character to include the latter species within *Deeringia* as part of Celosieae-Amaranthaceae, as proposed by Applequist and Pratt [119]. The seed resembles *Amaranthus* in that it has a thick testa with stalactites in its outer cell walls.

Chenopodiaceae. Chenopodiaceae is one of the largest families within core Caryophyllales and comprises ~1600 species distributed worldwide but mostly outside tropical regions. Until now, only the leaf structure of Chenopodiaceae could be assigned to the recent molecular classification with subdivisions into several clades [102] that generally corresponds with the subfamilial system as proposed by Ulbrich [120]. The fruit and seed anatomy of the family (and their taxonomic implications) have been intensively studied [31], [44], [46], [47], [49], [50], [121]. We propose here that a different set of carpological characters is found for every subfamily, and suggest that our data are of particular importance for delimiting of each of them (S2 Table, Table 2; Fig 6A–6E.).

Chenopodiaceae-Amaranthaceae alliance: carpological similarities and differences. A close relationship between the two families was suggested after the discovery of a special form of their sieve-element plastids [122], and some similarities in their pollen morphology [123], [124]. The two families also share many carpological features. The Chenopodioideae (mostly Chenopodieae tribe) and *Amaranthus* species share the simple parenchymatous pericarp and seeds with hard testa containing stalactites, and Salsoloideae (Chenopodiaceae) resemble the majority of Amaranthaceae in pericarp anatomy [31] (inner epidermis, parenchyma, cells with crystalliferous content, and inner epidermis). In both families the complicated pericarp histology has evolved in the upper part of the fruit, while its structure in the lower half comprises homocellular thin-walled cells. The function of the crystalliferous layer is not clear, but it may impede the transmission of sunlight to the underlying tissue [125].

In contrast to Amaranthaceae s.str., the solitary prismatic crystals in the U-shaped cells of the pericarp were found within Chenopodiaceae only in *Fadenia zygophylloides*, before this was transferred to the genus *Salsola* [126]. No cells with equally thickened walls (O-shaped cells) and containing prismatic crystals have been found in the pericarp of Chenopodiaceae. Such monocrystals are especially common in the pericarp of Amaranthaceae-Gomphrenoideae, but seem to be rare in the vegetative organs of Amaranthaceae [127], or else the crystals are deposited in the form of sand [112]. This feature is also characteristic ofthe pericarp of *Pleuropterantha*, which was originally included in Chenopodiaceae ([128] sub Salsolaceae), and in the enigmatic Chinese genus *Baolia*, known from only one location near the border of Sichuan and Gansu provinces [129]. The presence of leaf stipulae and the details of its fruit

Table 2. Set of carpological characters for the subfamilies of Chenopodiaceae (see separate file).

Common features of Chenopodiaceae subfamilies					
**Chenopodioideae**	**Betoideae**	**Salsoloideae incl. Camphorosmeae**	**Corispermoideae**	**Suaedoideae**	**Salicornioideae**
Reproductive diaspores: (1) fruit covered in anthocarp (perianth), (2) genuine fruit, and (3) seed due to pericarp rupture. Ovary superior. The anthocarp not concrescent with pericarp, 1- or several-layered, thin-walled. There is tendency to form heteromorphic seed types. Seed with vertical annular embryo. Seed-coat testa much thicker than tegmen, smooth or alveolate. Outer cell wall of the testa often impregnated with vertical or oblique tannins (stalactites). Perisperm present.	Reproductive diaspores: (1) infrutescence, (2) genuine fruit, and (3) seed when the fruit dehists with a lid. Ovary semi-inferior or superior. The anthocarp segments of neighbouring flowers can be concrescent with each other. Pericarp free of anthocarp, many-layered, consists of several thin-walled cells and many-layered sclerenchyma below. Seed with horizontal annular embryo. Seed-coat testa much thicker than tegmen. No fruit/seed heteromorphism present. Outer cell wall of the testa often impregnated with tannins (stalactites). Perisperm present.	Reproductive diaspores: (1) fruit covered in anthocarp (perianth) and (2) genuine fruit. Fruit indehiscent. Ovary superior. Pericarp not adherent to perianth, multi-layered (at least in upper third of the fruit), differentiated into topographical zones: (1) outer, usually one-layered epidermis, (2) parenchyma, (3) U-shaped cells with crystalliferous content, and (4) inner epidermis. Seeds with horizontal, vertical or oblique located, coiled or horseshoe-shaped embryo. Seed coat smooth (with no papillae or mamillae), comprises two thin, transparent, (sub)equal layers. Stalactites in the cell walls absent. Perisperm present or absent.	Reproductive diaspores: genuine fruit. Ovary superior. Fruit indehistent oi dehistent in special portion, flattened. Pericarp not concrescent with hyaline perianth segments, often mamillate, papillate and/or with dendroid hairs, many-layered, consists of several thin-walled cells and many-layered sclerenchyma below. There is a tendency of increasing sclerenchyma layers towards the fruit margins underneath the wing. Heterocarpy or heterospermy absent. Seed with vertical annular embryo. Seed coat smooth (with no papillae or mamillae), comprises two thin, tannin-filled (sub) equal layers, rarely the outer seedcoat layer thicker (*Agriophyllum*). Stalactites in the cell walls absent. Perisperm present.	Reproductive diaspores: (1) fruit covered in anthocarp (perianrth), (2) genuine fruit, and (3) seed due to pericarp rupture. Ovary superior. Pericarp not concrescent with the anthocarp, 1-3-layered, thin-walled. Heterospermy is common in annual species. Seeds with vertical or horizontal embryo. Seed coat testa much thicker than tegmen, smooth. Outer cell wall of the testa often impregnated with vertical or oblique tannins (stalactites). Perisperm absent or as traces (in dark seed type).	Reproductive diaspores: (1) fruit covered in anthocarp (perianth), (2) genuine fruit, and (3) seed due to pericarp rupture.Ovary superior. Pericarp not concrescent with the anthocarp, 1-3-layered, thin-walled. Structural heterocarpy or heterospemy not known, but terminal and lateral flowers can be distinguished by morphometry. Seeds with horizontal or vertical located, annular or bent embryo. Seed-coat testa smooth, mamillate or papillate, much thicker than tegmen, or both testa and tegmen thin and equal in size. Outer cell wall of the testa (if thick) impregnated with vertical stalactites. Perisperm abundant or scanty.
**Unusual derived carpological characters**					
Diaspore infrutescence (some *Spinacia*). Pericarp with stellate hairs (*Krascheninnikovia*), can be tightly adherent to the anthocarp without any detachments (*Halimione*, *Ceratocarpus*), or multi-layered (some *Chenopodium*, *Oxybasis*), differentiated into anatomical zones (one of two heterocarpous fruit types in *Axyris*). Seed coat consists of equal tranparent layers (*Halimione*), with no stalactites in the outer testa cell wall (*Halimione*, all members of tribes Dysphanieae and Axyrideae). Testa with hair-like outgrowths (some *Blitum*). Embryo straight (many *Dysphania*). Seeds with vertical embryo, or both vertical and horizontal embryo positions present within one individual.	Perianth free from fruit (*Acroglochin*, *Aphanisma*).	Presence of sclereids in the pericarp between U-shaped cell layer and inner epidermis (Salsoleae: *Lagenantha*, *Halothamnus*); reduction of U-shaped cells (*Noaea minuta*) or presence of O-shaped cells with prismatic crystals (*Fadenia zygophylloides*). Outer epidermis or rarely inner epidermis of several layers (some *Anabasis* species).	No unusual derived characters	Pericarp and perianth adherent (at least in lower fruit part) in *Bienertia* and some *Suaeda*.	Pericarp lignified in some Australian taxa. Outer seed coat layer with hair-like outgrowths (*Salicornia*, *Sarcocornia*).

doi:10.1371/journal.pone.0117974.t002

anatomy do not confirm the previous placement of *Baolia* within the tribe Chenopodieae, as proposed by Zhu [130]. The classification of *Baolia* within subf. Polycnemoideae as a part of Chenopodiaceae [131], that is now considered to be a member of Amaranthaceae s.str. [102], [109], is also weakly supported by two unique carpological characters: pericarp alveolation resulting from the rupturing of the thin outer cell wall of the outer pericarp layer, and the presence of monocrystals in the O-shaped cells in the innermost mesocarp layer.

A second difference between the two families concerns the diverse spatial embryo position in the seeds. The emergence of the horizontally oriented embryo in many Chenopodiaceae groups is not found in the Amaranthaceae or in almost all the other one-seeded members of core Caryophyllales. Some groups of Chenopodiaceae can also be distinguished by spatial heterospermy in the partial inflorescences, which likely evolved mixed horizontal and vertical embryo positions within one individual plant [49], [132].

'Globular Inclusion' clade.. Sarcobataceae. This monotypic family (1–2 shrubby species in North America), which was established by Behnke [133] on the basis of the distinctive sieve-element plastids, is well supported by molecular results [3]. However, some recent authors [37], [134], [135] still accept its placement within the Chenopodiaceae [120], [136]. We postulate here that the development of a radial wing from the middle portion of the pericarp of *Sarcobatus* (Fig 6F) is a unique case in the core Caryophyllales, in contrast with the "winged diaspores" in Chenopodiaceae, which (when present) are formed mostly from the perianth, or rarely the marginal parts of the fruit. The pericarp anatomy of *Sarcobatus* sets it apart from all Chenopodiaceae studied to date [31], [44], [46], [49], [137], [138]. Except for the wing area, the pericarp is ribbed, flattened in the lower half (up to the wing), and unequally thick, from 100–125 μm in the ribs, to 50–60 μm between them, and it is differentiated into several topographic zones (Fig 7A): (1) a unicellular epidermis possessing scattered T-shaped, stellate and simple trichomes; (2) parenchyma cells that contain druses; (3) mechanical tissue of 1–2 layers arranged parallel to the fruit axis; (4) an inner epidermis. Vascular bundles are present in the ribs. The cells of the inner epidermis in the rib zone sometimes bear short cylindrical papillae. The wing consists of parenchyma and fibers with the cells from 1 mm long. The two-layered seed coat does not adhere but tightly adjoins the pericarp. Its cells are thin-walled, equal in size, or (between the ribs) the outer layer consists of spongy cells. The seed embryo is vertical, spirally coiled in two turns; the perisperm is scarious, one- to two-layered, and is located peripherally near the seed coat.

Although the family belongs to the so-called 'Raphide clade' [4], only druses are found in the pericarp parenchyma.

Nyctaginaceae. The Nyctaginaceae is mostly an American family (~400 sp.) with a few members found in Africa, Asia and Australia [139], [140]. A diagnostic character of Nyctaginaceae is the persistent lower part of the perianth that usually appears to be accrescent, fully enveloping the fruit and as a rule containing mucilage [141–143]. In contrast to the taxonomic importance in the family of the anthocarp structure [98], [144–146], the pericarp and seed coat seem to be less diversified. The pericarp is dry, smooth, rarely ribbed (*Guapira graciliflora*) and not papillate, but can have stellate trichomes on its surface (and other organs), a feature that is peculiar to the Leucastereae [147]. The pericarp is one- to two-(three)-layered, rarely four- to five-layered and not divided into different topographic zones but tightly adjoining the seed coat. According to the embryological studies, the one-layered, thin pericarp in Nyctaginaceae results from the obliteration of all of the innermost layers [148], [149]. However, some genera are characterized by having a pericarp that consists of several or many layers that can be rather transparent, although sometimes they may be brownish due to the presence of tannin-like substances. The robust 5-12-layered parenchymatous pericarp in the ripe fruits is found in *Andradea* as part of tribe Leucastereae and in *Bougainvillea* (Bougainvilleeae). Although the pericarp

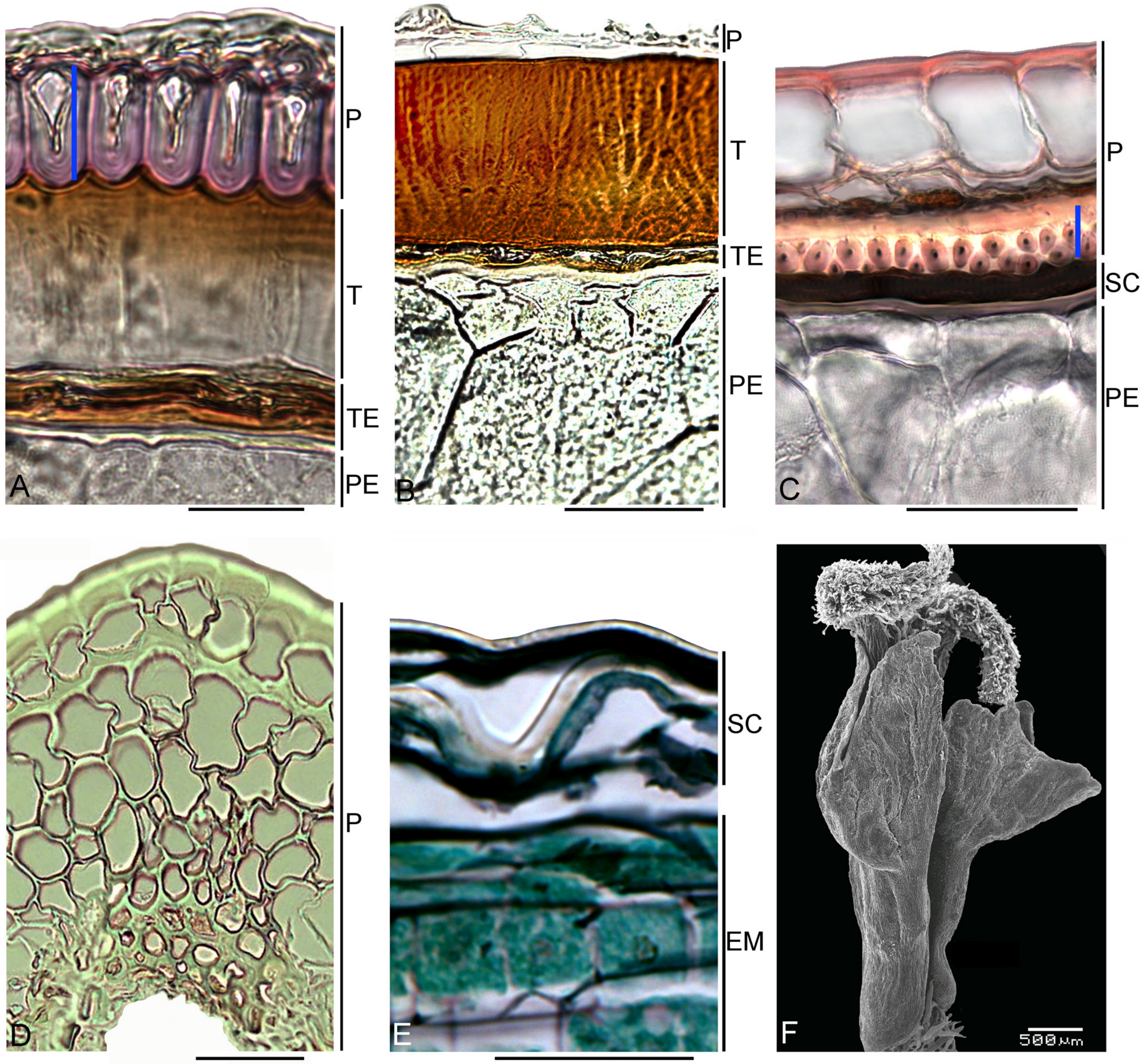

Fig 6. Carpology of the families Chenopodiaceae (A-E) and Sarcobataceae (F). (A-E). Transverse sections. (A) Pericarp and seed coat of one of the dimorphic fruits of *Axyris amaranthoides* (Chenopodioideae-Axyrideae), vertical blue line indicates sclereid layer; cell cavities increase towards outside; crystals not present.(B) Fruit and seed of *Chenopodium giganteum* (Chenopodioideae-Chenopodieae). (C) Fruit and seed of *Coryspermum sibiricum* (Corispermoideae), vertical blue line indicates layer of sclereids. (D) Pericarp in the upper part of the fruit of *Anabasis cretacea* (Salsoloideae). (E) Seed coat of *Anabasis cretacea*. (F) SEM image of the young fruit of *Sarcobatus baileyi* forming radial wing. *Abbreviations*: EM—embryo, EN—endocarp, EX—exocarp, M—mesocarp, P—pericarp, PE—perisperm, SC—seed coat, T—testa, TE—tegmen. Bars = 10 μm (A), 50 μm (B-E), 500 μm (F).

doi:10.1371/journal.pone.0117974.g006

of *Andradea* possesses vascular bundles with bar-thickened walls that are not found in other genera, it is exceptionally brittle. The alveolate fruit wall of *Cryptocarpus* and *Salpianthus* bears three to six layers of dead, tannin-filled cells with inclusions of raphides located subepidermally (*Cryptocarpus*) or as the innermost pericarp layer (*Salpianthus*). A similar pericarp structure is observed in *Boldoa purpurascens*, but the fruit wall is thinner, and the raphides are visible with

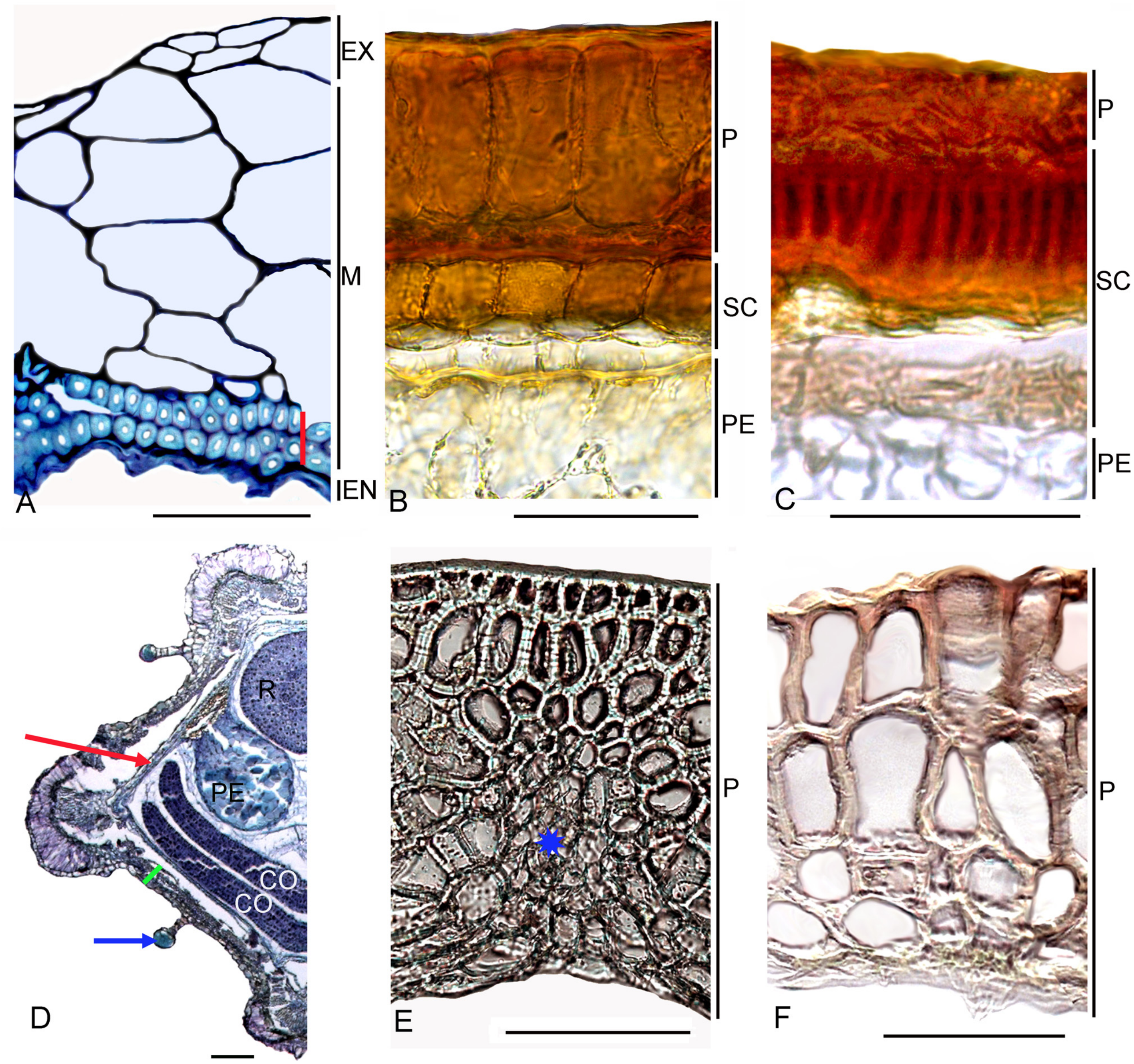

Fig 7. Carpology of the families Sarcobataceae (A), Nyctaginaceae (B-D), Basellaceae (E) and Didiereaceae (F). Transverse sections. (A) *Sarcobatus baileyi*, vertical red line indicates sclereid layers. (B) *Colignonia scandens*. (C) *Cryptocarpus pyriformis*; (D) *Boerhavia diffusa*, fruit half with anthocarp. Blue arrow indicates the glandular hairs on the anthocarp surface; red arrow indicates the thin pericarp and seed coat, green line indicates anthocarp. (E) *Tournonia hookeriana*, asterisk marks vascular bundle. (F) *Calyptrotheca taitensis*. *Abbreviations*: CO—cotyledon, EN—endocarp, EX—exocarp, M—mesocarp, P—pericarp, PE—perisperm, R—radicle, SC—seed coat. Bars = 100 μm (A, D-F), 50 μm (B-C).

doi:10.1371/journal.pone.0117974.g007

the naked eye as white striae. A multi-layered pericarp was found in one of the samples of *Pisonia aculeata*.

The seed coat consists of two to four membranous layers formed by both integuments (testa and tegmen), but they often appear to be equally thick, or the testa is insignificantly thicker than the inner layer(s). In the tribes Leucastereae, Colignonieae and Boldoeae the seed coat is described as crustaceous [98]. These groups are distinguished by having a thick seed coat, but their structure is not equivalent. Colignonieae and Leucastereae have large testa cells with

well-developed intermediate layers, but there is no decrease of the protoplast or impregnation of the cell walls with the stalactites (Fig 7B). The seed coat is brittle in Leucastereae. *Boldoa purpurascens* (Boldoeae), *Cryptocarpus pyriformis* and *Salpianthus aequalis* have black seeds with a robust testa possessing stalactites in its outer cell walls which influence the compression of the cell content (Fig 7C). The stalactites in the testa are not mentioned in other Nyctaginaceae tribes. The majority of representatives in the other tribes clearly have a thinner seed coat with tangentially elongated testa and tegmen cells (Fig 7D). The embryo is vertical and usually curved with perisperm often present. Only in the tribe Pisonieae in its recent circumscription [150] do the seeds have a straight embryo that fills the seed, and in most cases the perisperm is lost. A slightly bent and small embryo is found in *Colignonia scandens*. We observed raphides in the embryonic cotyledons of *Commicarpus* species.

The family is distinguished by three peculiarities in their fruit/seed anatomy within all one-seeded core Caryophyllales: (1) the presence of scattered raphides in the pericarp, seed coat, and even the embryo (e.g. *Commicarpus*), mentioned by Wilson [151] for the testa cells of some *Abronia*, where they were called ‘aleurone grains’. These crystalliferous inclusions are also present in vegetative organs [127], [152–156]; (2) in many of the Nyctaginaceae the pericarp is one-layered and very thin (a few mm thick) tightly adjoining the seed coat; (3) the perisperm (if present) is two-lobed or consists of two separated parts; it is entire only in *Boldoa*, *Salpianthus* and *Cryptocarpus*.

Basellaceae. This is a small family of four genera (*Basella*, *Ullucus*, *Anredera*, *Tournonia*) with diversity in the Tropics, predominantly of the New World. Until now, the pericarp of *Basella* has sometimes been considered to be fleshy [23], [157]. We agree with investigations by Franz [158] and Lacroix [159], and with the exact morphological descriptions of the Madagascan endemics of *Basella* [160], who noted that the consistency and colour of the diaspore is mostly provided by water-filled floral structures (subtending bracts and tepals) that tightly envelop the fruit, while the fruit itself is hard in all members of the family. The exocarp of *Basella*, represented by a sclerified layer, can also be slightly pink [18] due to pigmentation of the cell contents. In *Basella alba* and *B. rubra*, the exocarp cells are radially elongated, but in *B. paniculata* some parts of the fruit may contain two sclerified layers (exocarp and outermost mesocarp layer) with no cell elongation. The underlying 2–4 mesocarp layers can consist of unlignified parenchyma, but in some fruits these layers are represented only by fibers that are rounded in cross-section. The seed coat tightly adjoins the pericarp and comprises several layers with a prominent development of the testa [23].

In other genera these elongated exocarp cells are not found. The outer sclerenchymatous layer is present in both *Anredera brachystachya* and *A. scandens* with a parenchymatous layer beneath. However, in the basal part of the fruit of both mentioned *Anredera* species investigated and especially in its upper portion (near stylodia), the pericarp layers increase to five to seven layers due to the emergence of parenchyma covering the sclerenchymatous layer from above, while such parenchyma is lacking in the middle or lower parts of the fruit. Within the core Caryophyllales, this structure seems to be a unique case in which the epidermal layer transitions from parenchyma to sclerenchyma depending on the fruit topography. *Anredera cordifolia*, formerly placed within the genus *Boussingaultia* (*B. gracilis*) and now recognized as a member of *Anredera* [161], possesses parenchymatous pericarp layers. The same topography is found in *Ullucus tuberosus*, although its pericarp appears to be alveolate. *Tournonia hookeriana* has a many-layered pericarp made up entirely of sclerenchyma (Fig 7E).

The most recognizable carpological characters of Basellaceae are: (1) globose fruits, (2) significant tanniniferous impregnation of the outer cell wall of the testa in contrast to other seed-coat cell walls (the inner cell wall of the tegmen is also impregnated with tannins in *Basella*), and (3) embryos with a tendency to be spirally twisted [162].

Didiereaceae (*Alluaudia*, *Calyptrotheca*, *Ceraria*, *Decarya*, *Didierea* have been studied).

Its new wider circumscription includes the Madagascan members *Didierea*, *Alluaudia*, and *Decarya*, and the South and East African *Ceraria*, *Calyptrotheca* and *Portulacaria* [163], [164]. Almost all taxa have one-seeded fruits, including *Calyptrotheca* that have unusual splitting of the capsule from the base upwards [165], although we have observed one to three normally developed seeds in *Calyptrotheca somalensis*. Some of the genera (*Didierea*, *Decarya*) are distinguished by trigonous fruit outlines that resemble those of some Polygonaceae, and *Ceraria* possesses flattened fruits that sometimes have large simple trichomes (*C. namaquensis*). The general pericarp topography (Fig 7F) is found to be the same in almost all genera investigated (except *Ceraria*): sclerenchyma as outer layer(s), and unlignified, sometimes hardly noticeable or crushed cells beneath with prominently expressed mesocarp layer(s). A one-layered sclerenchyma (exocarp) is found in *Didierea*, *Alluaudia* and *Decarya* (subfam. Didiereiodeae), whereas *Calyptrotheca* (Calyptrothecoideae) possesses several lignified layers substituted below by tangentially elongated and sometimes crushed parenchyma. Two investigated *Ceraria* species are distinct in their pericarp anatomy: *C. namaquensis* has a multi-layered pericarp consisting of 7–10 layers of parenchyma and 1–2 sclerenchyma layers beneath, with large air cavities in the marginal parts supported by a sclerenchymatous sheath, and *C. longipedunculata* possesses a 3-4-layered, thin fruit wall with an indistinct exocarp with scattered glandular trichomes as well as an endocarp, and a prominent intermediate cell layer filled with tannins.

The seed is supplied with an aril (except *Ceraria*), and there is no perisperm; the embryo is in a vertical position. The seed-coat testa can be robust, especially in *Calyptrotheca*, sometimes with a prominent intermediate layer between the testa and tegmen. The tegmen is always easily visible with the cell having remarkable bar-thickened walls.

Carpology of the Phytolaccaceae cohort with predominantly one-seeded groups. Currently this large family is considered to be polyphyletic. It is actively being re-evaluated and has been placed in several different positions in the trees of the core Caryophyllales [2], [3], [6], [166–168]. The placements of some members are unstable, e.g. *Monococcus* [168], but some cohort members (e.g., Rivinaceae and Petiveriaceae) seem to be closely related based on molecular results [5] and share a similar wood anatomy [169].

Lophiocarpaceae. Both genera *Lophiocarpus* (~12 spp., South Africa) and *Corbichonia* (2 spp. in South and Eastern Africa, one of them, *C. decumbens*, has radiated into the Arabian floristic province) together form a separate lineage that should be excluded from Phytolaccaceae s.str. and Molluginaceae [3]. They were later united in their own family Lophiocarpaceae [170]. They are, however, not closely related to each other embryologically [171–173] or in flower morphology [42], [172], fruit type (many-seeded capsule in *Corbichonia* vs.one-seeded indehiscent fruit in *Lophiocarpus*), seed-coat testa that can be alveolate in *Lophiocarpus* or with short papilla-like outgrowths in *Corbichonia* [174], or the presence of a seed aril (only *Corbichonia*). We provide additional differences in the fine carpology of both genera (Table 3; Fig 8A–8C). Due to

Table 3. Additional carpological differences between *Lophiocarpus* and *Corbichonia*.

Characters	*Lophiocarpus*	*Corbichonia*
Pericarp	verrucose, rarely ribbed, completely or partially with unlignified cells, layers 4 to 10 (in inflated parts or ribs)	smooth, outer cells with lignified walls, the innermost cells without lignification; layers 4 to 8
Seedcoat testa	alveolate, in most species 20–50 μm (ca. 80 μm in *L. latifolius*), with almost straight stalactites	sinus-like, triangular in cross-sections, ca 50 μm, warty in upper part, stalactites obliquely oriented

doi:10.1371/journal.pone.0117974.t003

important differences in the reproductive characters, the placement of *Corbichonia* into Lophiocarpaceae needs further investigations.

Rivinoideae clade (*Rivina*, *Trichostigma*, *Hilleria*, *Petiveria*, *Gallesia* and *Seguieria* are investigated here). Carpologically, the representatives are clearly split into two groups. The first comprises *Rivina*, *Trichostigma*, and *Hilleria* with more or less fleshy fruits, a homocellular pericarp and a hard seed-coat testa (Rivinaceae s.str.), sometimes with acicular outgrowths of the testa cells (some *Rivina*: Fig 8D). The second group (Fig 8E and 8F) unites the representatives with dry fruits and a pericarp differentiated into several topographic zones (outer

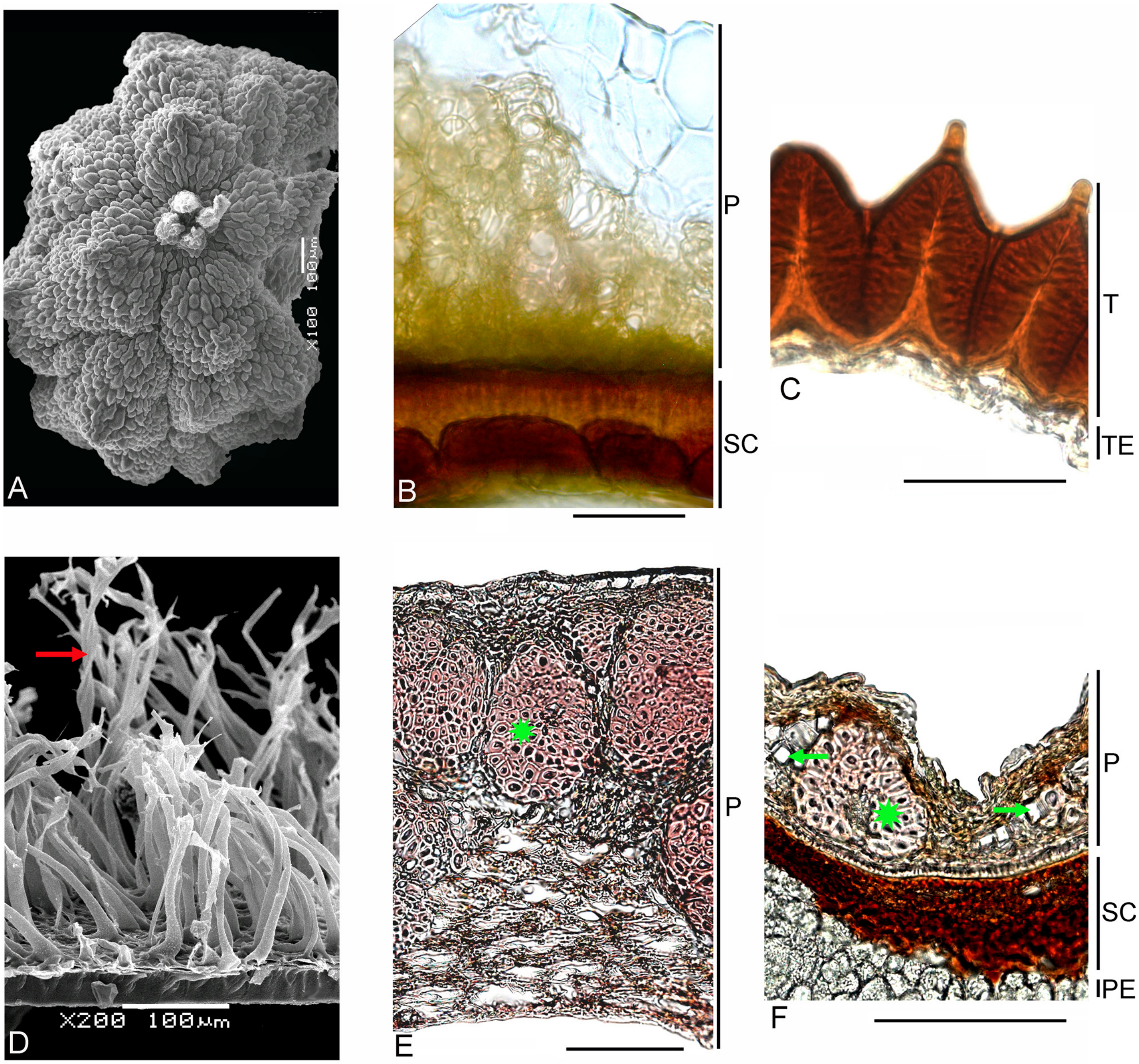

Fig 8. Carpology of the families Lophiocarpaceae (A-C), Rivinaceae (D-E) and Petiveriaceae (F). (B-F) Transverse sections. (A) SEM of the fruit (top view) of *Lophiocarpus tenuissimus*. (B) *Lophiocarpus burchellii*. (C) *Corbichonia decumbens*. (D) SEM of the seedcoat of *Rivina brasiliensis*, red arrow indicates trichomes. (E) *Seguieria aculeata*, asterisk marks one of the nests of sclereids. (F) *Petiveria alliacea*, green arrows indicate crystals and asterisk marks one of the nests of sclereids.Bars = 100 μm (A, D-F), 50 μm (B-C). *Abbreviations*: P—pericarp, PE—perisperm, SC—seed coat, T—testa, TE—tegmen.

doi:10.1371/journal.pone.0117974.g008

epiderm, underlying parenchymatous layers, thick sclerenchymatous sheath, inner parenchyma of one to three cell layers), and a thin seed coat with no important differences in the thickness of the layers (*Petiveria*, *Seguieria*, *Gallesia*). However, *Petiveria* (Petiveriaceae s.str.) and both *Seguieria* and *Gallesia* (Seguieriaceae s.str.) disagree in several main characters. The fruits of *Petiveria* terminate in (mostly) four reflexed aristae that probably facilitate epizoochoric dispersal. Both members of Seguieriaceae are characterized by the flattened wing-like stylodium of the fruit that enables anemochorous dissemination. The perisperm is easily visible only in *Petiveria*, and is almost absent in both *Seguieria* and *Gallesia*. The embryo is straight with plicate cotyledons (*Petiveria*), or annular in the Seguieriaceae.

Stegnospermataceae. A monotypic family of several shrubby species distributed in the Tropics of the New World. In contrast to Phytolaccaceae s.str., some characters are distinctive, for example, a diffuse axial parenchyma in the stem [175], sieve-element plastids [122], flower morphology [176], embryology [177], a two-loculed capsule as fruit type with 1–2 seeds in each locule, and the presence of a seed aril [21]. The valves of the capsule in *Stegnosperma cubense* are leathery, from 300 to 600 μm, with subdivisions into three topographic zones: (1) one-layered, radially elongated, sclerenchymatous parenchyma, (2) several-layered, thin-walled parenchymatous cells, and (3) four to eight layers of tangentially elongated fibers. The seeds are typical for the core Caryophyllales in having a large testa (100 μm) with an easily visible cell content and a thin striate tegmen layers with bar-thickening of the radial walls. Both molecular and carpological data suggest that Stegnospermataceae cannot be considered as a family with primitive individual characters within core Caryophyllales as proposed by Hofmann [178].

Bar-thickening of the endotegmen cells—one additional parameter in the description of the core Caryophyllales

From the nine characters that unite many of the core Caryophyllales, three traits belong to the seed structure (curved ovules, seed coat composed of exotesta and endotegmen, and well developed perisperm [23]). Another character that can be added to the existing description of this group is the presence of bar-thickening in the tegmen cell walls. The first comprehensive data about this peculiarity were provided by Kowal [179], [180] for *Chenopodium*, *Atriplex* (Chenopodiaceae), and *Amaranthus* (Amaranthaceae). Indeed, many members of the Chenopodiaceae-Amaranthaceae alliance are found to have bar-thickened tegmen (S2 Table): Chenopodiaceae-Chenopodioideae (*Chenopodium* s.str., *Atriplex*, *Lipandra*, *Oxybasis*, *Blitum*), Chenopodiaceae-Suaedoideae (*Suaeda*) and many genera of Amaranthaceae s.str. of different taxonomic position. Besides Chenopodiaceae and Amaranthaceae, this feature was also discovered in the present study in the tegmen cell walls of Achatocarpaceae, Rivinaceae s.str. (Fig 9), Stegnospermataceae, Seguieriaceae, Petiveriaceae, Microteaceae, Lophiocarpaceae, Macarthuriaceae, gen. *Adenogramma*, only a part of Nyctaginaceae and Caryophyllaceae. The Rhabdodendraceae is set apart from all the members of the alliance by having all (three to four) seed coat layers with bar-thickened walls. Among many-seeded fruits, such endotegmen cell walls are reported in the seeds of Aizoaceae, including Tetragoniaceae, Molluginaceae, Portulacaceae, Didiereaceae, Basellaceae, Cactaceae [23], and Anacampserotaceae [181] and were found in the present study in *Macarthuria* sp. (Macarthuriaceae), *Gisekia pharnaceoides* (Gisekiaceae) and *Limeum obovatum* (Limeaceae).

Does the origin of one-seeded fruits based on molecular phylogeny coincide with the embryological data?

The discussion about the origin of the one-seeded nuts from many-seeded capsules is well-known but only in the Phytolaccaceae s.l. and Caryophyllaceae. In previous investigations,

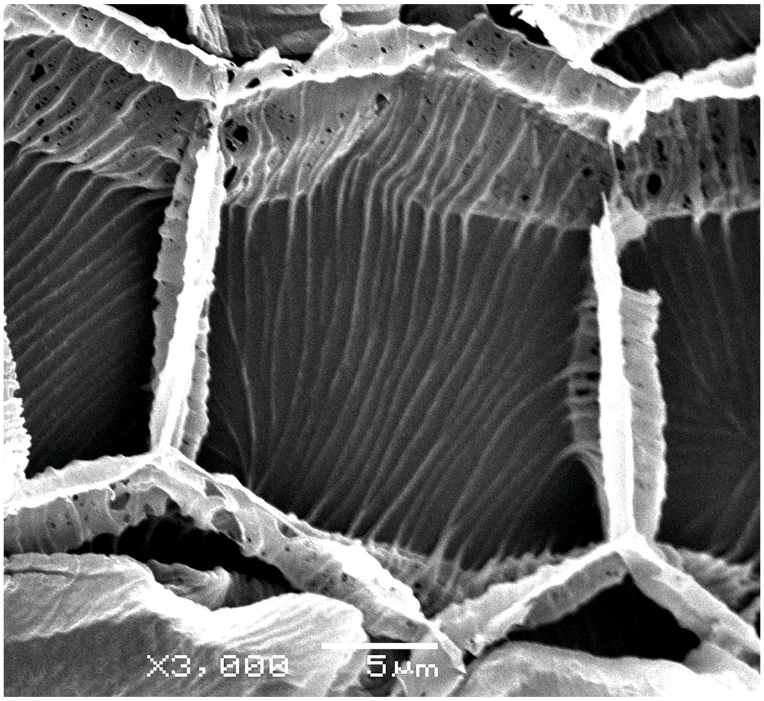

Fig 9. Endotegmen cell walls of *Rivina humilis* (SEM). Bar: 5 μm.

doi:10.1371/journal.pone.0117974.g009

Phytolacaceae was considered in a broader sense to include the one-seeded Rivinaceae, Seguieriaceae and Petiveriaceae, and it was suggested that the origin of the one-seeded fruits in Phytolaccaceae s.l. was connected with a reduction of the carpels [182–186]. According to the molecular phylogeny, the origin of the one-seeded fruits in Rivinaceae and its relatives cannot be directly assigned on the basis of Phytolaccaceae s.str., but we conclude that the multi-seeded fruit type is an ancestral state in the Phytolaccaceae s.str. clade and relatives, and that it has been converted into the one-seeded state in the clades Agdestiaceae + Sarcobataceae / Nyctaginaceae + Seguieriaceae + Rivinaceae + Petiveriaceae. This conclusion supports the results of other investigations [14], [187].

Recent statement concerning the ancestral characters of one-seeded fruits in Caryophyllaceae + Corrigiolaceae [43] are still contradicted by embryological data that mostly suggest the derivation of the one-seeded fruits from syncarpous capsules [40], [188], [189], with reduction of the central fruit column bearing the seeds with their placentas and septa. In the light of the molecular phylogeny, the increase of seed number in the fruit might be connected with the following basic transitions: (1) one basal ovule in the fruit as an ancestral character state (as in *Corrigiola*); (2) an increase in ovule numbers with maintenance of free funiculi. This is a common trend traced in many groups including *Telephium* (Corrigiolaceae) to several higher clades of Caryophyllaceae s.str. (Sperguleae, Sagineae, Arenarieae, Alsineae, a part of Polycarpaeae and Sclerantheae); (3) emergence of the central fruit column formed from the concrescent lower parts of the funiculi that can be extended to the fruit apex in the clades Caryophylleae and Sileneae. In these possible changes in fruit structure, only the evolutionary emergence of the septa between the central column and fruit margins whose fusion is postgenital [190] remains unclear and should be clarified by further detailed investigations. Thus the syncarpy-to-lysicarpy paradigm in the family needs to be reinterpreted with respect to most of the family.

Reconstruction of the carpological characters

For the reconstructions of the seed and fruit ancestral states we have chosen the most important characters concerned with the changes in the fruit and seed structure. All (one-, many-seeded, or associated) fruit types have been taken into account.

Pericarp succulence (Fig 10)—All the major clades, including the Polygonids, possess a dry pericarp. Fleshy and (often) coloured pericarp fruits originated independently in the AAC clade (*Achatocarpaceae*), some representatives of *Amaranthaceae* (*Bosea*, *Pleuropetalum* and *Deeringia*) and Chenopodiaceae (*Anabasis*). The mixed pericarp resulting from heterocarpy within one individual originated at least twice in *Chenopodiaceae—Holmbergia tweedi* and *Chenopodium nutans* and relatives (*Chenopodium* sect. *Rhagodia* and *Einadia*), and is connected with changes in the consistency of dry fruit types. In the 'Globular Inclusion' clade,

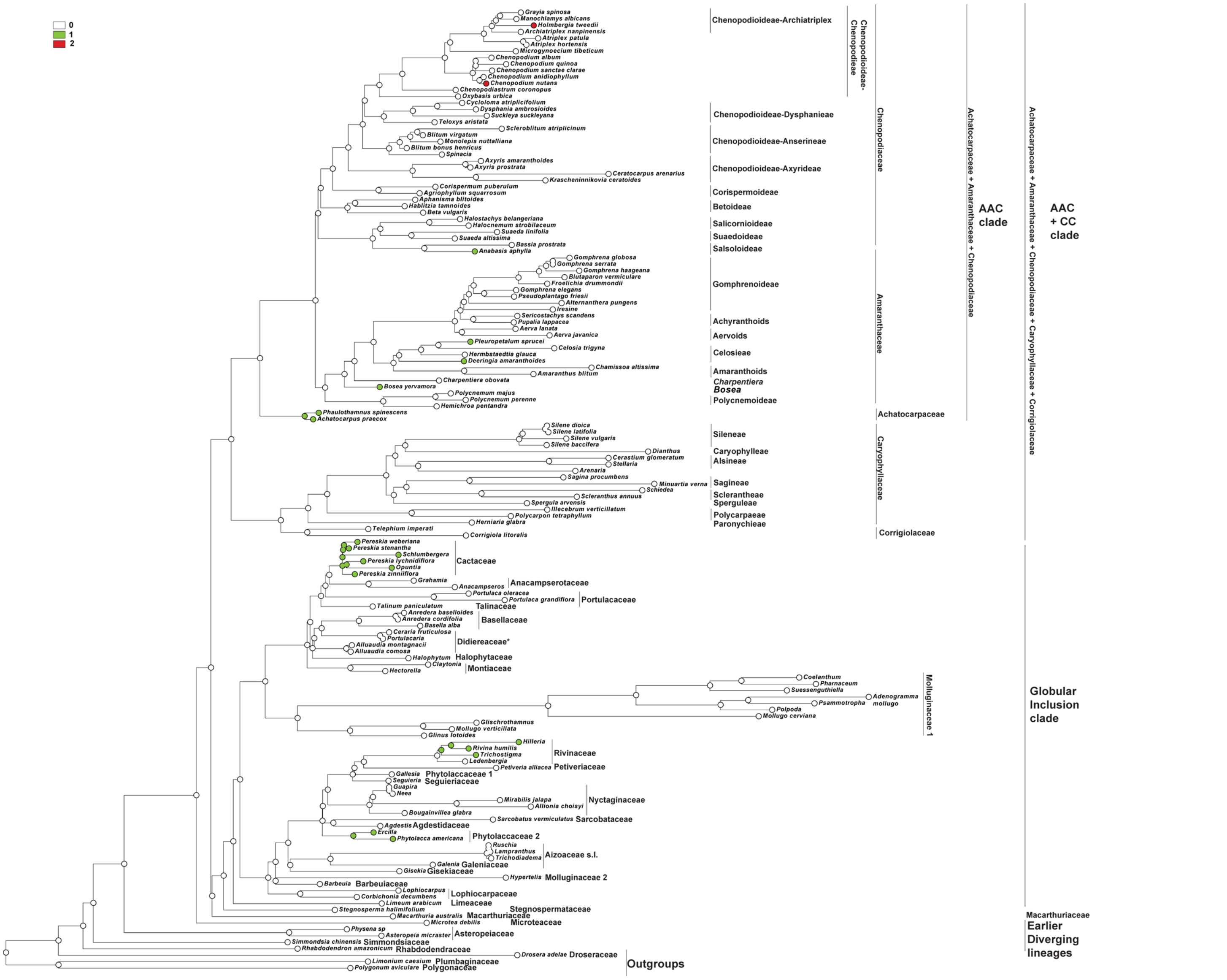

Fig 10. MP reconstruction of the evolutionary history of pericarp succulence of combined plastid dataset. Character states: 0—dry, 1—tendency to be fleshy and coloured (or rarely transparent); 2—both dry and fleshy fruits in one individual. Morphological characters treated as unordered.

doi:10.1371/journal.pone.0117974.g010

fleshy fruits have originated several times at least in Phytollacaceae (*Ercilla*, *Phytollaca*), Rivinaceae (*Hilleria*, *Rivina*, *Trichostigma*), and also in the large family Cactaceae. There are no reversions to the dry fruit types.

Pericarp layers (Fig 11). Pericarps consisting of more than three layers are the ancestral character state in almost all the clades, and all deepest groups in the core Caryophyllales (Simmondsiaceae, Physenaceae, Asteropeaceae, Microteaceae), as well as the majority of the Caryophyllaceae, and the Amaranthaceae s.str. are characterized by a multi-layered pericarp. The simplification of the pericarp to one to three layers evolved independently in both the AAC+CC and the 'Globular Inclusion' clades. In the latter clade, this trend is anchored in the large family Nyctaginaceae (the clades after *Bougainvillea*: *Neea*, *Guapira*, *Allionia*, *Mirabilis*). Reduction of the number of layers is also traced in Amaranthaceae (Polycnemoideae) and *Herniaria*

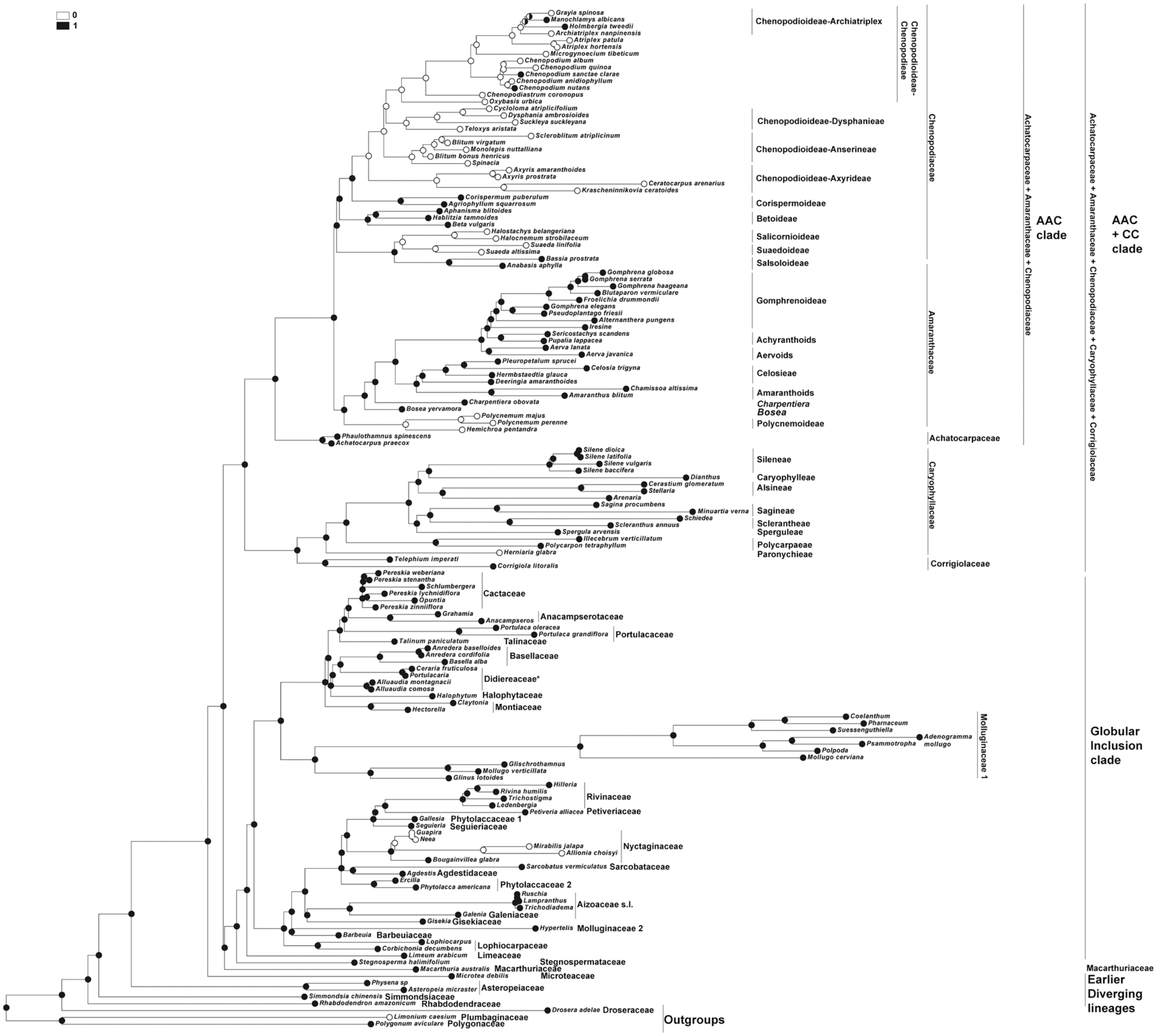

Fig 11. MP reconstruction of the evolutionary history of pericarp layers of combined plastid dataset. Character states: 0–1(2–3) layers, 1—more than 3 layers. Morphological characters treated as unordered.

doi:10.1371/journal.pone.0117974.g011

(Caryophyllaceae). In Chenopodiaceae, the multi-layered pericarp is the ancestral state in Salsoloideae, including Camphorosmeae (*Anabasis*, *Bassia*), Betoideae and Corispermoideae. However, a reduction in number of pericarp layers evolved independently in Suaedoideae + Salicornioideae and almost all Chenopodioideae, with further reversion to an increase in the pericarp layers in *Chenopodium* sect. *Rhagodia*, genera *Holmbergia* and *Manochlamys*.

Pericarp topography (Fig 12). The reconstruction of the pericarp topology suggests that the ancestral state for the core Caryophyllales + polygonids, as well as for 'Earlier Diverging' lineages, both the AAC+CC clade and the 'Globular Inclusion' clade, is a pericarp divided into two zones: sclerenchyma as outer layers with thin-walled parenchyma below. Such a fruit-wall arrangement exists in the *Physenaceae* and *Asteropeiaceae* (as well as in some polygonids) and it is typical in Corrigiolaceae and many members of *Caryophyllaceae*. However, Rhabdodendraceae and Simmondsiaceae evolved the distinct pericarp topography that is typical for drupes (*Rhabdodendron*) or similar to other deepest Caryophyllales but with complicated structure (*Simmondsia*).

In the AAC+CC clade, the pericarp topography has radically changed from an initial sclerenchymatous-parenchymatous pericarp into two different types. The least frequent change is to parenchyma as the outermost layer(s) with sclerenchyma beneath, and is traced in *Scleranthus* (Caryophyllaceae-Sclerantheae). Simplification of the fruit-wall structure to one or several homocellular layers occurred independently in several clades; it is especially common in some Paronychieae and Polycarpaeae, and is found as rare exceptions in Plurcaryophyllaceae (e.g. *Stellaria monosperma*). The conversion of the pericarp topography into several different zones evolved in the AAC clade, with further changes in pericarp structure expressed in its simplification in Suaedoideae + Salicornioideae, and conversion to parenchyma as the outermost layers with sclerenchyma below in Betoideae + Corispermoideae. The ancestral character state in subfamily Chenopodioideae might be characterized by both homocellular and parenchymatous-sclerenchymatous pericarp, but many lineages of Chenopodioideae (Chenopodieae and a part of Axyrideae: *Krascheninnikovia* + *Ceratocarpus*) have independently evolved the homocellular pericarp.

In the 'Globular Inclusion' clade, the ancestral pericarp zonation remains in many groups in the"Portulaceous alliance" (Didieriaceae, Talinaceae, Portulacaceae), while the members of the 'Raphide clade' evolved a complicated (*Petiveria*, *Sarcobatus*, *Gallesia* and *Seguieria*) or simplified (*Nyctaginaceae*, *Phytolaccaceae* s.str., *Rivinaceae*) pericarp structure. In some families with many-seeded fruits (Cactaceae, Aizoaceae), the pericarp topography has not yet been studied.

Embryo orientation (Fig 13). The vertical embryo position in the one-seeded fruits is an ancestral character state in all the large clades of the core Caryophyllales. Such a position was also retained when the multi-seeded fruit types (first originated from one-seeded fruit types) were decreased to single-seeded nuts in 'Globular Inclusion' clade and Caryophyllaceae. The changeto the horizontal position occurred independently in some *Chenopodiaceae* (*Salsoloideae*, *Betoideae*, *Chenopodioideae*), with later reversion to the vertical position in *Grayia*, *Manochlamis*, *Holmbergia*, *Archiatriplex*, *Atriplex*, *Microgynoecium*, and *Suckleya* (all Chenopodioideae). The mixed-embryo state within a single individual plant may have originated in two ways: from a horizontal position in some *Dysphania*, and from a vertical position in *Atriplex* and *Blitum*.

Conclusions

The one-seeded fruits in the core Caryophyllales are inferred to be an ancestral character state in the 'Earlier Diverging' lineages (Rhabdodendraceae, Simmondsiaceae, Asteropeiaceae, Physenaceae) and AAC+CC clade. The origin of the one-seeded fruits is a reversion from

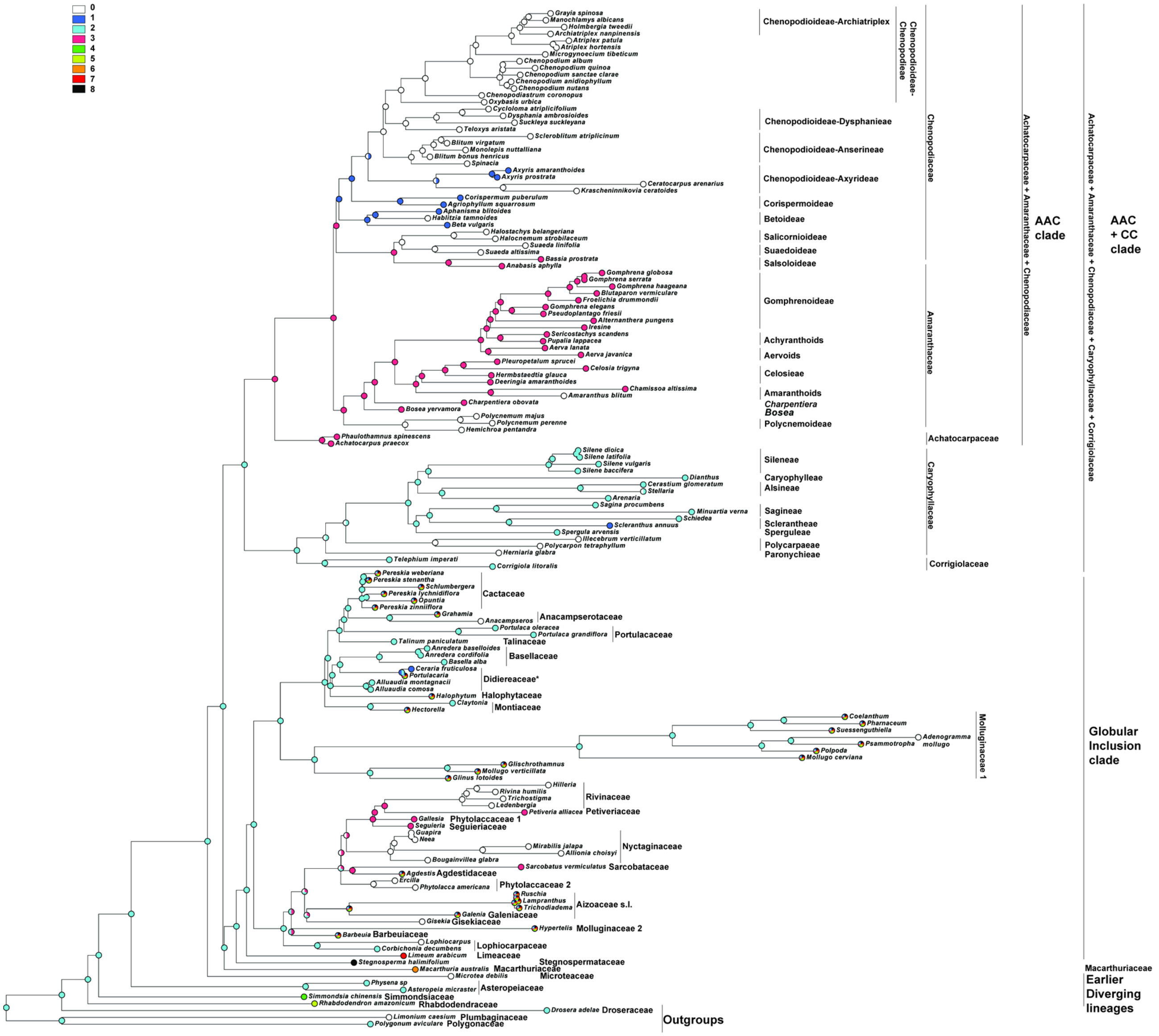

Fig 12. MP reconstruction of the evolutionary history of pericarp topography of combined plastid dataset. Character states: 0—no drastic differences in the consistency of all layers (cells parenchymatous, non-lignified or rarely sclerenchymatous), or only one layer is present; 1—differentiated into parenchyma as outermost layer(s) and sclerenchyma beneath (at least in some fruits, if heterocarpic); 2—differentiated into sclerenchyma (or sclerenchymatous parenchyma) as outermost layer(s) and thin-walled parenchyma; 3—differentiated into: (a) outer parenchymatous epidermis; (b)—thin-walled parenchyma (but sometimes reduced); (c)—sclerenchyma present as O-shaped cells (with equally thickened walls) or U-shaped cells (unequally thickened walls) that often contain crystals in the protoplast; (d)—inner epidermis (sometimes obliterated); 4—differentiated into: (a) outer sclereid layers; (b) thin-walled parenchyma intermixed with brachysclereids; (c) crumpled parenchyma; (d) inner epidermis; 5—differentiated into: (a) 1 or several layers with thick walls; (b) thin-walled parenchyma; (c) brachysclereids with walls filled with tannins (fruit is a typical drupe); 6—divided into (a) thick outer epidermis, (b) thin-walled parenchyma, and (c) thick inner epidermis; 7—divided into (a) parenchyma as outermost layer(s), b—sclerenchymatous layer(s), c—parenchyma layer(s); 8—divided into (a) sclerenchymatous parenchyma, (b) thin-walled parenchyma, (c) multilayered fibers. The position 9 (see text) is not shown on the tree due to lacking of the samplings of both *Anredera brachystachya* and A. *scandens*. Morphological characters treated as unordered. Ambiguities recoded as polymorphic states.

doi:10.1371/journal.pone.0117974.g012

multi-seeded fruits in the 'Globular Inclusion' clade. The dry and multi-layered pericarp, topographically divided into sclerenchyma as outermost layer(s) and parenchyma below, is an initial state in the core Caryophyllales, with a tendency to reduction in the number of layers in many clades, especially in large families such as Nyctaginaceae and Chenopodiaceae. The

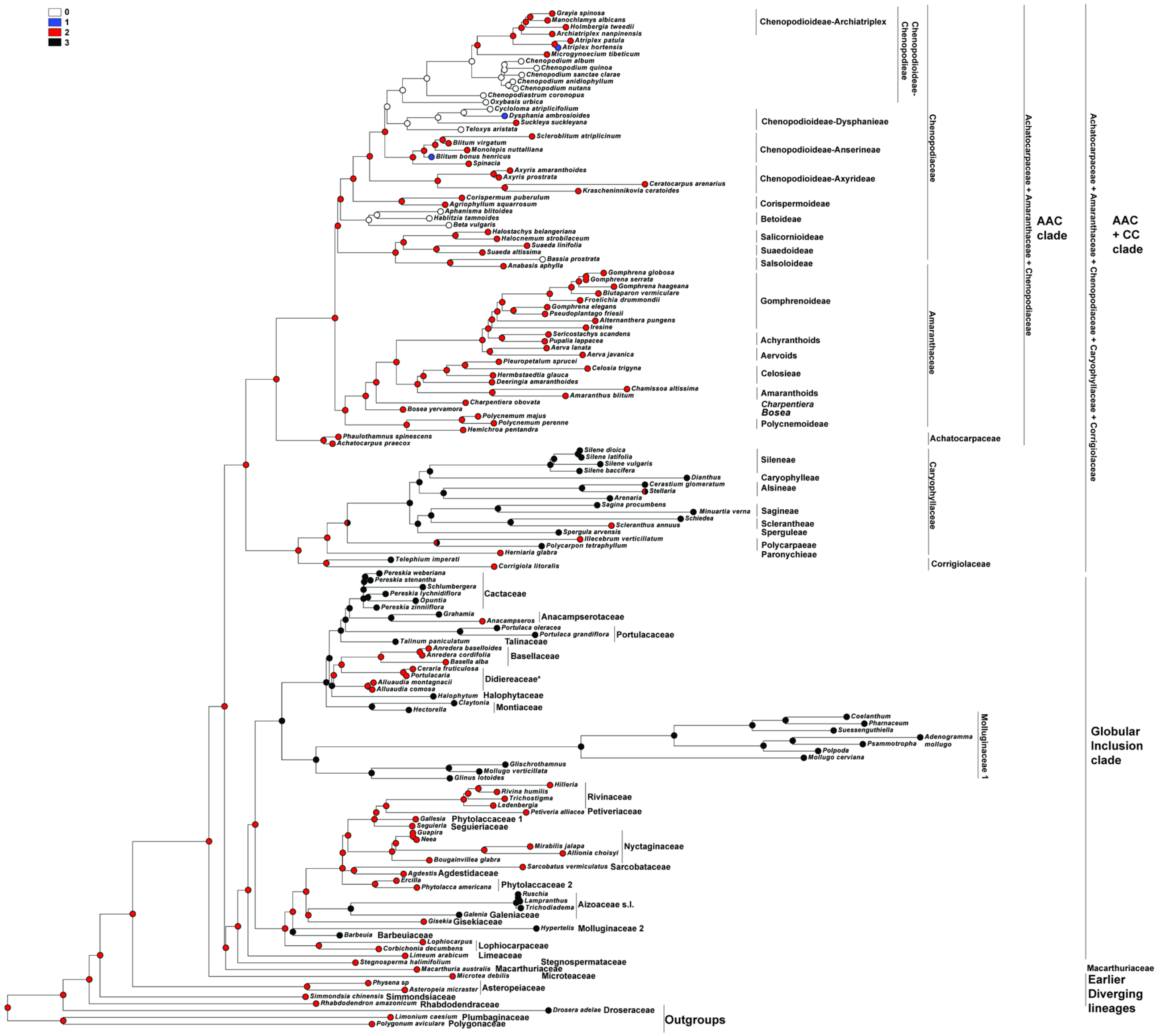

Fig 13. MP reconstruction of the evolutionary history of embryo orientation of combined plastid dataset. Character states: 0—horizontal; 1—both vertical and horizontal within individual (spatial heterospermy); 2—vertical; 3—not applicable due to more than two seeds in the fruit or locule. Morphological characters treated as unordered.

doi:10.1371/journal.pone.0117974.g013

wing-like outgrowths of the pericarp that enable anemochory are rare and known only in Sarcobataceae, Seguieriaceae and some representatives of the Chenopodiaceae (subfam. Corispermoideae). Fleshy and coloured fruits evolved several times from dry fruits. The families Rhabdodendraceae, Simmondsiaceae, Physenaceae and Asteropeiaceae are set apart from the other core Caryophyllales in having a thick multilayered fruit wall that is divided into several topographic zones, as well as a distinct seed coat structure. These families also share carpological characters such as massive fruits and a lack of nutritive tissue (perisperm and endosperm) in the ripe seed. The first lineage in the core Caryophyllales with typical "centrospermous" fruit and seed-coat structure is (depending on the topology of the molecular trees) either the American Microteaceae or the Australian Macarthuriaceae. The U-shaped cells with fine-grain

crystalliferous content in the pericarp above the inner epidermis are mostly known in the Achatocarpaceae, Amaranthaceae, and Chenopodiaceae (AAC clade).

The hard seed-coat testa appears to be typical of the majority of the core Caryophyllales. The bar-thickening of the endotegmen layer can be added as a basic character for many core Caryophyllales. All the members with one-seeded fruits arranged their embryos vertically, and many Chenopodiaceae representatives of different taxonomic position evolved a horizontally oriented embryo as a synapomorphy.

Unique, taxonomically important carpological characters justifying family rank have been proposed for Achatocarpaceae (polystarch grains in the seeds) and Sarcobataceae (general pericarp structure), and also for Seguieriaceae and Petiveriaceae (pericarp structure), the latter previously being considered part of Phytolaccaceae. The presence of raphides in the pericarp and seed coats, which is hardly noticeable in the one-layered pericarp, and the seed perisperm that is divided into two parts, are the carpological peculiarities of the Nyctaginaceae. The majority of the small families (Lophiocarpaceae, Microteaceae, Rivinaceae) do not show relevant differences in their pericarp and seed coat structure, although some *Rivina* species are distinguished by acicular outgrowths of the seed-coat testa.

Supporting Information

S1 Appendix. Origin of the material used in the carpological investigation in the present article.
(DOC)

S1 Table. List of taxa with GenBank-EMBL accession numbers used in the analyses
(DOC)

S2 Table. Diversity of the carpological characters in one-seeded fruits. Abbreviation: n/a—character not applicable (characters 4, 6 and 24 in many-seeded fruits). For more, see text.
(XLSX)

Acknowledgments

The authors thank Wei Wang, Zhi Xia and an anonymous reviewer for their valuable comments that have improved the manuscript, I. Belyaeva (Royal Botanic Gardens, Kew), G. Harper (Royal Botanic Garden Edinburgh), A. C. Timonin, A. G. Devyatov, M. Kushunina (Lomonosov Moscow State University), Sergey A. Kolesnikov (Michurinsk) and anonymous reviewers for discussion of some parts of the paper, and the herbarium staff who allowed fruit material to be obtained for the research. The work was carried out partially with the support of grants from the Russian Fund for Basic Research (project 14-04-00136-a: phylogeny of Caryophyllales) and the Russian Scientific Foundation (project 14-50-00029: carpological investigations).

Author Contributions

Conceived and designed the experiments: AS EM. Performed the experiments: AS AK MN EM MS KD. Analyzed the data: AS AK EM. Contributed reagents/materials/analysis tools: MN AE SB. Wrote the paper: AS AK EM.

References

1. The Angiosperm phylogeny group (2009) An update of the Angiosperm Phylogeny Group classification for the orders and families of flowering plants: APG III. Bot J Linn Soc 161: 105–121.

2. Schäferhoff B, Müller KF, Borsch T (2009) Caryophyllales phylogenetics: diesentangling of Phytolaccaceae and Molluginaceae and description of Microteaceae as a new isolated family. Willdenowia 39: 209–228.
3. Cuénoud P, Savolainen V, Chatrou LW, Powell M, Grayer RJ, et al. (2002) Molecular phylogenetics of Caryophyllales based on nuclear 18S rDNA and plastid rbcl, atbP, and matK DNA sequences. Am J Bot 89: 132–144. doi: 10.3732/ajb.89.1.132 PMID: 21669721
4. Brockington SF, Alexandre R, Ramdial J, Moore MJ, Crawley S, et al. (2009) Phylogeny of the Caryophyllales sensu lato: Revisiting hypotheses on pollination biology and perianth differentiation in the core Caryophyllales. Int J Plant Sci 170: 627–643.
5. Brockington SF, Dos Santos P, Glover B, Ronse de Craene LP (2013) Androecial evolution in Caryophyllales in light of paraphyletic Molluginaceae. Am J Bot 100: 1757–1778. doi: 10.3732/ajb.1300083 PMID: 24008516
6. Rettig JH, Wilson HD, Manhart JR (1992) Phylogeny of the Caryophyllales—gene sequence data. Taxon 41: 201–209.
7. Clement J, Mabry T, Wyler H, Dreiding A (1994) Chemical review and evolutionary significance of the betalains. In: Behnke HD, Mabry T, editors. Caryophyllales: evolution and systematic. Berlin: Springer. pp.247–261.
8. Brockington SF, Walker RH, Glover BJ, Soltis PS, Soltis DE (2011) Complex pigment evolution in the Caryophyllales. New Phytol 190: 854–864. PMID: 21714182
9. Solereder H (1899) Anatomie der Dicotyledonen. Stuttgart: Ferdinand Enke. 984 p.
10. Timonin AK (2011) Anomalous secondary thickening of the root and stem in Centrosperms: Specific of morphofuntional plant evolution. Moscow: KMK Press (in Russ.).
11. Behnke HD, Turner BL (1971) On specific sieve-tube plastids in Caryophyllales: Further investigations with special reference to the Bataceae. Taxon 20: 731–737.
12. Mabry TJ (1977) The order Centrospermae. Ann Mo Bot Gard 64: 210–220.
13. Behnke HD (1993) Further studies of the sieve-element plastids of the Caryophyllales including *Barbeuia*, *Corrigiola*, *Lyallia*, *Microtea*, *Sarcobatus*, and *Telephium*. Plant Syst Evol 186: 231–243.
14. Ronse de Craene L (2013) Reevaluation of the perianth and androecium in Caryophyllales: implications for flower evolution. Plant Syst Evol 299: 1599–1636.
15. Veselova TD, Timonin AK (2009) *Pleuropetalum* Hook. fil. is still an anomalous member of Amaranthaceae Juss. An embryological evidence. Wulfenia 16: 99–116.
16. Nowicke JW, Skvarla JJ (1977) Pollen morphology and the relationship of the Plumbaginaceae, Polygonaceae, and Primulaceae to the order Centrospermae. Smithson Contrib Bot 37: 1–64.
17. Nowicke JW (1996) Pollen morphology, exine structure and the relationships of Basellaceae and Didiereaceae to Portulacaceae. Syst Bot 21: 187–208.
18. Maheshwari H, Pullaiah T (1975) Life history of *Basella rubra* L. and taxonomic status of the family Basellaceae. J Indian Bot Soc 54: 154–166.
19. Netolitzky F (1926) Anatomie der Angiospermen-Samen. In: Linsbauer K, editor. Handbuch der Pflanzenanatomie, vol. 10. Berlin: Verlag Gebr. Bornträger. pp 1–364.
20. Wunderlich R (1967) Some remarks on the taxonomic significance of the seed coat. Phytomorphology 17: 301–311.
21. Corner EJH (1976) The seed of Diocotyledons. Cambridge: Univ. Printing House. 324 p.
22. Rocen T (1927) Zur Embryologie der Centrospermen. D. Ph. Thesis, Uppsala, Appelbergs.
23. Takhtajan AL, editor. (1991) Anatomia seminum comparativa, vol. 3. St.-Petersburg: Nauka (in Russ.).
24. Hill TG, de Fraine E (1912) On the seedling structure of certain Centrospermae. Ann Bot 26: 175–199.
25. Eckardt T (1954) Morphologische und systematische Auswertung der Placentation von Phytolaccaceen. Ber Dtsch Bot Ges 67: 113–128.
26. Eckardt T (1955) Nachweis der Blattbürtigkeit („Phyllosporie") grundständiger Samenanlagen bei Centrospermen. Ber Dtsch Bot Ges 68: 167–182.
27. Moquin-Tandon A (1849) Basellaceae. In: De Candolle A. Prodromus systematis naturalis regni vegetabilis 13(2). Paris: Masson. pp. 220–230.
28. Volkens G (1893) Chenopodiaceae, Basellaceae. In: Engler A, Prantl K, editors. Die Natürlichen Pflanzenfamilien, vol. 3. Leipzig: Engelmann Verlag. pp. 36–91; 124–128.
29. Bentham G, Hooker JD (1880) Genera plantarum 3(1). Reeve & Co., London, UK.

30. Rousi A, Yli-Rekola M, Jokela P, Kalliola R, Pietilä L, et al. (1988) The fruit of *Ullucus* (Basellaceae), an old enigma. Taxon 37: 71–75.

31. Sukhorukov AP (2008) Fruit anatomy of *Anabasis* (Salsoloideae, Chenopodiaceae). Aust Syst Bot 21: 431–442.

32. Lindley J (1845) A note upon the genus *Sarcobatus* Nees. London J Bot 4: 1–3.

33. Seubert M (1844) Ueber *Sarcobatus Maximiliani* Nees. Bot Zeitung 2: 753–755.

34. Baillon MH (1887) Développement de la fleur femelle du *Sarcobatus*. Bull Mens Soc Linn Paris 1: 649.

35. Nelson A (1909) New Manual of botany of the Central Rocky Mountains (Vascular plants), 2nd ed. New York-Cincinnati-Chicago: American Book Co. 662 p.

36. Grubov VI (1980) Chenopodiaceae. In: Takhtajan AL, editor. Plant Life 5(1). Moscow: Prosveschenie. pp. 374–382. (in Russ).

37. Hils MH, Thieret JW, Morefield JD (2003) Sarcobatus. In: Flora of North America Editorial Committee, editor. Flora of North America, North of Mexico, vol. 4. New York: Oxford University Press. pp. 387–389.

38. Vierhapper F (1907) Die systematische Stellung der Gattung *Scleranthus*. Oesterr Bot Zeitschrift 57: 41–47; 91–96.

39. Rohweder O (1970) Centrospermen-Studien 4. Morphologie und Anatomie der Blüten, Früchte und Samen bei Alsinoideen und Paronychioideen s. lat. (Caryophyllaceae). Bot Jahrb Syst 90: 201–271.

40. Devyatov AG, Ermilova IY (2002) The change of the fruit and seed structure in connection with origin of single-seeded diaspores in the tribe Caryophylleae. Bull Soc Nat Mosc, sect. biol. 107: 69–73 (in Russ).

41. Devyatov AG, Korolkova EO, Polevova SV (2003) Change of the morpho-anatomical structure of fruits and seeds in the tribe Paronychieae (Caryophyllaceae) in connection with origin of single-seeded fruits. Bull Soc Nat Mosc, sect. biol. 108: 67–70 (in Russ).

42. Friedrich HC (1956) Studien über die natürliche Verwandschaft der Plumbaginales und Centrospermae. Phyton (Horn) 6: 220–263.

43. Greenberg AK, Donoghue MJ (2011) Molecular systematics and character evolution in Caryophyllaceae. Taxon 60: 1637–1652.

44. Sukhorukov (Suchorukow) AP (2005) Karpologische Untersuchung der *Axyris*-Arten (Chenopodiaceae) im Zusammenhang mit ihrer Diagnostik und Taxonomie. Feddes Repert 116: 168–176.

45. Sukhorukov AP (2006) Zur Systematik und Chorologie der in Russland und benachbarten Staaten (in den Grenzen der ehemaligen UdSSR) vorkommenden Atriplex-Arten (Chenopodiaceae). Ann Naturhist Mus Wien 108 B: 307–420.

46. Sukhorukov AP (2007) Fruit anatomy and its taxonomic significance in *Corispermum* (Corispermoideae, Chenopodiaceae). Willdenowia 37: 63–87.

47. Sukhorukov AP (2011) *Axyris* (Chenopodiaceae s.str. or Amaranthaceae s.l.) in the Himalayas and Tibet. Willdenowia 41: 75–82.

48. Sukhorukov AP (2012) Taxonomic notes on *Dysphania* and *Atriplex* (Chenopodiaceae). Willdenowia 42: 169–180.

49. Sukhorukov AP, Zhang M (2013) Fruit and seed anatomy of *Chenopodium* and related genera (Chenopodioideae, Chenopodiaceae/Amaranthaceae): Implications for evolution and phylogeny. PLoS ONE 8(4). e61906. doi: 10.1371/journal.pone.0061906 PMID: 23626750

50. Sukhorukov AP, Zhang M, Nilova MV (2014) The carpology and taxonomy of some Chinese *Corispermum* (Amaranthaceae s.l.). Phytotaxa 172: 81–93.

51. Schiman-Czeika H (1987) Notes on the capsule dehiscence in *Acanthophyllum* (Caryophyllaceae) and allied genera. Plant Syst Evol 155: 67–69.

52. Werker E (1997) Seed anatomy. Berlin & Stuttgart: Bornträger Verlag. 424 p.

53. Stamatakis A (2006) RAxML-VI-HPC: maximum likelihood-based phylogenetic analyses with thousands of taxa and mixed models. Bioinformatics 22: 2688–2690. PMID: 16928733

54. Ronquist F, Huelsenbeck JP (2003) MrBayes 3: Bayesian phylogenetic inference under mixed models. Bioinformatics 19: 1572–1574. PMID: 12912839

55. Crowl AA, Mavrodiev E, Mansion G, Haberle R, Pistarino A, et al. (2014) Phylogeny of Campanuloideae (Campanulaceae) with Emphasis on the Utility of Nuclear Pentatricopeptide Repeat (PPR) Genes. PLoS ONE 9(4): e94199. doi: 10.1371/journal.pone.0094199 PMID: 24718519

56. Maddison WP, Maddison DR (2011) Mesquite: a modular system for evolutionary analysis. Version 2.75. http://mesquiteproject.org

57. Prance GT (1968) The systematic position of *Rhabdodendron* Gilg & Pilg. Bull Jard Bot Nat Belg 38: 127–146.

58. Prance GT (1972) Rhabdodendraceae. Flora Neotropica Monograph, vol. 11. New York: Hafner Co. 22 p.

59. Puff C, Weber A (1976) Contributions to the morphology, anatomy, and karyology of *Rhabdodendron*, and a reconsideration of the systematic position of the Rhabdodendronaceae. Plant Syst Evol 125: 195–222.

60. Behnke HD (1977) Zur Skulptur der Pollen-Exine bei drei Centrospermen (*Gisekia*, *Limeum*, *Hectorella*), bei Gyrostemonaceen und Rhabdodendraceen. Plant Syst Evol 128: 227–235.

61. Wolter-Filho W, da Rocha AI, Yoshida M, Gottlieb OR (1989) Chemosystematics of *Rhabdodendron*. Phytochemistry 28: 2355–2357.

62. Köhler E (2003) Simmondsiaceae. In: Kubitzki K, Bayer C, editors. The families and genera of vascular plants, vol. 5. Berlin–Heidelberg–New York: Springer. pp 355–358.

63. Müller J (1869) Buxaceae. In: De Candolle A Prodromus systematis naturalis regni vegetabilis 16(1). Paris: C. Lahure. pp 9–23.

64. Reveal JL (1992) Validation of ordinal names of extant vascular plants. Novon (St Louis) 2: 238–240.

65. Fay MF, Cameron KM, Prance GT, Lledó MD, Chase MW (1997) Familial relationships of *Rhabdodendron* (Rhabdodendraceae): Plastid rbcl sequences indicate a Caryophyllid placement. Kew Bull 52: 923–932.

66. Meimberg H, Dittrich P, Bringmann G, Schlauer J, Heubl G (2000) Molecular phylogeny of Caryophyllidae s.l. based on *matK* sequences with special emphasis of carnivorous taxa. Plant Biol 2: 218–228.

67. Melikyan AP (1968) Position of the Buxaceae and Simmondsiaceae families in the system. Bot Z (Bot Zhurn) 53: 1043–1047 (in Russ).

68. Schmid R (1978) Floral and fruit anatomy of jojoba (*Simmondsia chinensis*). In aninymous, Memorias de la II Conferencia Internacional sobre la Jojoba y su aprovechamiento (Febr. 1976). Mexico: Consejo Nacional de Ciencia & Tecnologia. pp 143–148.

69. Behnke HD (1982) Sieve-element plastids, exine sculpturing and the systematic affinities of Buxaceae. Plant Syst Evol 139: 257–266.

70. Thakur HA, Patil DA (2011) Taxonomic and phylogenetic assessment of the Euphorbiaceae: A review. JES Life Sciences 2: 37–46.

71. Carlquist S (2002) Wood anatomy and successive cambia in *Simmondsia* (Simmondsiaceae): Evidence for inclusion in Caryophyllales s.l. Madrono 49: 158–164.

72. Bobrov AVFCh, Melikyan AP, Romanov MS (2009) Morphogenesis of fruits of Magnoliophyta. Moscow: Librocom. 400 p (in Russ).

73. Wiger J (1935) Embryological studies on the families Buxaceae, Meliaceae, Simaroubaceae and Burseraceae. D.Ph. Thesis, Lund.

74. Gentry HS (1958) The natural history of Jojoba (*Simmondsia chinensis*) and its cultural aspects. Econ Bot 12: 261–295.

75. Morton CM, Karol KG, Chase MW (1997) Taxonomic affinities of *Physena* (Physenaceae) and *Asteropeia* (Theaceae). Bot Rev 63: 231–239.

76. Carlquist S (2006) *Asteropeia* and *Physena* (Caryophyllales): A case study in comparative wood anatomy. Brittonia 58: 301–313.

77. Takhtajan AL (2009) Flowering plants. 2nd ed. Leipzig: Springer. 871 p.

78. Christenhusz MJM, Brockington SF, Christin PA, Sage RF (2014) On the disintergration of Molluginaceae: a new genus and family (*Kewa*, Kewaceae) segregated from *Hypertelis*, and placement of *Macarthuria* in Macarthuriaceae. Phytotaxa 181: 238–242.

79. Lepschi BJ (1996) A taxonomic revision of *Macarthuria* (Molluginaceae) in Western Australia. Nuytsia 11: 37–54.

80. Behnke HD, Mabry TJ, Neuman P, Barthlott W (1983) Ultrastructural, micromorphological and phytochemical evidence for a "central position" of *Macarthuria* (Molluginaceae) within the Caryophyllales. Plant Syst Evol 143: 151–161.

81. Endress ME, Bittrich V (1993) Molluginaceae. In: Kubitzki K, Rohwer JG, Bittrich V, editors. The families and genera of vascular plants, vol. 2. Berlin–Heidelberg–New York: Springer. pp 419–425.

82. Christin PA, Sage TL, Edwards EJ, Ogburn RM, Khoshravesh R, et al. (2011) Complex evolutionary transitions and the significance of C_3-C_4 intermediate forms of photosynthesis in Molluginaceae. Evolution 65: 643–660. doi: 10.1111/j.1558-5646.2010.01168.x PMID: 20955197

83. Kunth KS (1817) Nova genera et species plantarum, vol. 2, Paris: D'Hautel.
84. Bittrich V (1993) Caryophyllaceae. In: Kubitzki K, Rohwer JG, Bittrich V, editors. The families and genera of vascular plants, vol. 2. Berlin–Heidelberg–New York: Springer. pp 206–236.
85. Carlquist S (1995) Wood anatomy of Caryophyllaceae: Ecological, habital, systematic, and phylogenetic implications. Aliso 14: 1–17.
86. Smissen RD, Clement JC, Garnock-Jones PhJ, Chambers GK (2002) Subfamilial relationships within Caryophyllaceae as inferred from 5'ndhF sequences. Am J Bot 89: 1336–1341. doi: 10.3732/ajb.89.8.1336 PMID: 21665736
87. Fior S, Karis PO, Casazza G, Minuto L, Sala F (2006) Molecular phylogeny of the Caryophyllaceae (Caryophyllales) inferred from chroloplast matK and nuclear rDNA ITS sequences. Am J Bot 93: 399–411. doi: 10.3732/ajb.93.3.399 PMID: 21646200
88. Shulz VA (1989) The genus *Saponaria* L. in the flora of USSR. Riga: Zinatne. 127 p (in Russ).
89. Harbaugh DT, Nepokroeff N, Rabeler RK, McNeill J, Zimmer EA, et al. (2010). A new lineage-based tribal clasification of the family Caryophyllaceae. Int J Plant Sci 171: 185–198.
90. Dumortier BC (1829) Analyse des familles des plantes: avec l'indication des principaux genres qui s'y rattachent. Tournai: Casterman.106 p.
91. Ikonnikov SS (1995) The genus *Corrigiola* (Illecebraceae). Bot Z (Bot Zhurn) 80: 113–118 (in Russ).
92. Rohweder O, Urmi E (1978) Centrospermen-Studien 10. Untersuchungen über den Bau der Blüten und Früchte von *Cucubalus baccifer* L. und *Drypis spinosa* L. Bot Jahrb Syst 100: 1–25.
93. Rohweder O, Urmi E (1971) Centrospermen-Studien 5. Bau der Blüten, Früchte und Samen von *Pteranthus dichotomus* Forsk. (Caryophyllaceae). Bot Jahrb Syst 90: 447–468.
94. Pal N (1952) A contribution to the life-histories of *Stellaria media* Linn. and *Polycarpon loeflingiae* Benth. & Hook. Proc Indian Natl Sci Acad B Biol Sci 18: 363–378.
95. Walter H (1906) Die Diagramme der Phytolaccaceen. Bot Jahrb Syst 37 (Beiblatt 85): 1–57.
96. Walter H (1909) Phytolaccaceae. In: Engler A, editor. Das Pflanzenreich 39. Leipzig: Engelmann Verlag. pp 1–154.
97. Carlquist S (2000) Wood and bark anatomy of Achatocarpaceae. SIDA Contrib. Bot. 19: 71–78.
98. Heimerl A (1934) Nyctaginaceae; Achatocarpaceae. In: Engler A, Harms H, editors. Die Natürlichen Pflanzenfamilien, 2nd ed. Leipzig: Engelmann Verlag. pp 86–134; 174–178.
99. Bortenschlager S, Auinger A, Blaha J, Simonsberger P (1972) Pollen morphology of the Achatocarpaceae (Centrospermae). Ber Naturwiss-Med Vereins Innsbruck 59: 7–14.
100. Skvarla JJ, Nowicke JW (1982) Pollen fine structure and relationships of *Achatocarpus* Triana and *Phaulothamnus* A. Gray. Taxon 31: 244–249.
101. Kadereit G, Zacharias E, Mavrodiev E, Sukhorukov AP (2010) Molecular phylogeny of Atripliceae (Chenopodioideae, Chenopodiaceae): Implications for systematics, biogeography, flower and fruit evolution, and the origin of C_4 photosynthesis. Am J Bot 97: 1664–1687. doi: 10.3732/ajb.1000169 PMID: 21616801
102. Kadereit G, Borsch T, Weising K, Freitag H (2003) Phylogeny of Amaranthaceae and Chenopodiaceae and the evolution of C4 photosynthesis. Int J Plant Sci 164: 959–986.
103. Costea M, Waines G, Sanders A (2001) Structure of the pericarp in some *Amaranthus* L. (Amaranthaceae) species and its taxonomic significance. Aliso 20: 51–60.
104. Oyama SO, de Souza LA, Muneratto JC, Meyer Albiero AL (2010) Morphological and anatomical features of the flowers and fruits during the development of *Chamissoa altissima* (Jacq.) Kunth (Amaranthaceae). Braz Arch Biol Technol 53: 1425–1432.
105. Butnik AA, Zhapakova UN (1991) Amaranthaceae. In: Takhtajan AL, editor. Anatomia seminum comparativa III. Leningrad: Nauka. pp 74–77 (in Russ).
106. Müller K, Borsch T (2005) Phylogenetics of Amaranthaceae based on matK/trnK sequnce data: evidence from parsimony, likelihood, and Bayesian analyses. Ann Mo Bot Gard 92: 66–102.
107. Sage RF, Sage LT, Pearcy RW, Borsch T (2007) The taxonomic distribution of C_4 photosynthesis in Amaranthaceae sensu stricto. Am J Bot 94: 1992–2003. doi: 10.3732/ajb.94.12.1992 PMID: 21636394
108. Ogundipe OT, Chase M (2009) Phylogenetic analysis of Amaranthaceae based on matK DNA sequence data with emphassis on West African species. Turk J Botany 33: 153–161.
109. Masson R, Kadereit G (2013) Phylogeny of Polycnemoideae (Amaranthaceae): Implications for biogeography, character evolution and taxonomy. Taxon 62: 100–111.

110. Meikle RD (1985) Flora of Cyprus, vol. 2. London: Bentham-Moxon Trust, Royal Botanic Gardens Kew. 1969 p.

111. Townsend CC (1993) Amaranthaceae. In: Kubitzki K, Rohwer JG, Bittrich V, editors. The families and genera of vascular plants, vol. 2. Berlin–Heidelberg–New York: Springer. pp 7–91.

112. Bramwell D (1976). The systematic position of the genus *Bosea* L. (Amaranthaceae). Bot Macaronésica 2: 19–24.

113. Kull U, Kühn B (1978) On some constituents of *Bosea yervamora* fruits. Vieraea 8: 187–190.

114. Kunkel G (1975) Additions and corrections to Eriksson O, Hansen A, Sunding P: "Flora of Macaronesia. Checklist of vascular plants 1974". Cuadernos Bot Canaria 24: 29–59.

115. Plisko MA (1996) Anacardiaceae. In: Danilova MF, editor. Anatomia seminum comparativa, vol. 5. St.-Petersburg: Mir & Semya. pp 445–469 (in Russ).

116. Sohmer SH (1972) Revision of the genus *Charpentiera* (Amaranthaceae). Brittonia 24: 283–312.

117. Sohmer SH (1973) A preliminary report of the biology of the genus *Charpentiera* (Amaranthaceae). Pac Sci 27: 399–405.

118. Schinz H (1893) Amaranthaceae. In: Engler A, Prantl K, editors. Die Natürlichen Pflanzenfamilien, vol. 3. Leipzig: Engelmann Verlag. pp 91–118.

119. Applequist WL, Pratt DB (2005) The Malagasy endemic *Dendroportulaca* (Portulacaceae) is referable to *Deeringia* (Amaranthaceae): molecular and morphological evidence. Taxon 54: 681–687.

120. Ulbrich E (1934) Chenopodiaceae. In: Engler A, Harms H, editors. Die Natürlichen Pflanzenfamilien, 2nd ed., vol. 16. Leipzig: Engelmann Verlag. pp 379–584.

121. Sukhorukov AP (2010) Heterodiaspory and its correlation with heteroanthocarpy and fruit/seed dimorphisms (a case study of some Chenopodiaceae representatives). Bull Soc Nat Mosc, sect. biol. 115: 39–47.

122. Behnke HD (1976) Ultrasculpture of sieve-element plastids in Caryophyllales (Centrospermae), evidence for the delimitation and classification of the order. Plant Syst Evol 126: 31–54.

123. Nowicke JW, Skvarla JJ (1979) Pollen morphology: The potential influence in higher order systematic. Ann Mo Bot Gard 66: 633–700.

124. Borsch T (1998) Pollen types in Amaranthaceae. Morphology and evolutionary significance. Grana 37: 129–142.

125. Struwig M, Jordaan A, Siebert SJ (2011) Anatomical adaptations of *Boerhavia* L. and *Commicarpus* Standl. (Nyctaginaceae) for survival in arid environments of Namibia. Acta Biol Crac, Ser Bot 53: 50–58.

126. Akhani H, Edwards G, Roalson EH (2007) Diversification of the Old World *Salsoleae* s.l. (*Chenopodiaceae*): Molecular phylogenetic analysis of nuclear and chloroplast data sets and a revised classification. Int J Plant Sci 168: 931–956.

127. Metcalfe CR, Chalk L (1950) Anatomy of the Dicotyledons, vol. 1–2. Oxford: Clarendon Press. 1500 p.

128. Franchet AR (1882) Faune et flore des pays Çomalis (Afrique orientale). Paris: Challamel Ainé. 70 p.

129. Kung H, Chu [Zhu] GL, Tsien CP, Li A, Ma C (1978) The Chenopodiaceae in China. Acta Phytotax Sin 16: 99–123.

130. Zhu GL (1979) Chenopodiaceae. In: Kung HW, Tsien CP, editors. Flora Reipublicae Popularis Sinicae, vol. 25. Beijing: Science Press. pp 115–135.

131. Zhu GL (1988) On systematic position of *Baolia* Kung et G. L. Zhu in Chenopodiaceae. Acta Phytotax Sin 26: 299–300.

132. Iljin MM (1936) Chenopodiaceae. Pp. In: Komarov VL, editor. Flora of USSR, vol. 6. Moscow-Leningrad: Akademiya Nauk. pp 2–354.

133. Behnke HD (1997) Sarcobataceae—a new family of Caryophyllales. Taxon 46: 495–507.

134. Falkovitsh MI, Kovalev OV (2007) An overview of classification of the higher taxa of the Chenopodiaceae and the family origin. In: Kovalev OV, Zhilin SG, editors. Phase transitions in biological systems and the evolution of biodiversity. St.-Petersburg: PIYAF RAN (in Russ.). pp 80–115.

135. Kamelin RV (2011) Ancient xerophilous family Chenopodiaceae in the Turanian and Central Asian flora. Bot Z (Bot Zhurn) 96: 441–464 (in Russ.).

136. Britton N, Brown A (1913) An illustrated flora of the Northern United States, Canada and the British possession, 2nd ed. New York: Charles Scribner's Sons.

137. Butnik AA (1981) The carpological characteristics of the *Chenopodiaceae* representatives. Bot Z (Bot Zhurn) 66: 1433–1433 (in Russ.).

138. Butnik AA (1991) Family *Chenopodiaceae*. In: Takhtajan AL, editor. *Anatomia* seminum comparativa III. Leningrad: Nauka. pp 77–82 (in Russ.).

139. Meikle RD (1979) Supplementary notes on *Commicarpus* (Nyctaginaceae). Kew Bull 34: 341–343.

140. Miller AG (1996) Nyctaginaceae. In: Miller AG, Cope TA, Nyberg JA, editors. Flora of the Arabian Peninsula and Socotra, vol. 1. Edinburgh: Univ. Press. pp 143–155.

141. Grubert M (1982) Bestimmung des Schleimgehaltes myxospermer Diasporen verschiedener Angiospermenfamilien. Plant Syst Evol 141: 7–21.

142. Le Duc A (1994) A revision of *Mirabilis* section *Mirabilis* (Nyctaginaceae). SIDA Contrib. Bot. 16: 613–648.

143. Mahrt M, Spellenberg R (1994) Taxonomy of *Cyphomeris* (Nyctaginaceae) based on multivariate analyses of geographic variation. SIDA Contrib. Bot. 16: 679–697.

144. Standley PC (1911) The Allioniaceae in of Mexico and Central America. Contributions from the United States National Herbarium. Smithsonian Institution 13: 377–430.

145. Bogle AL (1974) The genera of Nyctaginaceae in the Southeastern United States. J Arnold Arbor 55: 1–37.

146. Struwig M, Jordaan A, Siebert SJ (2011) Anatomy of the Southern African *Boerhavia* and *Commicarpus* species (Nyctaginaceae). Bangladesh J Plant Taxon 18: 105–115.

147. Douglas NA, Manos PS (2007) Molecular phylogeny of Nyctaginaceae: Taxonomy, biogeography, and characters associated with a radiation of xerophytic genera in North America. Am J Bot 94: 856–872. doi: 10.3732/ajb.94.5.856 PMID: 21636455

148. Kajale LB (1938) Embryo and seed development in the Nyctaginaceae I. Studies in the genus *Boerhavia*. J Indian Bot Soc 17: 243–254.

149. da Rosa SM, de Souza LA, Moscheta IS (2002) Morfo-anatomia do desenvolvimento do antocarpo e do fruto de *Pisonia aculeata* L. (Nyctaginaceae). Acta Cient Venez 53: 245–250. PMID: 12945489

150. Douglas NA, Spellenberg R (2010) A new tribal classification of Nyctaginaceae. Taxon 59: 905–910.

151. Wilson RC (1975) *Abronia*: III. Pericarp and seed coat anatomy and its ecological implications for nine species of *Abronia*. Aliso 8: 289–299.

152. Willson J, Spellenberg R (1977) Observations on anthocarp anatomy in the subtribe Mirabilinae (Nyctaginaceae). Madrono 24: 104–111.

153. Bittrich V, Kühn U (1993) Nyctaginaceae. In: Kubitzki K, Rohwer JG, Bittrich V, editors. The families and genera of vascular plants, vol. 2. Berlin–Heidelberg–New York: Springer. pp 473–486.

154. López HA, Caletto L (2002) Flower structure and reproductive biology of *Bougainvillea stipitata* (Nyctaginaceae). Plant Biol 4: 508–514.

155. Chen SH, Wu MJ (2007) A taxonomical study of the genus *Boerhavia* (Nyctaginaceae) in Taiwan. Taiwania. 52: 332–342.

156. Chew S (2010) Anatomical features of *Bougainvillea* (Nyctaginaceae). SURG (Studies by Undergraduate Researchers at Guelph) 4: 72–78.

157. Poddubnaya-Arnoldi VA (1976) Cytoembryology of Angiosperms. Basis and Perspectives. Moscow: Nauka. 507 p (in Russ.).

158. Franz E (1908) Beiträge zur Kenntnis der Portulacaceen und Basellaceen. Bot Jahrb Syst 42, Beiblatt 97: 1–46.

159. Lacroix Ch (1986) Floral development of *Basella rubra* L. (Basellaceae). M Sc Thesis, Univ. McGill.

160. Perrier de la Bâthie H (1950) Basellaceae. In: Humbert H, editor. Flore de Madagascar et des Comores (Plantes vasculaires), vol. 72-76: Basellacceae-Ranunculaceae. Paris: Firmin-Didot & Co. pp 1–7.

161. Nyffeler R, Eggli U (2010) Disintegration of Portulacaceae: a new familial classification of the suborder Portulacineae (Caryophyllales) based on molecular and morphological data. Taxon 59: 227–240.

162. Nyananyo BL, Olowokudejo JD (1986) The taxonomic position of *Ceraria* Pears. & Steph., *Portulacaria* Jacq., and *Silvaea* Phil. Feddes Repert 97: 775–777.

163. Applequist WL, Wallace RS (2000) Phylogeny of the Madagascan endemic family Didiereaceae. Plant Syst Evol 221: 157–166.

164. Applequist WL, Wallace RS (2003) Expanded circumscription of Didiereaceae and its division into three subfamilies. Adansonia 25: 13–16.

165. McNeill J (1974) Synopsis of a revised classification of the Portulacaceae. Taxon 23: 725–728.

166. Brown GK, Varadarajan GS (1985) Studies in Caryophyllales. I. Re-evaluation and classification of Phytolaccaceae s.l. Syst Bot 10: 49–63.

167. Downie SR, Palmer JD (1994) A chloroplast DNA phylogeny oft he Caryophyllales based on structural and inverted repeat restrictionsite variation. Syst Bot 19: 236–252.

168. Lee J, Kim SY, Park SH, Ali MA (2013) Molecular phylogenetic relationships among members of the Phytolaccaceae sensu lato inferred from internal transcribed spacer sequences of nuclear ribosomal DNA. Genet Mol Res 12: 4515–4525. doi: 10.4238/2013.February.28.15 PMID: 23479160

169. Jansen S, Ronse de Craene LP, Smets E (2000) On the wood and stem anatomy of *Monococcus echinophorus* (Phytolaccaceae s.l.). Syst Geogr Plants 70: 171–179.

170. Doweld A, Reveal JL (2008) New suprageneric names for vascular plants. Phytologia 90: 416–417.

171. Narayana HS, Lodha BC (1963) Embryology of *Orygia decumbens* Forsk. (Aizoaceae). Phytomorphology 13: 54–59.

172. Eckardt T (1974) Vom Blütenbau der Centrospermen-Gattung *Lophiocarpus* Turcz. Phyton (Horn) 16: 13–27.

173. Hakki MI (2013) On flower anatomy and embryology of *Lophiocarpus polystachyus* (Lophiocarpaceae). Willdenowia 43: 185–194.

174. Hassan NMS, Meve U, Liede-Schumann S (f.) (2005) Seed coat morphology of Aizoaceae-Sesuvioideae, Gisekiaceae and Molluginaceae and its systematic significance. Bot J Linn Soc 148: 189–206.

175. Carlquist S (1999) Wood and stem anatomy of *Stegnosperma* (Caryophyllales); phylogenetic relationship; nature of lateral meristems and successive cambial activity. IAWA J 20: 149–163.

176. Hofmann U (1973) Centrospermen-Studien: 6. Morphologische Untersuchungen zur Umgrenzung und Gliederung von Aizoaceen. Bot Jahrb Syst 93: 247–324.

177. Kamelina OP, Proskurina OB (1985) On embryology of *Stegnosperma halimifolium* (Stegnospermataceae). Subdivision of the embryological characters in the order Caryophyllales. Bot Z (Bot Zhurn) 70: 721–730 (in Russ.).

178. Hofmann U (1977) Centrospermen-Studien: 9. Die Stellung von *Stegnosperma* innerhalb der Centrospermen. Ber Dtsch Bot Ges 90: 39–52.

179. Kowal T (1953) A key for determination of the seeds of the genera *Chenopodium* L. and *Atriplex* L. Monogr Bot 1: 87–163 (in Polish).

180. Kowal T (1954) The morphological and anatomical features of the seeds of genus *Amaranthus* L. and keys for their determination. Monogr Bot 2: 162–193 (in Polish).

181. Kowal T (1961) Morphology and anatomy of the seeds in Portulacaceae Rchb. Monogr Bot 12: 3–34 (in Polish).

182. Heimerl A (1889) Phytolaccaceae In: Engler A, Prantl K, editors. Die Natürlichen Pflanzenfamilien, vol. 3. Leipzig: Engelmann Verlag. pp 1–14.

183. Pax F (1889) Caryophyllaceae In: Engler A, Prantl K, editors. Die Natürlichen Pflanzenfamilien, vol. 3, Leipzig: Engelmann Verlag. pp 61–94.

184. Joshi AC, Rao VS (1933) Floral anatomy of *Rivina humilis* L., and the theory of the carpel polymorphism. New Phytol 32: 359–363.

185. Kajale LB (1954) A contribution to the embryology of the Phytolaccaceae. II. Fertilization and the development of embryo, seed and fruit in *Rivina humilis* L. and *Phytolacca dioica* L. J Indian Bot Soc 23: 206–225.

186. Melikyan AP (1986) The comparative seedcoat anatomy of Phytolaccaceae and relatives in light of systematics and phylogeny. In: Tikhomirov VN, editor Sources of information in the phylogenetic systematic of plants. Moscow: Nauka (in Russ.). pp 54–56.

187. Zheng HC, Ma SW, Chai TY (2010) Ovular development and perisperm formation in *Phytolacca americana* (Phytolaccaceae) and their systematic significance in Caryophyllales. J Syst Evol 48: 318–325.

188. Volgin SA, Tikhomirov VN (1980) About the structural types of monocyclic syncarpous gynoecium of Angiosperms. Bull Soc Nat Mosc, sect. biol. 85: 63–74 (in Russ.).

189. Shamrov II (2012) Spatial-temporal co-ordination during ovary and ovule development in the lysicarpous gynoecium. In: Timonin AK, et al, editors. Caryophyllales: New insights into the phylogeny, systematics and morphological evolution of the order: Proceedings of the Symposium. Tula: Grif & Co. pp 32–36.

190. Rohweder O (1965) Centrospermen-Studien 1. Der Blütenbau bei *Uebelinia kiwuensis* T. C. E. Fries. Bot Jahrb Syst 83: 406–418.
